# Association between DMFT and primary headaches: a study based on the Rafsanjan cohort study

**DOI:** 10.1186/s12903-023-03815-0

**Published:** 2024-01-04

**Authors:** Fatemeh Ayoobi, Parvin khalili, Zahra Jamali, Sogand Esmaeil Moghaddam, Farimah Sardari, Alireza Vakilian

**Affiliations:** 1 Occupational Safety and Health Research Center, NICICO, World Safety Organization, Rafsanjan University of Medical Sciences, Rafsanjan, Iran; 2 Clinical Research Development Unit, Ali-Ibn Abi-Talib Hospital, Rafsanjan University of Medical Sciences, Rafsanjan, Iran; 3 Social Determinants of Health Research Center, Rafsanjan University of Medical Sciences, Rafsanjan, Iran; 4 Pistachio Safety Research Center, Rafsanjan University of Medical Sciences, Rafsanjan, Iran; 5 Student Research Committee, Rafsanjan University of Medical Sciences, Rafsanjan, Iran; 6 Department of Oral Medicine, Dental School, Rafsanjan University of Medical Sciences, Rafsanjan, Iran; 7 Neurology Department, School of Medicine, Non-Communicable Diseases Research Center, Rafsanjan University of Medical Sciences, Rafsanjan, Iran

**Keywords:** DMFT index, Headache, Oral health, Rafsanjan cohort study (RCS), Prospective epidemiological research studies in IrAN (PERSIAN)

## Abstract

**Background:**

Although primary headaches are common disorders, there is little research on the possible relationship between primary headaches and oral health (decayed, missing, and filled teeth: DMFT). The present study aims to investigate the relationship between the DMFT index and primary headaches.

**Method:**

This descriptive study was performed on 8682 cases from the Rafsanjani cohort population based on the Rafsanjani cohort study (RCS) and Oral Health Branch of the Rafsanjan Cohort Study (OHBRCS). Episodic primary headache (EPH) and chronic primary headache (CPH) of RCS patients who participated in OHBRCS were studied according to their DMFT score in comparison to nonprimary headache patients. Demographic characteristics and risk factors were compared in different groups. We used crude and multiple logistic regression analyses in this study.

**Results:**

The missing teeth were significantly higher in the CPH group than in the no CPH group (*P* < 0.001), and filled teeth were significantly higher in the EPH group than in the no EPH group (*P* < 0.001). In the crude model, there was a direct significant association between the prevalence of EPH and filled teeth total and > 5 filled teeth, and after adjusting for confounders, this relationship remained significant. The odds ratios of CPH were not associated with DMFT or its components in the adjusted models.

**Conclusion:**

Our study found a correlation between filled teeth and EPH cases, but no correlation between CPH and DMFT or its components.

**Supplementary Information:**

The online version contains supplementary material available at 10.1186/s12903-023-03815-0.

## Background

Primary headaches (PH) refer to headaches that occur repeatedly without a specific cause and are divided into episodic and chronic groups [1]. There are three types of chronic primary headaches (CPH) including migraine group, tension-type headache group (TTH), and trigeminal autonomic cephalalgia group (TACs) [2]. Secondary headaches are proven by having three criteria: first, the presence of headache; second, a causative disorder; and finally, proof of causation [3, 4], which are the boundaries of separation of primary and secondary headaches.

There are some related factors to PH such as sex, race, age, familial history, and hypertension but not as a cause for them [2, 5]. Moreover, dental disorders have been reported to induce prolonged headaches in patients [6].

Oral and dental health care should include safe, consistent, diverse, and quality care that does not cause other diseases. The most important indicators in dental health are decayed, missing, and filled teeth (DMFT) indexes, which indicate decayed teeth, stretched teeth due to decay, and filled teeth [7, 8]. Here, a question was asked regarding whether the DMFT score has a role in causing PH. Peskersoy et al. (2016) investigated the relationship between migraine and oral and dental diseases in 2001 patients [9]. The oral and dental examinations of the patients were evaluated based on the DMFT index. The results of this study showed that there was a strong relationship between the incidence of migraine and oral and dental diseases [9]. The study carried out in 2019 reported that oral and dental diseases could cause migraine-like headaches [10].

In a study of DMFT in five-year-old Children, a significant association was observed between the child’s headache episodes and DMFT [11]. Additionally, the results of another study in 2021 showed that the prevalence of caries was high in children with headaches [12].

In filled teeth as a part of the DMFT index, mercury was a factor suspicious for many complications in humans. Mercury is a toxic substance that can lead to symptoms such as headaches, mood disorders, and tremors [13]. Mercury fillings could cause these types of symptoms when the vapor is inhaled. Silver amalgam fillings were also popular due to their economic benefits. They were less expensive but had side effects. In addition to their aesthetic drawbacks, they can also cause allergies, headaches, and other issues [13]. Composite fillings were more expensive and safer in comparison to amalgam fillings [13]. According to many studies regarding different types of headaches among the adult and young population, the causes of headaches are still unclear.

### Aim

The study aimed to explore the relationships between DMFT and episodic (duration less than 15 days per month) and chronic primary headache (duration more than 15 days per month) (EPH&CPH) among the participants in the Rafsanjan cohort study.

## Methods

### Ethical approval and study population

This descriptive study was conducted using the data of the RCS and Oral Health Branch of the Rafsanjan Cohort Study (OHBRCS) [14]. The dental and oral health of the RCS participants was investigated in OHBRCS. This study was approved by the Ethics Committee of Rafsanjan University of Medical Sciences (Ethical codes: ID: IR.RUMS.REC.1400.183). Furthermore, guidelines for the report of observational studies in epidemiology (STROBE) were considered for performing this study. All methods were carried out according to relevant guidelines and regulations. All of the questionnaires prepared in the Farsi language were previously validated in the PERSIAN cohort study [15].

### Sample size calculation

RCS, as a part of Prospective Epidemiological Research Studies in Iran (PERSIAN) [15], recruited 10,000 subjects of both genders aged 35–70 years, living in four pre-determined districts of Rafsanjan (2500 participants from each site) including urban and suburban areas. A population of 10,000 individuals would be large enough to detect for detection of a relative risk of 2 for exposure, (α level of 0.05, β of 90%, for a given 10% prevalence). Given the target population of 10,000, the recruitment team invited 14,827 individuals until the target sample size was reached (participation rate: 67.44%) [15]. This sample size estimated by the PERSIAN Cohort Central Scientific Committee for each PERSIAN cohort site supports adequate statistical power. This study began in 2015. A total of 8,682 subjects from the adult cohort study participated in the OHBRCS. To ensure that all procedures were performed following the PERSIAN Cohort protocol, quality assurance (QA) and quality control (QC) measures have been implemented by central and local QA/QC teams.

### Participant inclusion and exclusion criteria

Inclusion criteria included residency in Rafsanjan for a minimum of nine months per year, having Iranian ethnicity (confirmation of having a national identity card), and aged 35–70 years old [16]. The exclusion criteria were not understanding the Farsi language, being illiterate, and incomplete medical history questionnaires, especially headaches and incomplete dental examinations (DMFT data).

### Study method

A team of 27 personnel trained in the Persian Cohort Center, including general interviewers, physicians, laboratory technicians, and specialized interviewers, collected data and stored biological samples by the PERSIAN cohort protocols [15]. Signed informed written consent was obtained from the subjects, every participant had a specific code in the RCS, and the names of the participants were unknown [16]. They were interviewed using a validated questionnaire containing demographic, socioeconomic, and anthropometric characteristics, medical history, and personal habits. The socioeconomic status of subjects was measured using the wealth score index (WSI). Cigarette and opium smoking and alcohol consumption were self-reported. All individuals who consumed approximately 200 ml of beer or 45 ml of liquor once a week for at least six months were considered alcohol drinkers [15]. Smoking was divided into nonsmokers, current smokers, and former smokers. The subject who used opium at least once a week for six months was defined as an opium user [17].

The EPH and CPH were determined through interviews with questions regarding episodic and chronic headaches. (I) Has episodic primary headaches? Experience of recurring transient headaches in a lifetime of less than 15 days in a month was considered EPH. (II) Has chronic primary headaches? Headaches occurring for at least 15 days in a month and continuing for at least three consecutive months were defined as CPH in the most common PH, which were TTH and migraines. Headaches associated with head or neck injury, cranial or cervical vascular disorders, nonvascular intracranial disorders, substance use or withdrawal, infection, and psychiatric disorders (somatization or psychotic disorders) were not considered EPH and CPH [18]. The sub-classification of PH types was not included in this interview.

A trained assistant who accompanied the dental specialists recorded the data. Three oral medicine specialists, one periodontist, and one general dentist evaluated dental and oral status. The DMFT score of the participants was determined using the results of clinical examination and calculation of the number of decayed (D), filled (F), and missing (M) teeth due to caries. In the DMFT index, “missing” refers to a tooth removed due to caries. A tooth having been extracted for orthodontic or trauma reasons, etc., does not signify a missing tooth in the DMFT index.

The final analysis included 8641 participants. The oral questionnaire contained factors related to oral health that were asked by trained interviewers. Tooth brushing was categorized into two groups (no, yes).

### Statistical analyses

Frequency (%) for categorical variables and mean (SD: standard deviation) for the quantitative variables were used, and baseline characteristics were compared across the groups of our study using chi-square (χ²) for categorical variables and t-test for continuous variables.

In addition, we used crude and multivariable (adjusted) logistic regression analyses to assess the relation between CPH or EPH and DMFT as dependent and independent variables, respectively. Crude and two adjusted models were used in the regression analysis. Based on subject matter knowledge and epidemiological studies, we identified the Confounder’s variables. Potential confounding variables were sequentially entered into models according to their hypothesized strengths of association with DMFT and headaches. Then, variables with a *p*-value < 0.25 were selected as confounders.

Adjusted model 1 included basic sociodemographic characteristics (age, gender, education years, and wealth score index). Model 2 adjusted for habits (smoking, opium, and alcohol consumption), teeth brushing, disease history (diabetes, hypertension), and family history of headache. All analyses were performed through State V.12. *P*-values < 0.05 and 95% confidence intervals were considered statistically significant.

## Results

### Demographic, medical and oral characteristics of participants

The study involved a total of 8641 adults aged 35–70 years old (4642 women and 3999 men). The mean age of the participants was 49.95 years, and there was a statistically significant difference in the mean age of the EPH and control groups (no EPH) (*p* < 0.001). Additionally, there were differences in gender, educational level, BMI, wealth score index, cigarette smoking, opium and alcohol consumption, hypertension, diabetes mellitus, family history of headache, and teeth brushing between EPH and no EPH groups (Table 1). Of note, the mean missing teeth (*P* = 0.002) and filled teeth (*P* < 0.001) were significantly different between the EPH and no EPH groups.

The prevalence of CPH in women was higher than that in men, and this difference was significant between the CPH and control groups (no CPH) (*P* < 0.001). In addition, there were differences in educational level, BMI, wealth score index, cigarette smoking, opium and alcohol consumption, hypertension, and family history of headache between the CPH and no CPH groups. The mean missed teeth was significantly higher in the CPH group than in the no CPH group (*P* = 0.013) (Table 1).

**Table 1:** Demographic features of participants with and without primary headache

Characteristics	All (n = 8641)	EPH (n = 2518)	NO EPH (n = 6123)	*P*-value	CPH (n = 642)	No CPH (n = 7999)	*P*-value
Age—year				< 0.001			0.339
Mean ± SD	49.95 ± 9.50	48.86 ± 9.20	50.40 ± 9.59		50.29 ± 9.44	49.92 ± 9.51	
Gender—no (%)				< 0.001			< 0.001
Male	3999 (46.28)	771 (30.62)	3228 (52.72)		173 (26.95)	3826 (47.83)	
Female	4642 (53.72)	1747 (69.38)	2895 (47.28)		469 (73.05)	4173 (52.17)	
Education—year				0.001			< 0.001
Mean ± SD	8.63 ± 5.03	8.30 ± 5.00	8.76 ± 5.03		7.38 ± 4.93	8.73 ± 5.02	
BMI				0.044			< 0.001
Mean ± SD	27.85 ± 4.85	28.02 ± 4.79	27.79 ± 4.87		28.67 ± 4.93	27.79 ± 4.84	
Wealth score index				0.002			< 0.001
Mean ± SD	0.03 ± 0.98	− 0.02 ± 1.01	0.06 ± 0.96		-0.12 ± 1.00	0.04 ± 0.97	
Cigarette smoking—no (%)				< 0.001			< 0.001
No	6482 (75.13)	2042 (81.23)	4440 (72.62)		524 (82.13)	5958 (74.57)	
Current	1396 (16.18)	297 (11.81)	1099 (17.98)		76 (11.91)	1320 (16.52)	
Former	750 (9.40)	175 (6.96)	575 (8.69)		38 (5.96)	712 (8.91)	
Opium consumption—no (%)				< 0.001			< 0.001
Yes	1954 (22.65)	415 (16.51)	1539 (25.18)		109 (17.08)	1845 (23.09)	
No	6673 (77.35)	2099 (83.49)	4574 (74.82)		529 (82.92)	6144 (76.91)	
Alcohol consumption—no (%)				< 0.001			0.017
Yes	857 (9.93)	186 (7.40)	671 (10.97)		46 (7.21)	811 (10.15)	
No	7771 (90.07)	2328 (92.60)	5443 (89.03)		592 (92.79)	7179 (89.85)	
Hypertension—no (%)				0.002			< 0.001
Yes	1946 (22.52)	622 (24.70)	1324 (21.62)		205 (31.93)	1741 (21.77)	
No	6695 (77.48)	1896 (75.30)	4799 (78.38)		437 (68.07)	6258 (78.23)	
Family history of headache—no (%)				< 0.001			< 0.001
Yes	2293 (26.54)	950 (37.73)	1343 (21.93)		272 (42.37)	2021 (25.27)	
No	6348 (73.46)	1568 (62.27)	4780 (78.07)		370 (57.63)	5978 (74.73)	
Diabetes mellitus—no (%)				0.011			0.125
Yes	1698 (19.65)	452 (17.95)	1246 (20.35)		141 (21.96)	1557 (19.46)	
No	6943 (80.35)	2066 (82.05)	4877 (79.65)		501 (78.04)	6442 (80.54)	
Teeth brushing—no (%)				< 0.001			0.958
Yes	6132 (70.96)	1873 (74.38)	4259 (69.56)		455 (70.87)	5677 (70.97)	
No	2509 (29.04)	645 (25.62)	1864 (30.44)		187 (29.13)	2322 (29.03)	
DMFT				0.06			0.06
Mean ± SD	21.31 ± 8.53	21.05 ± 8.36	21.42 ± 8.60		21.93 ± 8.64	21.26 ± 8.52	
Decayed teeth				0.177			0.141
Mean ± SD	3.08 ± 3.64	2.99 ± 3.51	3.11 ± 3.70		2.89 ± 3.60	3.09 ± 3.56	
Missing teeth				0.002			0.013
Mean ± SD	14.87 ± 11.00	14.28 ± 10.69	15.11 ± 11.12		15.90 ± 11.01	14.78 ± 11.00	
Filled teeth				< 0.001			0.185
Mean ± SD	3.37 ± 4.65	3.77 ± 4.96	3.21 ± 4.51		3.14 ± 4.63	3.39 ± 4.66	

Abbreviations: Decayed, Missing, and Filled teeth (DMFT); Chronic primary headache (CPH); Episodic primary headache (EPH); Body mass index (BMI).

### Primary headache and oral characteristics of participants

The results of the crude and multiple logistic regression analyses are presented in Table 2. The crude model included the DMFT score and its components. In this model, a significant relationship between filled teeth total and EPH was observed (OR: 1.03; 95% CI 1.02 to 1.04). Additionally, after adjusting for confounders, the association between EPH and total filled teeth was significant (OR: 1.02; 95% CI 1.01 to 1.03).

For further investigation, the total number of filled teeth was divided into four categories (no filled teeth, 1–2 filled teeth, 3–5 filled teeth, and > 5 filled teeth). In the crude model, there was a direct significant association between the prevalence of EPH and > 5 filled teeth compared with the reference group (OR: 1.30; 95% CI 1.16 to 1.46). These results showed that the odds of EPH increased in participants with > 5 filled teeth by approximately 17% and 18% in adjusted models 1 and 2, respectively, compared with the reference group.

After adjusting for confounding variables, the odds ratio of CPH was not associated with DMFT or its components (Table 2).

**Table 2:** Relationship between DMFT index and chronic and episodic primary headache

	Crude model OR (95%Ci)	Adjusted model 1 OR (95%Ci)^a^	Adjusted model 2 OR (95%Ci)^b^
EPH			
DMFT	0.99 (0.99-1.00)	1.00 (1.00-1.01)	1.00(0.99–1.01)
Decayed teeth	0.99 (0.98-1.00)	0.98 (0.97-1.00)	0.98(0.96-1.00)
Missing teeth	0.99 (0.99-1.00)	1.00 (1.00-1.01)	1.00(0.99–1.01)
Filled teeth total	1.03 (1.02–1.04) *	1.02 (1.01–1.03) *	1.02(1.01–1.03) *
No Filled teeth	1	1	1
Filled teeth = 1–2	1.12(0.97–1.29)	0.96(0.83–1.12)	0.97(0.83–1.13)
Filled teeth = 3–5	1.07(0.93–1.24)	0.96(0.82–1.13)	0.98(0.83–1.15)
Filled teeth > 5	1.30(1.16–1.46) *	1.17(1.02–1.34) *	1.18(1.02–1.36) *
CPH			
DMFT	1.01 (1.00-1.02)	1.01 (0.99–1.02)	1.01(0.99–1.02)
Decayed teeth	0.98 (0.96–1.01)	0.98 (0.96–1.01)	0.99(0.96–1.02)
Missing teeth	1.00 (1.00-1.02)	1.00 (1.00-1.02)	1.00(0.99–1.02)
Filled teeth total	0.99 (0.97–1.01)	0.99 (0.97–1.02)	1.00(0.98–1.02)
No Filled teeth	1	1	1
Filled teeth = 1–2	0.96(0.76–1.23)	0.93(0.72–1.19)	0.95(0.73–1.23)
Filled teeth = 3–5	0.80(0.61–1.04)	0.82(0.62–1.08)	0.85(0.64–1.13)
Filled teeth > 5	0.85(0.69–1.04)	0.89(0.70–1.13)	0.92(0.72–1.17)

The crude model is stratified on the status of DMFT.

The adjusted model 1 is adjusted for confounding variables age (continuous variable), gender (male/female), education years (continuous variable), and Wealth score index (continuous variable).

The adjusted model 2 has additional adjustments for confounding including diabetes (yes/no), hypertension (yes/no), Family history of headache (yes/no), smoking (yes/no), opium consumption (yes/no), Alcohol consumption (yes/no) and Teeth brushing (yes/no).

Abbreviations: Decayed, Missing, and Filled teeth (DMFT); Chronic primary headache (CPH); Episodic primary headache (EPH).

* Significant odds ratios

### Fi-index tool

This manuscript has been checked with the Fi-index tool and obtained a score of 0 on the date 15/01/23 according to Scopus® for the first, author only (references).

## Discussion

In this descriptive study, we compared our cohort population as cases with or without PH and their DMFT scores and their components to evaluate any association between DMFT scores and PH in the RCS. Filling teeth had a relation with EPH in both crude and adjusted models. DMFT, decayed, and missing teeth had no association with EPH or CPH in the crude and adjusted models.

Our study did not show a relationship between DMFT score or decayed teeth and PH, and the theory of the relationship between migraine and oral and dental disease was not approved directly in this research, as with the Nishihara and Peskersoy studies [9, 10]. However, from another point of view, filling teeth may be related to dental decay and could be a point of relation between decay and PH.

In Mattila et al.’s study, there was a relationship between DMFT and episodic headache in five-year-old children, similar to our results regarding the relationship of some parts of DMFT, such as filled teeth, with EPH. However, in Mattila’s study, the description of the headache was not clear, and whether it was a primary or secondary headache was not given [11].

In another study, Khatatbeh et al. found a caries relationship with headaches in children, but there was a difference between headaches in our study, which were PH with secondary headaches, as described in that study [12]. Additionally, there is another difference between these studies and ours in the population studied (children vs. adults in ours).

A Taiwan cohort study showed that oral and dental diseases increased the risk of migraine during the 13-year follow-up period [19], which was not supported by our study because in the present study, headaches included not only, migraine but also other PH, and it was a cross-sectional study. This relation may be hidden by more tension-type headache cases, which may not be related to inflammation in our study.

Leira et al. showed a relationship between chronic periodontitis (CP) and chronic migraine (CM) by high procalcitonin in the CP-CM group [20]. Ameijoria found that CM cases have more occurrences of CP than non-CM cases [21]. Another study also found a relationship between CM and CP in which some mechanisms for this relationship were described [22]. In a thesis performed by Bryn E Davis, there was no association between CP and headache disorders, similar to our finding. Instead, they found a relationship between *Porphyromonas gingivalis* and migraine occurrence [23]. On the other hand, our study did not show similar findings, especially in the CPH group.

Our unique finding was the relation of filling teeth with EPH in this research, which may point to another suspicious pathogenesis for primary headaches. Amalgam is a filling material that is used extensively in the restoration of tooth decay, in comparison to composite, which is another material that has many side effects on humans [13, 24]. Some studies have shown the safety of Amalgam and its noninferiority in comparison to other nonamalgam restorations [25].

In confirmation of some results of our study, a study by Peskersoy et al. found that the mean number of decayed, missing, or filled teeth (DMFT) showed significant differences between those with and without migraines [9].

There is a possibility of a coexistence relationship between oral bacteria and humans through the salivary nitrate pathway that could activate migraines [26]. Filling teeth could point to the presence of oral commensal bacteria and their effect on decayed teeth and this study could support our results [26].

The findings reported by Gonzalez and colleagues are the first contribution to headache etiology research suggesting oral microorganisms as the cause of headaches linked to nitric oxide, which has been implicated in the pathophysiology of many headache disorders, such as migraines, tension-type, and cluster headaches [27]. This study is in line with ours, as filling teeth could point to the base of oral infection and subsequent events on teeth.

Our study’s strength is that it is population-based with a large sample size, which increases the external validity of the results in this study and prepares extensive data collection for the exposure of interest (DMFT and its components) and potential confounders. An advanced dental cohort and clinical examination by experienced dentists is another point of our study strength.

One of our limitations was the findings of the present research work might have been influenced by different kinds of bias, such as imprecise responses about previous headaches or diagnosis of PH, and wrong recall of the subject’s PH information. Another limitation was the cross-sectional design of the study which cannot support any causal relationship. Also, no other study with sufficient information on the relationship between DMFT and headache prevalence in adults in Iran has been conducted so far, and thus we could not compare our results with any related findings. We offer another cohort follow-up study by checking new cases of PH and their types in our cohort population compared with the DMFT score, which also presents a causal relation.

## Conclusion

Our results show that one of the important factors related to EPH could be oral and dental problems, especially filled teeth. These results could indicate the relationship between dental-filling materials and headache, which should be investigated in future studies.

### Electronic supplementary material

Below is the link to the electronic supplementary material.

Supplementary Material 1

## Data Availability

The datasets used and/or analyzed during the current study are available from the corresponding author upon reasonable request.

## Abbreviations

CPH: Chronic primary headache
EPH: Episodic primary headache
PH: Primary Headaches
BMI: Body mass index
DMFT: Decayed, missing and filled teeth
RCS: Rafsanjani cohort study
OHBRCS: Oral Health Branch of Rafsanjan Cohort Study
PERSIAN: Prospective epidemiological research studies in IrAN
TTH: Tension-type headache
TACs: Trigeminal autonomic cephalalgia
GBD: Global burden of disease
SES: Socioeconomic status
WSI: Wealth score index
CP: Chronic periodontitis
CM: Chronic migraine
QA: Quality assurance
QC: Quality control
