# Supplementary material for: Association between DMFT and primary headaches: a study based on the Rafsanjan cohort study
Source: BMC Oral Health. 2024 Jan 4;24:21. doi: 10.1186/s12903-023-03815-0 (PMC10768315; doi:10.1186/s12903-023-03815-0)
Supplement: Supplementary file 1 — Supplementary Material 1 [file 12903_2023_3815_MOESM1_ESM.pdf]

PERSIAN Cohort Study

Data Dictionary for Baseline Variables

PERSIAN Cohort Study

Data Dictionary for Baseline Variables

Table of Contents

Page

Introduction 5

General Questionnaire

- Personal Information 7
- Anthropometry 10
- Socioeconomic Information 11
- Fuel Use and Living Standards 13
- Life Style Exposures 14
  - Animal Contact 16
- Current Job Information 17
  - Job History 17
- Physical Activity 18
- Sleeping Habits 20
- Mobile Use 21
- Pesticide Use 22

Medical Questionnaire

- Past Medical History 25
- Sign and Symptoms 30
- Medication Use (Past and Present) 34
  - Selected Important Medications 34
- Family Medical History 35
  - Family Medical History (modified) 36

- Reproductive History (Women) 39
    - Contraception History 41
    - Hormonal Replacement Therapy 41
- Physical Examinations 42
    - Disability or Amputations 42
- Oral Health 43
- Personal Habits 44
    - Smoking History 45
    - Non-Cigarette Tobacco Use History 46
    - Drug Use History 47
    - Alcohol Use History 48
    - Dosage 49
- Blood Pressure 50

Nutrition Questionnaire

- Food Frequency Questionnaire (FFQ) 51
    - Local Foods in the FFQ 55
- Nutrients 59
- Dietary Habits 62
- Water Use 68
- Dietary Supplements 69

Biological Samples

- Biobank Samples 71
- Lab Test Results 72

Supplementary Tables and Figurs

- Figure S1 ……………..…………………………………………………………………………..…………………….. 75
- Province ID …………………………………………………………………………………………..…………..…..……. 76
- County ID …………………………………………………………………………………………..………..……..….. 77
- Medication ID …………………………………………………………………………………………...,…………..… 81

PERSIAN Cohort

Introduction

The PERSIAN Cohort is a prospective study aiming to include Iranians aged 35–70 years from 19 geographically distinct areas of Iran (Figure 1). While the Ministry of Health and Medical Education oversees the project, investigators at local universities carry it out. These include the Universities of Medical Sciences in Ahvaz, Ardabil, Bandar Abbas, Fasa, Guilan, Kermanshah, Kordestan, Mashhad, Mazandaran, Rafsanjan, Sabzevar, Shahrekord, Shiraz, Tabriz, Urmia, Yasuj, Yazd, and Zahedan.

PERSIAN was designed to represent all of the major ethnic groups living in different geographical areas of Iran (Figure 1), enabling it to capture a wide range of environmental diversity, lifestyle, and socioeconomic differences, as well as many other exposures influencing disease patterns that maybe very unique to one area or highly prevalent in another.

The PERSIAN Cohort baseline data collection consists of 32 questionnaires divided into 3 major parts: general, medical, and nutrition. Each part is administered by a trained interviewer and all study centers used the same devices and protocols for all data collection/measurements.

Variables from each questionnaire are listed in the corresponding tables in this document. Each table includes 6 columns as defined below:

1. V Code: the unique code for each variable
2. Variable Name: the name given to each variable in the database
3. Variable Type: the type of variable as follows:
    - **Categorical**: for qualitative variables with more than 2 categories
    - **Dichotomous**: for qualitative variables with 2 categories
    - **Discrete**: for quantitative variables with integer values
    - **Continuous**: for quantitative variables with decimal
    - **Characteristic**: for variables which contain characters
    - **Date**: for date variables including month/day/year
4. Description: a brief description of the variable
5. Coding/Unit: the coding label for categorical and dichotomous variables and measurement units for continuous variables
6. Comment: includes any pertinent information, if necessary.

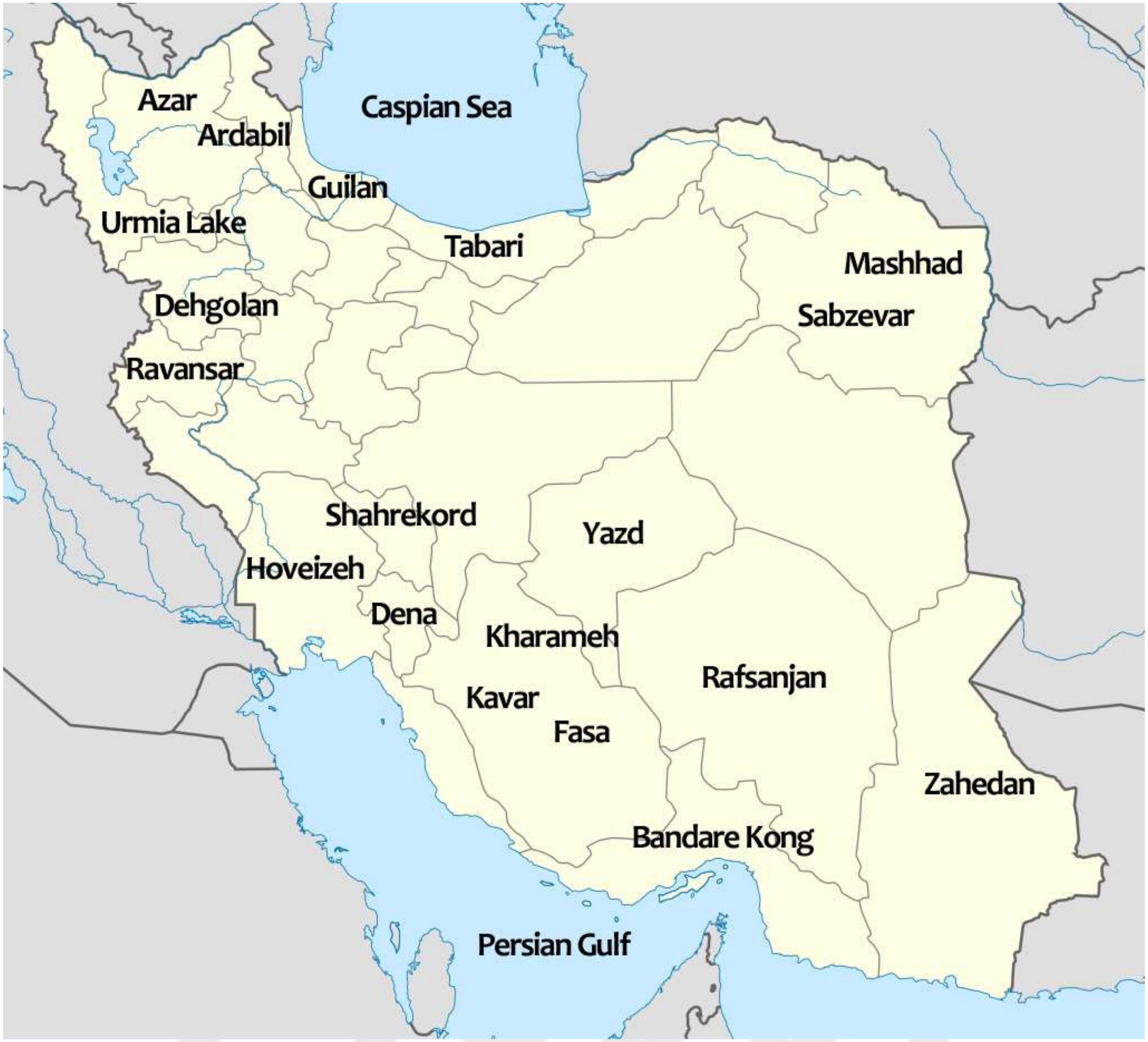

Figure 1. PERSIAN Cohort Sites

Table A: Personal Information					
**V Code**	**Variable Name**	**Variable Type**	**Description**	**Coding/Unit**	**Comment**
**A1**	**IRPC**	Discrete	The unique PERSIAN Cohort ID for each participant		
**A2**	**EnrollmentDate**	Date	Date of enrollment		
**A3**	**CohortCenterID**	Categorical	Unique code designated to each cohort center	2= Ravansar 12= Shahrekord<br>3= Guilan 13= Bandare Kong<br>4= Fasa 14= Urmia Lake<br>5= Azar 15= Ardabil<br>6= Kharameh 16= Sabzevar<br>7= Mazandaran 17= Mashhad<br>8= Zahedan 18= Dena<br>9= Yazd 19= Kavar<br>10= Rafsanjan 20= Dehgolan<br>11= Hoveizeh	
**A4**	**GenderID**	Dichotomous	Participant gender	1= Male, 2= Female	
**A5**	**BirthDate**	Date	Date of birth based on national ID card		
**A6**	**Age_At_Interview**	Continuous	Age on the date of interview	Year	Calculated using variables A2 and A5
**A7**	**BirthDateReal**	Date	The self reported date of birth		
**A8**	**MotherEthnicityID**	Categorical	Maternal ethnicity	1= Fars 9= Zaboli<br>2= Azari 10= Guilak<br>3= Balouch 11= Turk Nomad<br>4= Kurd 12= Arab Nomad<br>5= Lor 13= Mazani<br>6= Arab 14= Tut<br>7= Turkman 15= Other<br>8= Talesh 16= Turk	
**A9**	**MotherEthnicityOther**	Characteristic	Specifies ethnicity if "Other" is reported for variable A8		
**A10**	**FatherEthnicityID**	Categorical	Paternal ethnicity	1= Fars 9= Zaboli<br>2= Azari 10= Guilak<br>3= Balouch 11= Turk Nomad<br>4= Kurd 12= Arab Nomad<br>5= Lor 13= Mazani<br>6= Arab 14= Tut<br>7= Turkman 15= Other<br>8= Talesh 16= Turk	
**A11**	**FatherEthnicityOther**	Characteristic	Specifies ethnicity if "Other" is reported for variable A10		

**A12**	**BirthPlaceProvinceID**	Categorical	Province in which participant was born	Coding is presented in supplementary table S1: "Province ID"	
**A13**	**BirthPlaceCountyID**	Categorical	County in which participant was born	Coding is presented in supplementary table S2 "County ID"	
**A14**	**BirthPlaceCity**	Characteristic	City in which the participant was born		
**A15**	**BirthPlaceVillage**	Characteristic	Village in which participant was born		
**A16**	**ProvinceID**	Categorical	Province of residence	Coding is presented in supplementary table S1: "Province ID"	
**A17**	**CountyID**	Categorical	County of residence	Coding is presented in supplementary table S2 "County ID"	
**A18**	**City**	Characteristic	City of residence		
**A19**	**Village**	Characteristic	Village of residence		
**A20**	**EducationYears**	Discrete	The number of years the participant received education	Year	
**A21**	**LastEduID**	Categorical	The highest level of education the participant completed/ last degree obtained	1= Elementary School<br>2= Middle School<br>3= High School diploma<br>4= Associate degree<br>5= Bachelor's degree<br>6= Master's degree<br>7= PhD<br>8= Illiterate	
**A22**	**FamilyStatusID**	Categorical	The participant's status/relationship in the household (at the time of enrollment)	1= Father<br>2= Mother<br>3= Child<br>4= Grandfather/grandmother<br>5= Uncle/Aunt (father's side)<br>6= Uncle/aunt (mother's side)<br>7= Other	
**A23**	**FamilyStatusOther**	Characteristic	Specifies family status, if "Other" is recorded for variable A22		
**A24**	**MaritalStatusID**	Categorical	Marital status of the participant	1= Single (never married)<br>2= Married<br>3= Widowed<br>4= Divorced<br>5= Other	
**A25**	**MaritalStatusOther**	Characteristic	Specifies marital status, if "Other" is recorded for variable A24		
**A26**	**MarriageNo**	Discrete	The number of times the participant has been married		Variables A26-A28 only available when a category other than "Single (never married)" is recorded for variable A24.
**A27**	**FirstMarriageAge**	Discrete	Age at first marriage	Year	
**A28**	**FamilyMarriageID**	Categorical	Having a consanguineous marriage	1= Yes (with first cousin)	

				2= No<br>3= Yes (with second cousin)	
**A29**	**EthnicityID**	Categorical	The participant's ethnicity	1= Fars 9= Zaboli<br>2= Azari 10= Guilak<br>3= Balouch 11= Turk Nomad<br>4= Kurd 12= Arab Nomad<br>5= Lor 13= Mazani<br>6= Arab 14= Tut<br>7= Turkman 15= Mixed<br>8= Talesh 16= Turk	Created variable based on the following: If the ethnicity of both parents was the same (variables A8 and A10), that ethnicity was also taken for the individuals. If parents had different ethnicities, the individual was considered to have a "Mixed" ethnicity.
**A30**	**ResidenceType**	Dichotomous	Specifies residence in urban vs. rural areas	1= Urban, 2= Rural	

Table B: Anthropometry*					
**V Code**	**Variable Name**	**Variable Type**	**Description**	**Coding/Unit**	**Comment**
**B1**	**HeightCm**	Continuous	Height	Centimeter (cm)	
**B2**	**WeightKg**	Continuous	Weight	Kilogram (kg)	
**B3**	**WaistCircumference**	Continuous	Waist circumference	Centimeter	
**B4**	**HipCircumference**	Continuous	Hip circumference	Centimeter	
**B5**	**WristCircumference**	Continuous	Wrist circumference	Centimeter	
**B6**	**BMI**	Continuous	Body mass index	$Kg/(m^2)$	calculated using the formula below = Weight (kg)/ Height $(m)^2$

* All anthropometric measurements were taken using US National Institutes of Health protocols (https://www.cdc.gov/nchs/data/nhanes/nhanes_07_08/manual_an.pdf). Some anthropometric measurements were not measured for pregnant women and individuals with disabilities hindering them to stand upright.

PERSIAN Cohort

Table C: Socioeconomic Information					
**V Code**	**Variable Name**	**Variable Type**	**Description**	**Coding/Unit**	**Comment**
**C1**	**HousingStatusID**	Categorical	Status of the house the individual currently lives in	1= Owned<br>2= Leased or Rented<br>3= Company home (governmental or private company)<br>4= Relative's house (only if rent is not paid)	
**C2**	**HouseArea**	Continuous	The participant's home area	m^2	Excluding the following spaces: porch, garden/yard, garage, or any place where house animals are kept (i.e. barn)
**C3**	**Houseroom No**	Discrete	The number of bedrooms in the participant's current home		
**C4**	**Family No**	Discrete	The number of people living in the participant's current home		
**C5**	**AccessFreezer**	Dichotomous	Access to a freezer at home	0= No, 1= Yes	
**C6**	**AccessWashingMachine**	Dichotomous	Access to a washing machine at home	0= No, 1= Yes	
**C7**	**AccessDishWasher**	Dichotomous	Access to a dish washer at home	0= No, 1= Yes	
**C8**	**AccessComputer**	Dichotomous	Access to a computer at home	0= No, 1= Yes	
**C9**	**AccessInternet**	Dichotomous	Access to internet access at home	0= No, 1= Yes	
**C10**	**AccessMotorcycle**	Dichotomous	Access to a motorcycle in the household	0= No, 1= Yes	
**C11**	**AccessCar**	Dichotomous	Access to a car in the household	0= No, 1= Yes	
**C12**	**CarPriceID**	Categorical	Specifies the price of the car, if "Yes " is reported for variable C11	1= <20 million Tomans<br>2= 20-50 million Tomans<br>3= 50-100 million Tomans<br>4= >100 million Tomans	
**C13**	**AccessVacuumCleaner**	Dichotomous	Access to a vacuum cleaner at home	0= No, 1= Yes	
**C14**	**AccessColorTV**	Dichotomous	Access to a color TV at home	0= No, 1= Yes	
**C15**	**ColorTvTypeID**	Dichotomous	Specifies the type of Color TV, if "Yes " is reported for variable C14	1= Regular, 2= Plasma	
**C16**	**AccessBathroom**	Dichotomous	Access to a bathroom inside the house	0= No, 1= Yes	Pertinent to old houses in Iran, where the bathroom was built outside the house (in the yard)
**C17**	**OwnMobile**	Dichotomous	Owning a mobile/cell phone	0= No, 1= Yes	
**C18**	**OwnPC**	Dichotomous	Owning a desktop computer	0= No, 1= Yes	
**C19**	**OwnLaptop**	Dichotomous	Owning a laptop	0= No, 1= Yes	
**C20**	**OwnInternet**	Dichotomous	Owning an internet account	0= No, 1= Yes	
**C21**	**OwnCar**	Dichotomous	Owning a car	0= No, 1= Yes	

**C22**	**OwnCarPriceID**	Categorical	Specifies the price of the owned car, if "Yes " is reported for variable C21	1= <20 million Tomans<br>2= 20-50 million Tomans<br>3= 50-100 million Tomans<br>4= >100 million Tomans	
**C23**	**BookNoRead**	Discrete	The number of books the participant has read in the past year		Excluding school books, those required for a job, and religious scriptures
**C24**	**ForeignTravelNo**	Discrete	The total number of international trips the participant has taken during lifetime		
**C25**	**ForeignPilgrimageNo**	Discrete	The number of international pilgrimage trips the participant has taken		
**C26**	**ForeignNonPilgrimageNo**	Discrete	The number of international non-pilgrimage trips the participant has taken		
**C27**	**NationalTravelNo**	Discrete	The number of trips the participant has made within Iran in the past 10 years		A trip is defined as: both pilgrimage or non-pilgrimage trips, that are at least 100 kilometers away from participant’s home
**C28**	**WSI_by_center**	Continuous	Wealth score index (separately for each center)		For each center, WSI is separately estimated by multiple correspondence analysis (MCA) of the variables listed below: (Access to a freezer, access to a washing machine, access to a dish washer, access to a computer, access to internet, access to a motorcycle, access to a car (no access, access to a car with price of <50 million Tomans, and access to a car with price of >50 million Tomans), access to a vacuum cleaner, color Tv type (no color Tv or regular color Tv vs. Plasma color Tv), owning a mobile, owning a PC or laptop, international trips in lifetime (never, just pilgrimage, both pilgrimage or non-pilgrimage trips)
**C29**	**WSI_total**	Continuous	Wealth score index (for all centers pooled)		For all centers, WSI is estimated by multiple correspondence analysis (MCA) of the variables listed below: (Access to a freezer, access to a washing machine, access to a dish washer, access to a computer, access to internet, access to a motorcycle, access to a car (no access, access to a car with price of <50 million

					Tomans, and access to a car with price of >50 million Tomans), access to a vacuum cleaner, color Tv type (no color Tv or regular color Tv vs. Plasma color Tv), owning a mobile, owning a PC or laptop, international trips in lifetime (never, just pilgrimage, both pilgrimage or non-pilgrimage trips)

Table D: Fuel Use and Living Standards*					
V Code	Variable Name	Variable Type	Description	Coding/Unit	Comment
D1	**LivingFromAge**	Discrete	Age at the beginning of each entry	Year	
D2	**LivingToAge**	Discrete	Age at the end of each entry	Year	
D3	**ProvinceID**	Categorical	Province of residence	Coding is presented in supplementary table S1: "Province ID"	
D4	**CountyID**	Categorical	County of residence	Coding is presented in supplementary table S2 "County ID"	
D5	**Village**	Characteristic	Village of residence		
D6	**HouseTypeID**	Categorical	Specifies the participant's house structure material	1= Bricks and steel<br>2= Wood and bricks<br>3= Cement/concrete<br>4= Stone and chalk/charcoal<br>5= Clay and mud<br>6= Other	
D7	**CookingFuelTypeID**	Categorical	Specifies the type of fuel used for cooking	1= Oil/gasoline<br>2= Wood/firewood<br>3= Animal waste<br>4= Gas<br>5= Other<br>6= No fuel used for cooking	
D8	**HeatingFuelTypeID**	Categorical	Specifies the type of fuel used for heating	1= Oil/gasoline<br>2= Wood/firewood<br>3= Animal waste<br>4= Gas<br>5= Other<br>6= No fuel used for cooking	
D9	**HeatingSystemID**	Categorical	Specifies the heating system in the house	1= Electrical heater<br>2= Oil heater with a chimney<br>3= Fire place<br>4= Oil heater without chimney<br>5= Gas heater<br>6= Other<br>7= No heating system	
* The variables in this table (D1-D9) have been repeated for each place of residence reported. Only residence of greater than or equal to one year have been recorded.					

Table E: Lifestyle Exposures					
**V Code**	**Variable Name**	**Variable Type**	**Description**	**Coding/Unit**	**Comment**
**E1**	**WaterSourceID1**	Dichotomous	Specifies the primary drinking water source (#1)	1= Well water<br>2= River water<br>3= Spring water<br>4= Tap water<br>5= Mineral water<br>6= Water Tank<br>7= Underground water Cistern<br>8= Other	Up to two sources, that are used more often than other sources, could have been reported.
**E2**	**WaterSourceID2**	Dichotomous	Specifies the primary drinking water source (#2)	1= Well water<br>2= River water<br>3= Spring water<br>4= Tap water<br>5= Mineral water<br>6= Water Tank<br>7= Underground water Cistern<br>8= Other	
**E3**	**TapWaterAllTime**	Dichotomous	Whether or not tap water has been used throughout lifetime	0= No, 1= Yes	This variable is only completed if "Tap water" is selected as a primary water sources in variables E1-E2.<br><br>Variable significant for under-developed areas that may not have had tap water at some point in participant's lifetime
**E4**	**TapWaterUseYear**	Continuous	The number of years the participant has been using tap water	years	This variable is only completed if "No" is reported for variable E3
**E5**	**BeforeTapWaterSourceID1**	Dichotomous	Specifies the primary drinking water source prior to access to tap water (#1)	1= Well water<br>2= River water<br>3= Spring water<br>5= Mineral water<br>6= Water tank<br>7= Underground water cistern<br>8= Other	Variables E5-E6 are only completed if "No" is reported for variable E3.<br><br>Up to two sources, that are used more often than other sources, could have been reported.
**E6**	**BeforeTapWaterSourceID2**	Dichotomous	Specifies the primary drinking water source prior to access to tap water (#2)	1= Well water<br>2= River water<br>3= Spring water<br>5= Mineral water<br>6= Water tank<br>7= Underground water cistern<br>8= Other	

**E7**	**IsWaterSourceHealthy**	Dichotomous	Whether or not water sources specified in E5-E6 were approved by the government for use	0= No, 1= Yes	This variable is only completed if a water source other than "Tap water" is reported for variables E1-E2 or if "No" is reported for variable E3. Government approval would come from both the Ministry of Health and the Water/Waterwaste Department of Ministry of Energy
**E8**	**HasWaterPurifier**	Dichotomous	Use of a water filter (past or present)	0= No, 1= Yes	
**E9**	**KitchenTypeID**	Categorical	Specifies kitchen placement in the house	1= closed kitchen inside the house<br>2= open kitchen inside the house<br>3= outside of the house	
**E10**	**KitchenHoodUseID**	Categorical	Use of a kitchen hood or ventilator	1= Always<br>2= Sometimes<br>3= No/Doesn't have one	
**E11**	**HasKitchenWindow**	Dichotomous	Whether or not the kitchen has windows	0= No, 1= Yes	
**E12**	**CookingWinOpen**	Dichotomous	Whether or not the kitchen window is opened during cooking, if Yes" is reported for variable E11	0= No, 1= Yes	
**E13**	**HasAnimalContact**	Dichotomous	Having ever had contact with animals	0= No, 1= Yes	Contact is defined as touching or being in areas where animals are present.<br>If "Yes", table "Ea" is also completed.

Table Ea: Animal Contact *					
**V Code**	**Variable Name**	**Variable Type**	**Description**	**Coding/Unit**	**Comment**
**EA1**	**AnimalContactFromAge**	Discrete	Age at the beginning of each contact entry	Year	
**EA2**	**AnimalContactToAge**	Discrete	Age at the end of each contact entry	Year	
**EA3**	**AnimalContactLevelTypeID**	Categorical	Specifies the level of contact with animals	1= Sometimes (i.e. animals residing within 200 meters of home or workplace)<br>2= Not daily, but at least twice each month (i.e. animals residing next to home or workplace)<br>3= Daily (i.e. animals kept in the house or workplace, but participant is not involved in taking care of the animal)<br>4= Close daily contact (i.e. feeding or cleaning animal living area)	
**EA4**	**AnimalTypeID**	Categorical	Specifies the type of animal	1= Horses/ donkeys /camels<br>2= Sheep/cows/goats<br>3= Dogs<br>4= Birds<br>5= Cats<br>6= Other	
* The variables in this table (Ea1-Ea4) have been repeated for each different type of animal contact reported.					

PERSIAN Cohort

Table F: Current Job Information					
**V Code**	**Variable Name**	**Variable Type**	**Description**	**Coding/Unit**	**Comment**
**F1**	**HasJob_new**	Dichotomous	The participant is employed (at the time of enrollment visit)	0= No,<br>1= Yes,<br>2=Retired,<br>3=Housewife/husband	A job is defined as working at least 8 hours per week. Seasonal works are also recorded (i.e. farming)
**F2**	**IncomeSourceInsurance**	Dichotomous	The participant's primary source of income/financial support comes from: insurance / retirement	0= No, 1= Yes	
**F3**	**IncomeSourceSelf**	Dichotomous	The participant's primary source of income/financial support comes from: earned, self	0= No, 1= Yes	
**F4**	**IncomeSourceSpouse**	Dichotomous	The participant's primary source of income/financial support comes from: spouse	0= No, 1= Yes	
**F5**	**IncomeSourceChildren**	Dichotomous	The participant's primary source of income/financial support comes from: children	0= No, 1= Yes	
**F6**	**IncomeSourceWelfareCharity**	Dichotomous	The participant's primary source of income/financial support comes from: welfare/charity	0= No, 1= Yes	
**F7**	**IncomeSourceParents**	Dichotomous	The participant's primary source of income/financial support comes from: parents	0= No, 1= Yes	
**F8**	**IncomeSourceOther**	Dichotomous	The participant's primary source of income/financial support comes from: sources other than those stated in F2-F7	0= No, 1= Yes	
**F9**	**JobName**	Characteristic	Specifies the participant's job title		
**F10**	**PartnerJob**	Characteristic	If married, the job title of the participant's spouse		

Table Fa: Job History*					
**V Code**	**Variable Name**	**Variable Type**	**Description**	**Coding/Unit**	**Comment**
**Fa1**	**JobFromAge**	Discrete	Age at the beginning of each job entry	Year	
**Fa2**	**JobToAge**	Discrete	Age at the end of each job entry	Year	
**Fa3**	**JobName**	Characteristic	Job title		
* The variables in this table (Fa1-Fa4) have been repeated for each job reported. Only positions held for greater than or equal to one year have been recorded. A job is defined as working at least 8 hours per week. Seasonal works are also recorded (i.e. farming)					

Table G: Physical Activity					
**V Code**	**Variable Name**	**Variable Type**	**Description**	**Coding/Unit**	**Comment**
**G1**	**SleepDuration24h1**	Continuous	Night time sleep	Hour	MET= 0.9
**G2**	**SleepDurationMidDay1**	Continuous	Day time sleeping (naps)	Hour	MET= 0.9
**G3**	**Rest1**	Continuous	Resting/laying in bed awake	Hour	MET= 1.0
**G4**	**TV1**	Continuous	Watching TV/movies on laptop/PC, listening to music, etc.	Hour	MET= 1.0
**G5**	**Study1**	Continuous	Reading books, magazines, and newspapers	Hour	MET= 1.0
**G6**	**AtDeskWork1**	Continuous	Working while seated (i.e. administrative work)	Hour	MET= 1.5
**G7**	**Computer1**	Continuous	Working at a computer or desk	Hour	MET= 1.5
**G8**	**Eating1**	Continuous	Eating, sitting in a meeting, party or conference	Hour	MET= 1.5
**G9**	**Driving1**	Continuous	Driving (motorcycle or vehicle), operational work while seated	Hour	MET= 1.5
**G10**	**DrivingHeavyVehicle1**	Continuous	Driving agricultural or road construction machinery	Hour	MET= 2.0
**G11**	**Cooking1**	Continuous	Cooking, washing the dishes, and similar activities while standing	Hour	MET= 2.5
**G12**	**HouseCleaning1**	Continuous	House cleaning, washing clothes, sweeping floors and other light house work	Hour	MET= 3.0
**G13**	**Sale1**	Continuous	Vending on the street (if applicable)	Hour	MET= 3.0
**G14**	**Walking1**	Continuous	Walking, going down the stairs, slow dancing	Hour	MET= 3.5
**G15**	**LightTechnicalJobs1**	Continuous	Light technical services (i.e. oil change, car service, car wash, battery maintenance, etc.)	Hour	MET= 4.0
**G16**	**Masonary1**	Continuous	Light building construction activities (eg. painting, plastering), carpentry	Hour	MET= 4.0
**G17**	**LightAgricultural1**	Continuous	Gardening or light agricultural activities	Hour	MET= 4.0
**G18**	**AerobicExercise1**	Continuous	Brisk walking, light aerobic exercise, bicycling to work or for pleasure	Hour	MET= 4.5
**G19**	**CarryLightObject1**	Continuous	Carrying furniture, carrying light objects up stairs	Hour	MET= 5.0
**G20**	**HeavyEngineeringJobs1**	Continuous	Heavy mechanical services (such as motor vehicle services, blacksmithing, turning, casting, and etc.) or working in wood carving workshops	Hour	MET= 6.5
**G21**	**HeavyLaborAgricultJobs1**	Continuous	Heavy labor or agricultural activities (combined with activities such as shoveling, sawing, knocking, hammering), shoveling snow, or carrying heavy objects up stairs	Hour	MET= 6.5

**G22**	**HeavyExercise1**	Continuous	Professional sport activities such as running, bicycling, racing or heavy aerobic exercise	Hour	MET= 8.0
**G23**	**Duration1**	Discrete	Specifies the number of months in a year that the participant follows all the above activities (G1-G22)	Month	If more than 1 pattern of physical activity is reported (i.e. for seasonal works), up to two patterns including the most number of months in a year are recorded. The variable names for the second pattern are the same as above, ending with the number 2.
**G24**	**MET**	Continuous	Metabolic equivalent of task (MET) calculated for 24 hours	Hour per day	Calculated using the METs specified for each activity (above in this column). If two patterns were reported, the weighted mean of the corresponding METs were used to calculate the total MET score.

Questionnaire Notes:

- The total hours reported for activities in G1-G22 must equal 24 hours.
- Participant must spend at least 10 minutes performing an activity, in order for it to be recorded. The time spent on any regular activity not performed on a daily basis, (performed weekly or monthly), is converted to daily, and if it is still more than 10 minutes, it is recorded.
- If two activities are performed simultaneously, the activity with a higher MET is recorded, (i.e. if listening to music while doing dishes, the time spent is categorized as doing dishes).

Table H: Sleeping Habits					
**V Code**	**Variable Name**	**Variable Type**	**Description**	**Coding/Unit**	**Comment**
**H1**	NightSleepHour	Discrete	The time at which the participant usually falls asleep (hour)	Hour	
**H2**	NightSleepMin	Discrete	The time at which the participant usually falls asleep (minute)	Minute	
**H3**	FallingSleepDurationMin	Discrete	The length of time participant lays in bed before actually falling asleep	Minute	
**H4**	MorningWakeupHour	Discrete	The time at which the participant usually wakes up in the mornings (hour)	Hour	
**H5**	MorningWakeupMin	Discrete	The time at which the participant usually wakes up in the mornings (minute)	Minute	
**H6**	PreferredWakeupHour	Discrete	The time at which the participant would like to be able to wake up in the mornings (hour)	Hour	
**H7**	PreferredWakeupMin	Discrete	The time at which the participant would like to be able to wake up in the mornings (minute)	Minute	
**H8**	DayTimeNap	Dichotomous	Taking daily naps on a regular basis	0= No, 1= Yes	Regular is defined as greater than or equal to three time per week
**H9**	NapDurtionMin	Discrete	The duration of nap, if "Yes" is reported for variable H8	Minute	
**H10**	NightShiftWork	Dichotomous	Working night shifts in the year prior to the enrollment date	0= No, 1= Yes	Night shift is defined as at least 6 hours of work between 9PM and 6 AM
**H11**	NightShiftNoPerYear	Discrete	The number of night shift in the year prior to the enrollment date, if "Yes" is reported for variable H10		
**H12**	LegRestlessnessID	Categorical	Having leg restlessness while asleep	1= Yes<br>2= No<br>3= I don't know	
**H13**	DozingOff	Dichotomous	Dozing off during the day	0= No, 1= Yes	
**H14**	SleepingPillsUse	Dichotomous	Using sleeping pills on a regular basis	0= No, 1= Yes	Regular is defined as more than 2 times per week
**H15**	SleepDuration	Continuous	The time between sleep and wakeup hour	Hour	Calculated using H1, H2 and H4, H5

PERSIAN Cohort

**Table I: Mobile Use**					
**V Code**	**Variable Name**	**Variable Type**	**Description**	**Coding/Unit**	**Comment**
**I1**	**MobileUse**	Dichotomous	Whether or not the participant uses a mobile/cell phone	0=No, 1=Yes	Variables I2-I10 are only available if "Yes" is recorded for variable I1.
**I2**	**MobileUseDuration**	Continuous	The number of years the participant has used a mobile/cell phone	Year	
**I3**	**AnswerFrom(H/D)**	Continuous	Specifies the minimum time spent	Hour per day	Variables I3, I4 are linked and specify the amount of time participant spends using a phone for talking or answering phone calls in the 12 months prior to enrollment.
**I4**	**AnswerTo(H/D)**	Continuous	Specifies the maximum time spent	Hour per day	
**I5**	**UseFrom(H/D)**	Continuous	Specifies the minimum time spent	Hour per day	Variables I5, I6 are linked and specify the amount of time participant spends using a phone/tablet for doing things other than calling or answering calls (i.e for texting, chat, playing, or using the internet)
**I6**	**UseTo(H/D)**	Continuous	Specifies the maximum time spent	Hour per day	

PERSIAN Cohort

**Table J: Pesticide Use***					
**V Code**	**Variable Name**	**Variable Type**	**Description**	**Coding/Unit**	**Comment**
**J1**	**NearFarm**	Dichotomous	Whether or not the participant's house is in close proximity of farming areas	0=No, 1=Yes	
**J2**	**FarmDistanceID**	Categorical	Specifies distance, if "Yes" is reported for variable J1	1= Distant (more than 200 meters)<br>2= Quite close (100-200 meters)<br>3= Close (50-100 meters)<br>4= Very close (less than 50 meters)	
**J3**	**UseInFarm**	Dichotomous	Applying pesticides in farms, green houses, or agricultural fields	0=No, 1=Yes	
**J4**	**UseInFarmNo**	Discrete	The number of times pesticides were applied in farms, green houses or agricultural fields		Variables J4-J6 are only available if "Yes" is reported for variable J3
**J5**	**UseInFarmDuration**	Continuous	Average length of time spent applying pesticides in farms, green houses or agricultural fields	Minute	
**J6**	**UseInFarmPPE**	Dichotomous	Using PPEs when applying pesticides in farms, green houses or agricultural fields	0=No, 1=Yes	
**J7**	**UseInYard**	Dichotomous	Applying pesticides at home for plants	0=No, 1=Yes	
**J8**	**UseInYardNo**	Discrete	The number of times pesticides were applied at home for plants		Variables J8-J10 are only available if "Yes" is reported for variable J7
**J9**	**UseInYardDuration**	Continuous	Average length of time spent applying pesticides at home for plants	Minute	
**J10**	**UseInYardPPE**	Dichotomous	Using PPEs when applying pesticides at home for plants	0=No, 1=Yes	
**J11**	**UseInHome**	Dichotomous	Applying insecticides at home	0=No, 1=Yes	To kill mosquitoes, flies, ants, beetles, ...
**J12**	**UseInHomeNo**	Discrete	The number of times insecticides were applied at home		Variables J12-J14 are only available if "Yes" is reported for variable J11
**J13**	**UseInHomeDuration**	Continuous	Average length of time spent applying insecticide at home	Minute	
**J14**	**UseInHomePPE**	Dichotomous	Using PPEs when applying insecticides at home	0=No, 1=Yes	
**J15**	**HasPesticidePreparation**	Dichotomous	Mixing/loading pesticides or insecticides	0=No, 1=Yes	
**J16**	**PesticidePreparationNo**	Discrete	The number of times pesticides or insecticides were mixed/loaded		Variables J16-J18 are only available if

**J17**	**PesticidePreparationDuration**	Continuous	Average length of time spent mixing/loading pesticides or insecticides	Minute	"Yes" is reported for variable J15
**J18**	**PesticidePreparationPPE**	Dichotomous	Using PPEs when mixing/loading pesticides or insecticides	0=No, 1=Yes	
**J19**	**InPesticidePlace**	Dichotomous	Entering work areas where pesticides were just applied	0=No, 1=Yes	
**J20**	**InPesticidePlaceNo**	Discrete	The number of times entered work areas where pesticides were just applied		Variables J20-J22 are only available if "Yes" is reported for variable J19
**J21**	**InPesticidePlaceDuration**	Continuous	Average length of time spent in work areas where pesticides were just applied	Minute	
**J22**	**InPesticidePlacePPE**	Dichotomous	Using PPEs when entering work areas where pesticides were just applied	0=No, 1=Yes	
**J23**	**CleanPesticideEquipment**	Dichotomous	Cleaning equipment contaminated with pesticides	0=No, 1=Yes	Spray equipment, pesticide containers, storage areas
**J24**	**CleanPesticideEquipmentNo**	Discrete	The number of times equipment contaminated with pesticides were cleaned		Variables J24-J26 are only available if "Yes" is reported for variable J23
**J25**	**CleanPesticideEquipmentDuration**	Continuous	Average length of time spent cleaning equipment contaminated with pesticides	Minute	
**J26**	**CleanPesticideEquipmentPPE**	Dichotomous	Using PPEs when cleaning equipment contaminated with pesticides	0=No, 1=Yes	
**J27**	**FixPesticideEquipment**	Dichotomous	Repairing/using equipment that had recently been used to apply pesticides	0=No, 1=Yes	
**J28**	**FixPesticideEquipmentNo**	Discrete	The number of times equipment that had recently been used to apply pesticides, was repaired/used		Variables J28-J30 are only available if "Yes" is reported for variable J27
**J29**	**FixPesticideEquipmentDuration**	Continuous	Average length of time spent repairing/using equipment that had recently been used to apply pesticides	Minute	
**J30**	**FixPesticideEquipmentPPE**	Dichotomous	Using PPEs when repairing/using equipment that had recently been used to apply pesticides	0=No, 1=Yes	
**J31**	**storedPesticide**	Dichotomous	Handling stored pesticide products	0=No, 1=Yes	
**J32**	**storedPesticideNo**	Discrete	The number of times stored pesticide products were handled		Variables J32-J34 are only available if "Yes" is reported for variable J31
**J33**	**storedPesticideDuration**	Continuous	Average length of time spent handling stored pesticide products	Minute	

**J34**	**storedPesticidePPE**	Dichotomous	Using PPEs when handling stored pesticide products	0=No, 1=Yes	
**J35**	**ManageSprayingPesticide**	Dichotomous	Directing spray operations	0=No, 1=Yes	
**J36**	**ManageSprayingPesticideNo**	Discrete	The number of times spray operations were directed		Variables J36-J38 are only available if "Yes" is reported for variable J35
**J37**	**ManageSprayingPesticideDuration**	Continuous	Average length of time spent directing spray operations	Minute	
**J38**	**ManageSprayingPesticidePPE**	Dichotomous	Using PPEs when directing spray operations	0=No, 1=Yes	

***** Variable J3-J38 pertain to activities performed in the 12 months prior to the enrollment date.
PPE: Personal protective equipment

PERSIAN Cohort

**Table K: Past Medical History**					
**V Code**	**Variable Name**	**Variable Type**	**Description**	**Coding/Unit**	**Comment**
**K1**	**HasDiabetes**	Categorical	Diagnosed with diabetes	0= No, 1= Yes	
**K2**	**DiabetesStartAge**	Discrete	Age at the time of diabetes diagnosis	Year	
**K3**	**DiabetesInTreatment**	Dichotomous	Receiving treatment for diabetes	0= No, 1= Yes	
**K4**	**HasHypertension**	Categorical	Diagnosed with hypertension	0= No, 1= Yes	
**K5**	**HypertensionStartAge**	Discrete	Age at the time of hypertension diagnosis	Year	
**K6**	**HypertensionInTreatment**	Dichotomous	Receiving treatment for hypertension	0= No, 1= Yes	
**K7**	**HasCardiacDisease**	Dichotomous	Diagnosed with cardiac diseases	0= No, 1= Yes	Ischemic heart disease, heart failure
**K8**	**CardiacDiseaseStartAge**	Discrete	Age at the time of cardiac disease diagnosis	Year	
**K9**	**CardiacDiseaseInTreatment**	Dichotomous	Receiving treatment for cardiac disease	0= No, 1= Yes	
**K10**	**HasMI**	Dichotomous	Diagnosed with myocardial infarction (MI)	0= No, 1= Yes	
**K11**	**MIStartAge**	Discrete	Age at the time of MI	Year	
**K12**	**MIInTreatment**	Dichotomous	Received treatment for MI	0= No, 1= Yes	
**K13**	**HasStroke**	Dichotomous	Diagnosed with a stroke	0= No, 1= Yes	
**K14**	**StrokeStartAge**	Discrete	Age at the time of stroke	Year	
**K15**	**StrokeInTreatment**	Dichotomous	Received treatment for stroke	0= No, 1= Yes	
**K16**	**HasRenalFailure**	Dichotomous	Diagnosed with renal failure	0= No, 1= Yes	Including minor chronic kidney disease with only low GFR (not requiring dialysis), and major chronic kidney failure requiring dialysis or kidney transplant
**K17**	**RenalFailureStartAge**	Discrete	Age at the time of renal failure diagnosis	Year	
**K18**	**RenalFailureInTreatment**	Dichotomous	Receiving treatment for renal failure	0= No, 1= Yes	
**K19**	**HasFattyLiver**	Dichotomous	Diagnosed with fatty liver	0= No, 1= Yes	Diagnosed by a physician
**K20**	**FattyLiverStartAge**	Discrete	Age at the time of fatty liver diagnosis	Year	
**K21**	**FattyLiverInTreatment**	Dichotomous	Receiving treatment for fatty liver	0= No, 1= Yes	Non-pharmacological therapies such as diet and exercise are also considered as treatments
**K22**	**HasHepatitisB**	Dichotomous	Diagnosed with hepatitis B	0= No, 1= Yes	
**K23**	**HepatitisBStartAge**	Discrete	Age at the time of hepatitis B diagnosis	Year	
**K24**	**HepatitisBInTreatment**	Dichotomous	Receiving treatment for hepatitis B	0= No, 1= Yes	
**K25**	**HasHepatitisC**	Dichotomous	Diagnosed with hepatitis C	0= No, 1= Yes	
**K26**	**HepatitisCStartAge**	Discrete	Age at the time of hepatitis C diagnosis	Year	

**K27**	**HepatitisCInTreatment**	Dichotomous	Receiving treatment for hepatitis C	0= No, 1= Yes	
**K28**	**HasChronicLungDisease**	Dichotomous	Diagnosed with chronic lung disease	0= No, 1= Yes	Including tuberculosis, asthma, etc.
**K29**	**ChronicLungDiseaseStartAge**	Discrete	Age at the time of chronic lung disease diagnosis	Year	
**K30**	**ChronicLungDiseaseInTreatment**	Dichotomous	Receiving treatment for chronic lung disease	0= No, 1= Yes	
**K31**	**HasThyroidDisease**	Dichotomous	Diagnosed with thyroid disease	0= No, 1= Yes	Including hypothyroidism, hyperthyroidism, goiter and thyroid nodules
**K32**	**ThyroidDiseaseStartAge**	Discrete	Age at the time of thyroid disease diagnosis	Year	
**K33**	**ThyroidDiseaseInTreatment**	Dichotomous	Receiving treatment for thyroid disease	0= No, 1= Yes	
**K34**	**HasKidneyStone**	Dichotomous	Diagnosed with kidney stones	0= No, 1= Yes	
**K35**	**KidneyStoneStartAge**	Discrete	Age at the time of kidney stone diagnosis	Year	
**K36**	**KidneyStoneInTreatment**	Dichotomous	Received treatment for kidney stones	0= No, 1= Yes	
**K37**	**HasGallstone**	Dichotomous	Diagnosed with gallstones	0= No, 1= Yes	
**K38**	**GallstoneStartAge**	Discrete	Age at the time of gallstone diagnosis	Year	
**K39**	**GallstoneInTreatment**	Dichotomous	Received treatment for gallstones	0= No, 1= Yes	Regular followed-ups by a physician (without receiving medical treatment) is also considered to be a treatment
**K40**	**HasRheumaticDisease**	Dichotomous	Diagnosed with any kind of rheumatological disorder	0= No, 1= Yes	
**K41**	**RheumaticDiseaseStartAge**	Discrete	Age at the time of rheumatological disorder diagnosis	Year	
**K42**	**RheumaticDiseaseInTreatment**	Dichotomous	Receiving treatment for rheumatological disorder	0= No, 1= Yes	
**K43**	**HasSkinCancer**	Dichotomous	Diagnosed with skin cancer	0= No, 1= Yes	
**K44**	**SkinCancerStartAge**	Discrete	Age at the time of skin cancer diagnosis	Year	
**K45**	**SkinCancerInTreatment**	Dichotomous	Receiving treatment for skin cancer	0= No, 1= Yes	
**K46**	**HasBreastCancer**	Dichotomous	Diagnosed with breast cancer	0= No, 1= Yes	
**K47**	**BreastCancerStartAge**	Discrete	Age at the time of breast cancer diagnosis	Year	
**K48**	**BreastCancerInTreatment**	Dichotomous	Receiving treatment for breast cancer	0= No, 1= Yes	
**K49**	**HasStomachCancer**	Dichotomous	Diagnosed with stomach cancer	0= No, 1= Yes	
**K50**	**StomachCancerStartAge**	Discrete	Age at the time of stomach cancer diagnosis	Year	
**K51**	**StomachCancerInTreatment**	Dichotomous	Receiving treatment for stomach cancer	0= No, 1= Yes	
**K52**	**HasColorectalCancer**	Dichotomous	Diagnosed with colorectal cancer	0= No, 1= Yes	
**K53**	**ColorectalCancerStartAge**	Discrete	Age at the time of colorectal cancer diagnosis	Year	
**K54**	**ColorectalCancerInTreatment**	Dichotomous	Receiving treatment for colorectal cancer	0= No, 1= Yes	
**K55**	**HasBladderCancer**	Dichotomous	Diagnosed with bladder cancer	0= No, 1= Yes	

**K56**	**BladderCancerStartAge**	Discrete	Age at the time of bladder cancer diagnosis	Year	
**K57**	**BladderCancerInTreatment**	Dichotomous	Receiving treatment for bladder cancer	0= No, 1= Yes	
**K58**	**HasHSC**	Dichotomous	Diagnosed with hematopoietic system cancer (HSC)	0= No, 1= Yes	
**K59**	**HSCStartAge**	Discrete	Age at the time of HSC diagnosis	Year	
**K60**	**HSCInTreatment**	Dichotomous	Receiving treatment for HSC	0= No, 1= Yes	
**K61**	**HasEsophagealCancer**	Dichotomous	Diagnosed with esophageal cancer	0= No, 1= Yes	
**K62**	**EsophagealCancerStartAge**	Discrete	Age at the time of esophageal cancer diagnosis	Year	
**K63**	**EsophagealCancerInTreatment**	Dichotomous	Receiving treatment for esophageal cancer	0= No, 1= Yes	
**K64**	**HasProstateCancer**	Dichotomous	Diagnosed with prostate cancer	0= No, 1= Yes	
**K65**	**ProstateCancerStartAge**	Discrete	Age at the time of prostate cancer diagnosis	Year	
**K66**	**ProstateCancerInTreatment**	Dichotomous	Receiving treatment for prostate cancer	0= No, 1= Yes	
**K67**	**HasLungCancer**	Dichotomous	Diagnosed with lung cancer	0= No, 1= Yes	
**K68**	**LungCancerStartAge**	Discrete	Age at the time of lung cancer diagnosis	Year	
**K69**	**LungCancerInTreatment**	Dichotomous	Receiving treatment for lung cancer	0= No, 1= Yes	
**K70**	**HasBrainAndCNSCancer**	Dichotomous	Diagnosed with brain or CNS cancer	0= No, 1= Yes	
**K71**	**BrainAndCNSCancerStartAge**	Discrete	Age at the time of brain or CNS cancer diagnosis	Year	
**K72**	**BrainAndCNSCancerInTreatment**	Dichotomous	Receiving treatment for brain or CNS cancer	0= No, 1= Yes	
**K73**	**HasEpilepsy**	Dichotomous	Diagnosed with epilepsy	0= No, 1= Yes	
**K74**	**EpilepsyStartAge**	Discrete	Age at the time of epilepsy diagnosis	Year	
**K75**	**EpilepsyInTreatment**	Dichotomous	Receiving treatment for epilepsy	0= No, 1= Yes	
**K76**	**HasChronicHeadaches**	Dichotomous	Diagnosed with chronic recurrent headaches	0= No, 1= Yes	Chronic recurrent headaches are defined as headaches occurring at least 15 days in a month, continuing for at least three consecutive months. Migraine disease is considered to be a type of chronic recurrent headache.
**K77**	**ChronicHeadachesStartAge**	Discrete	Age at the time of diagnosis with chronic recurrent headaches	Year	
**K78**	**ChronicHeadachesInTreatment**	Dichotomous	Receiving treatment for chronic recurrent headaches	0= No, 1= Yes	
**K79**	**HasDepression**	Dichotomous	Diagnosed with depression	0= No, 1= Yes	Diagnosed by a physician
**K80**	**DepressionStartAge**	Discrete	Age at the time of depression diagnosis	Year	
**K81**	**DepressionInTreatment**	Dichotomous	Receiving treatment for depression	0= No, 1= Yes	
**K82**	**HasPsychiatricDisorder**	Dichotomous	Diagnosed with any kind of psychiatric disorder, other than depression	0= No, 1= Yes	Including obsessive compulsive disorder, anxiety

					disorders, schizophrenia, bipolar disorder, etc. Addiction is also considered to be a psychiatric disorder.
**K83**	**PsychiatricDisorderStartAge**	Discrete	Age at the time of diagnosis of any psychiatric disorder, other than depression	Year	
**K84**	**PsychiatricDisorderInTreatment**	Dichotomous	Receiving treatment for any psychiatric disorder, other than depression	0= No, 1= Yes	
**K85**	**HasLearningDisability**	Dichotomous	Diagnosed with any kind of learning disability that has prevented the participant from educational achievements	0= No, 1= Yes	In childhood or adulthood
**K86**	**LearningDisabilityStartAge**	Discrete	Age at the time of learning disability diagnosis	Year	
**K87**	**LearningDisabilityInTreatment**	Dichotomous	Receiving treatment for learning disability	0= No, 1= Yes	
**K88**	**HasAmnesia**	Categorical	Diagnosed with any kind of amnesia that has led to severe difficulty in daily functioning	0= No, 1= Yes	
**K89**	**AmnesiaStartAge**	Discrete	Age at the time of amnesia diagnosis	Year	
**K90**	**AmnesiaInTreatment**	Dichotomous	Receiving treatment for amnesia	0= No, 1= Yes	
**K91**	**HasLaryngealCancer**	Dichotomous	Diagnosed with laryngeal cancer	0= No, 1= Yes	
**K92**	**LaryngealCancerStartAge**	Discrete	Age at the time of laryngeal cancer diagnosis	Year	
**K93**	**LaryngealCancerInTreatment**	Dichotomous	Receiving treatment for laryngeal cancer	0= No, 1= Yes	
**K94**	**HasTongueCancer**	Dichotomous	Diagnosed with tongue cancer	0= No, 1= Yes	
**K95**	**TongueCancerStartAge**	Discrete	Age at the time of tongue cancer diagnosis	Year	
**K96**	**TongueCancerInTreatment**	Dichotomous	Receiving treatment for tongue cancer	0= No, 1= Yes	
**K97**	**HasUterineCancer**	Dichotomous	Diagnosed with uterine cancer	0= No, 1= Yes	
**K98**	**UterineCancerStartAge**	Discrete	Age at the time of uterine cancer diagnosis	Year	
**K99**	**UterineCanceIn Treatment**	Dichotomous	Receiving treatment for uterine cancer	0= No, 1= Yes	
**K100**	**HasOvarianCancer**	Dichotomous	Diagnosed with ovarian cancer	0= No, 1= Yes	
**K101**	**OvarianCancerStartAge**	Discrete	Age at the time of ovarian cancer diagnosis	Year	
**K102**	**OvarianCancerInTreatment**	Dichotomous	Receiving treatment for ovarian cancer	0= No, 1= Yes	
**K103**	**HasLupus**	Dichotomous	Diagnosed with lupus disease	0= No, 1= Yes	
**K104**	**LupusStartAge**	Discrete	Age at the time of lupus diagnosis	Year	
**K105**	**LupusInTreatment**	Dichotomous	Receiving treatment for lupus	0= No, 1= Yes	
**K106**	**HasMS**	Dichotomous	Diagnosed with multiple sclerosis (MS)	0= No, 1= Yes	
**K107**	**MSStartAge**	Discrete	Age at the time of MS diagnosis	Year	
**K108**	**MSInTreatment**	Dichotomous	Receiving treatment for MS	0= No, 1= Yes	
**K109**	**HasPregnancyHypertension**	Dichotomous	Diagnosed with gestational hypertension	0= No, 1= Yes	

**K110**	**PregnancyHypertensionStartAge**	Discrete	Age at the time of gestational hypertension diagnosis	Year	Variables K109-K114 are only available for women.<br><br>K109: Includes preeclampsia, eclampsia, etc.
**K111**	**PregnancyHypertensionInTreatment**	Dichotomous	Received treatment for gestational hypertension	0= No, 1= Yes	
**K112**	**HasPregnancyDiabetes**	Dichotomous	Diagnosed with gestational diabetes mellitus (GDM)	0= No, 1= Yes	
**K113**	**PregnancyDiabetesStartAge**	Discrete	Age at the time of diagnosis of GDM	Year	
**K114**	**PregnancyDiabetesInTreatment**	Dichotomous	Taking treatment for GDM	0= No, 1= Yes	
**K115**	**Description**	Characteristic	Any other comments		

Table L: Sign and Symptoms					
**V Code**	**Variable Name**	**Variable Type**	**Description**	**Coding/Unit**	**Comment**
**L1**	**HasSternumIrritation**	Dichotomous	Having ever experienced a burning sensation, pain, pressure or discomfort in the chest while walking fast, or walking uphill	0= No, 1= Yes	
**L2**	**HasSwelling**	Dichotomous	Having ever experienced swelling or edema in any body part, especially feet	0= No, 1= Yes	
**L3**	**HasUrineColorChanges**	Dichotomous	Having ever experienced any kind of urine discoloration	0= No, 1= Yes	
**L4**	**HasUrinaryProblems**	Dichotomous	Having ever experienced any of the following: 1) burning sensation while urinating 2) frequency of urination 3) nocturnal urination 4) bedwetting	0= No, 1= Yes	
**L5**	**HasAbnormalUrineTest**	Dichotomous	Having ever had an abnormal urine test	0= No, 1= Yes	Including a urinalysis positive for RBC, WBC, blood, etc. or positive urine culture.
**L6**	**HasHeartburn**	Dichotomous	Experiencing a burning sensation underneath the sternum (heart burn), during the past year	0= No, 1= Yes	
**L7**	**HeartburnFrequencyOfOccurrenceID**	Categorical	Specifies the frequency, if "Yes" is reported for variable L6	1= Almost daily<br>2= Several times each week<br>3= Several times each month<br>4= Every once in a while	
**L8**	**HasFoodRegurgitation**	Dichotomous	Experiencing food regurgitation, during the past year	0= No, 1= Yes	
**L9**	**FoodRegurgitationFrequencyOfOccurrenceID**	Categorical	Specifies the frequency, if "Yes" is reported for variable L8	1= Almost daily<br>2= Several times each week<br>3= Several times each month<br>4= Every once in a while	
**L10**	**HasGERD**	Dichotomous	Diagnosed with gastroesophageal reflux disease (GERD)	0= No, 1= Yes	
**L11**	**HasBloating**	Dichotomous	Experiencing bloating and distension of the abdomen, especially after eating, during the past year	0= No, 1= Yes	
**L12**	**BloatingFrequencyOfOccurrenceID**	Categorical	Specifies the frequency of bloating and distension of the abdomen, if "Yes" is reported for variable L11	1= Almost daily<br>2= Several times each week<br>3= Several times each month<br>4= Every once in a while	
**L13**	**BowelMovementInterval**	Discrete	The number of times the participant moves her/his bowels, per the time frame specified in variable L14		Variables L13-L14 are linked and specify the number of times the participant moves his/her bowels in a given time frame
**L14**	**BowelMovementIntervalTypeID**	Categorical	Specifies the time frame for variable L13	1= Daily<br>2= Weekly<br>3= Monthly	

**L15**	**HasBloodInStool**	Dichotomous	Having ever had fresh blood in stool	0= No, 1= Yes	
**L16**	**HasWeightLoss**	Dichotomous	Having ever had unintentional weight loss	0= No, 1= Yes	More than 5kg in one month, and without dieting/exercising
**L17**	**HasJaundice**	Dichotomous	Having ever had jaundice (skin or eyes)	0= No, 1= Yes	
**L18**	**JaundiceAge**	Discrete	Specifies the age, if "Yes" is reported for variable L17		Variables L18-L19 are linked and specify the age at jaundice at given unit
**L19**	**JaundiceAgeTypeID**	Categorical	Specifies the unit for variable L18	1= Week<br>2= Month<br>3= Year	
**L20**	**HasCough**	Dichotomous	Experiencing a cough, lasting for at least two weeks, during the past year	0= No, 1= Yes	
**L21**	**CoughTypeID**	Dichotomous	Specifies the type of the cough, if "Yes" is reported for variable L20	1= With sputum<br>2= Without sputum	
**L22**	**HasShortnessOfBreath**	Dichotomous	Experiencing shortness of breath and wheezing, lasting for at least two weeks, during the past year	0= No, 1= Yes	
**L23**	**HasGaitProblem**	Dichotomous	Having ever experienced problems with gait (balance and coordination), lasting more than one week	0= No, 1= Yes	
**L24**	**HasFaint**	Dichotomous	Having experienced a short-term faint or syncopal episode, occurring for no specific reason, more than once in lifetime	0= No, 1= Yes	
**L25**	**HasThoughtDisorder**	Dichotomous	Having ever had difficulty/disruption in thought, memory or speaking, lasting more than one week	0= No, 1= Yes	
**L26**	**HasVisualImpairment**	Dichotomous	Having ever had an impaired vision, or double vision, lasting more than one week, but resolving on its own	0= No, 1= Yes	Including photophobia, color disruption etc.<br>Refractive errors are not considered in this question.
**L27**	**HasMuscleWeakness**	Dichotomous	Having ever experienced muscle weakness in any part of body, lasting more than one week	0= No, 1= Yes	
**L28**	**HasMovementDisorder**	Dichotomous	Having ever experienced movement impairments (stiffness and slowness) or tremors in limbs, lasting more than one week	0= No, 1= Yes	
**L29**	**HasNumbness**	Dichotomous	Having ever experienced loss of sensation, or a tingling sensation in hands or feet, lasting more than one week	0= No, 1= Yes	
**L30**	**HasBlowToTheHead**	Dichotomous	Having ever had a blow to the head, resulting in loss of consciousness	0= No, 1= Yes	Even if the loss of consciousness lasted for a short period of time
**L31**	**HasRecurringHeadaches**	Dichotomous	Having experienced persistent headaches more than once in lifetime	0= No, 1= Yes	Persistent is defined as lasting more than 4 hours each time
**L32**	**HasDizziness**	Dichotomous	Having ever experienced dizziness that has greatly impacted daily functioning	0= No, 1= Yes	

**L33**	**HasTinnitus**	Dichotomous	Having ever experienced a continuous wheezing sound in the ear (tinnitus), lasting more than one week	0= No, 1= Yes	
**L34**	**HasFiveYearFracture**	Dichotomous	Fracturing a bone in the past 5 years	0= No, 1= Yes	
**L35**	**FractureSite**	Characteristic	Specifies the location of fracture, if "Yes" is reported for variable L34		
**L36**	**HasFractureEver**	Dichotomous	Have you ever had a bone fracture	0=No, 1-Yes	
**L37**	**LastFractureAge**	Discrete	Specifies age of last bone fracture if “Yes” is reported for variable L36	Year	
**L38**	**LastFractureForFalling**	Dichotomous	Whether or not the last bone fracture occurred as a result of falling	0= No, 1= Yes	
**L39**	**FallingNo**	Discrete	The number of times the participant has fallen in the past year		High-energy traumas such as accidents resulting in falling, or sport and exercise-induced falling are not considered
**L40**	**HasHipFemoralFracture**	Dichotomous	Having ever had a hip or femur fracture	0= No, 1= Yes	
**L41**	**HasOsteoporosis**	Dichotomous	Diagnosed with osteoporosis, or have been told by a physician that she/he is at risk for developing it	0= No, 1= Yes	
**L42**	**HasBackPain**	Dichotomous	Having ever had back pain that disrupts daily functioning, lasting more than one week	0= No, 1= Yes	
**L43**	**HasBackpainStiffness**	Dichotomous	Having ever experienced back pain, with morning stiffness of one hour or more	0= No, 1= Yes	
**L44**	**HasJointpain**	Dichotomous	Having ever had joint pain	0= No, 1= Yes	
**L45**	**HasJointpainStiffness**	Dichotomous	Experiencing ever had joint pain, with morning stiffness of one hour or more	0= No, 1= Yes	
**L46**	**HasMouthAphthous**	Dichotomous	Having ever had recurring oral aphthous	0= No, 1= Yes	Recurring oral aphthous is defined as lesions occurring at least 4 times a year
**L47**	**HasGenitalAphthous**	Dichotomous	Having ever had recurring genital aphthous	0= No, 1= Yes	Recurring genital aphthous is defined as lesions occurring at least 4 times a year
**L48**	**HasRheumatoidArthritis**	Dichotomous	Diagnosed with rheumatoid arthritis by a physician	0= No, 1= Yes	
**L49**	**HasSurgery**	Categorical	Having had any surgery	1= Yes<br>2= No<br>3= Doesn’t know	
**L50**	**SurgeryNo**	Discrete	Specifies the number of surgeries, if "Yes" is reported for variable L49		
**L51**	**HasHospitalization**	Categorical	Having had any hospitalizations	1= Yes<br>2= No<br>3= Doesn’t know	
**L52**	**HospitalizationNo**	Discrete	Specifies the number of hospitalizations, if "Yes" is reported for variable L51		

**L53**	**HasTransfusion**	Categorical	Having had any transfusions	1= Yes<br>2= No<br>3= Doesn’t know	
**L54**	**TransfusionNo**	Discrete	Specifies the number of transfusions, if "Yes" is reported for variable L53		
**L55**	**DrugAllergy**	Dichotomous	Having had any drug allergies	0= No, 1= Yes	
**L56**	**DrugAllergyDetail**	Characteristic	Specifies the drug name, if "Yes" is reported for variable L55		
**L57**	**Description**	Characteristic	Any related comments		

PERSIAN Cohort

**Table M: Medication Use (Past and Present) ***					
**V Code**	**Variable Name**	**Variable Type**	**Description**	**Coding/Unit**	**Comment**
**M1**	**MedicationID**	Categorical	Specifies the medication name	Coding is presented in supplementary table S3: "Medication ID"	
**M2**	**UseNo**	Continuous	Specifies the number of medicines taken per time frame in variable M3		Variables M2-M3 are linked and specify the number of medicines taken in a given time frame
**M3**	**MedicationIntervalUsedTypeID**	Categorical	Specifies the time frame of medicine use	1= Daily<br>2= Weekly<br>3= Monthly	
**M4**	**DurationOfUse**	Continuous	The length of time the medicine has been used	Months	
* The variables in this table (M1-M4) have been repeated for each medication reported.					

**Table Ma: Selected Important Medications**					
**V Code**	**Variable Name**	**Variable Type**	**Description**	**Coding/Unit**	**Comment**
**Ma1**	**Anti_HTN_Drug**	Dichotomous	Using anti-hypertensive medications	0= No, 1= Yes	
**Ma2**	**Anti_DM_Pills**	Dichotomous	Using oral medication for diabetes	0= No, 1= Yes	
**Ma3**	**Insulin**	Dichotomous	Using insulin for diabetes	0= No, 1= Yes	
**Ma4**	**Statins**	Dichotomous	Using statins	0= No, 1= Yes	
**Ma5**	**TG_Lowering_Drug**	Dichotomous	Using triglyceride lowering medications	0= No, 1= Yes	

Table N: Family Medical History*					
**V Code**	**Variable Name**	**Variable Type**	**Description**	**Coding/Unit**	**Comment**
**N1**	**DiseaseHxID**	Categorical	Specifies the disease name	1= Diabetes<br>2= Hypertension<br>3= Cardiac disease<br>4= MI<br>5= Stroke<br>6= Stomach cancer<br>7= Colorectal cancer<br>8= Breast cancer<br>9= Prostate cancer<br>10= Skin cancer<br>11= Bladder cancer<br>12= Hematopoietic system cancer<br>13= Esophageal cancer<br>14= Lung cancer<br>15= Brain and CNS cancer<br>16= Epilepsy<br>17= Psychiatric disorders<br>18= Chronic recurrent headache<br>19= Alzheimer's disease<br>20= Pelvic or femoral fracture<br>21= Laryngeal cancer<br>22= Tongue cancer<br>23= Uterine cancer<br>24= Ovary cancer<br>25= Lupus disease<br>26= Multiple Sclerosis (MS)	
**N2**	**HasDisease**	Categorical	Whether or not anyone in the family has the diseases mentioned in variable N1	0= No, 1= Yes	
**N3**	**FamilyRelationID**	Categorical	Specifies the family member's relationship with participant	1= Father<br>2= Mother<br>3= Brother<br>4= Sister<br>5= Half-brother<br>6= Half-sister<br>7= Son<br>8= Daughter<br>9= Grandparent<br>10= Spouse<br>11= Other second-degree family members (including Uncle, Aunt and Cousin)	

* Variable N2 is repeated for each of the diseases specified in N1. If variable N2 is "Yes" for any of the diseases, the corresponding family member relationship code (N3) is also specified. If more than one family member has the same disease, the disease is repeated in the database, for all family member relationship codes reported.

PERSIAN Cohort

Table Na: Family Medical History (modified)					
V Code	Variable Name	Variable Type	Description	Coding/Unit	Comment
Na1	FH1_Diabetes	Dichotomous	Whether or not any first degree relatives have diabetes	0= No, 1= Yes	
Na2	FH2_Diabetes	Dichotomous	Whether or not any second degree relatives have diabetes	0= No, 1= Yes	
Na3	FH1_Hypertension	Dichotomous	Whether or not any first degree relatives have hypertension	0= No, 1= Yes	
Na4	FH2_Hypertension	Dichotomous	Whether or not any second degree relatives have hypertension	0= No, 1= Yes	
Na5	FH1_CardiacDisease	Dichotomous	Whether or not any first degree relatives have cardiac disease	0= No, 1= Yes	
Na6	FH2_CardiacDisease	Dichotomous	Whether or not any second degree relatives have cardiac disease	0= No, 1= Yes	
Na7	FH1_MI	Dichotomous	Whether or not any first degree relatives have had an MI	0= No, 1= Yes	
Na8	FH2_MI	Dichotomous	Whether or not any second degree relatives have had an MI	0= No, 1= Yes	
Na9	FH1_Stroke	Dichotomous	Whether or not any first degree relatives have had a stroke	0= No, 1= Yes	
Na10	FH2_Stroke	Dichotomous	Whether or not any second degree relatives have had a stroke	0= No, 1= Yes	
Na11	FH1_StomachCancer	Dichotomous	Whether or not any first degree relatives have/have had stomach cancer	0= No, 1= Yes	
Na12	FH2_StomachCancer	Dichotomous	Whether or not any second degree relatives have/have had stomach cancer	0= No, 1= Yes	
Na13	FH1_ColorectalCancer	Dichotomous	Whether or not any first degree relatives have/have had colorectal cancer	0= No, 1= Yes	
Na14	FH2_ColorectalCancer	Dichotomous	Whether or not any second degree relatives have/have had colorectal cancer	0= No, 1= Yes	
Na15	FH1_BreastCancer	Dichotomous	Whether or not any first degree relatives have/have had breast cancer	0= No, 1= Yes	
Na16	FH2_BreastCancer	Dichotomous	Whether or not any second degree relatives have/have had breast cancer	0= No, 1= Yes	
Na17	FH1_ProstateCancer	Dichotomous	Whether or not any first degree relatives have/have had prostate cancer	0= No, 1= Yes	
Na18	FH2_ProstateCancer	Dichotomous	Whether or not any second degree relatives have/have had prostate cancer	0= No, 1= Yes	
Na19	FH1_SkinCancer	Dichotomous	Whether or not any first degree relatives have/have had skin cancer	0= No, 1= Yes	
Na20	FH2_SkinCancer	Dichotomous	Whether or not any second degree relatives have/have had skin cancer	0= No, 1= Yes	
Na21	FH1_BladderCancer	Dichotomous	Whether or not any first degree relatives have/have had bladder cancer	0= No, 1= Yes	

**Na22**	**FH2_BladderCancer**	Dichotomous	Whether or not any second degree relatives have/have had bladder cancer	0= No, 1= Yes	
**Na23**	**FH1_HematopoieticCancer**	Dichotomous	Whether or not any first degree relatives have/have had hematopoietic system cancer	0= No, 1= Yes	
**Na24**	**FH2_HematopoieticCancer**	Dichotomous	Whether or not any second degree relatives have/have had hematopoietic system cancer	0= No, 1= Yes	
**Na25**	**FH1_EsophagealCancer**	Dichotomous	Whether or not any first degree relatives have/have had esophageal cancer	0= No, 1= Yes	
**Na26**	**FH2_EsophagealCancer**	Dichotomous	Whether or not any second degree relatives have/have had esophageal cancer	0= No, 1= Yes	
**Na27**	**FH1_LungCancer**	Dichotomous	Whether or not any first degree relatives have/have had lung cancer	0= No, 1= Yes	
**Na28**	**FH2_LungCancer**	Dichotomous	Whether or not any second degree relatives have/have had lung cancer	0= No, 1= Yes	
**Na29**	**FH1_Brain_CNSCancer**	Dichotomous	Whether or not any first degree relatives have/have had brain and CNS cancer	0= No, 1= Yes	
**Na30**	**FH2_Brain_CNSCancer**	Dichotomous	Whether or not any second degree relatives have/have had brain and CNS cancer	0= No, 1= Yes	
**Na31**	**FH1_Epilepsy**	Dichotomous	Whether or not any first degree relatives have epilepsy	0= No, 1= Yes	
**Na32**	**FH2_Epilepsy**	Dichotomous	Whether or not any second degree relatives have epilepsy	0= No, 1= Yes	
**Na33**	**FH1_PsychiatricDisorders**	Dichotomous	Whether or not any first degree relatives have psychiatric disorders	0= No, 1= Yes	
**Na34**	**FH2_PsychiatricDisorders**	Dichotomous	Whether or not any second degree relatives have psychiatric disorders	0= No, 1= Yes	
**Na35**	**FH1_ChronicRecurrentHeadache**	Dichotomous	Whether or not any first degree relatives have chronic recurrent headaches	0= No, 1= Yes	
**Na36**	**FH2_ChronicRecurrentHeadache**	Dichotomous	Whether or not any second degree relatives have chronic recurrent headaches	0= No, 1= Yes	
**Na37**	**FH1_Alzheimer**	Dichotomous	Whether or not any first degree relatives have alzheimer’s disease	0= No, 1= Yes	
**Na38**	**FH2_Alzheimer**	Dichotomous	Whether or not any second degree relatives have alzheimer’s disease	0= No, 1= Yes	
**Na39**	**FH1_Pelvic_FemoralFracture**	Dichotomous	Whether or not any first degree relatives have had pelvic or femoral fracture	0= No, 1= Yes	
**Na40**	**FH2_Pelvic_FemoralFracture**	Dichotomous	Whether or not any second degree relatives have had pelvic or femoral fracture	0= No, 1= Yes	
**Na41**	**FH1_LaryngealCancer**	Dichotomous	Whether or not any first degree relatives have/have had laryngeal cancer	0= No, 1= Yes	
**Na42**	**FH2_LaryngealCancer**	Dichotomous	Whether or not any second degree relatives have/have had laryngeal cancer	0= No, 1= Yes	
**Na43**	**FH1_TongueCancer**	Dichotomous	Whether or not any first degree relatives have/have had tongue cancer	0= No, 1= Yes	

**Na44**	**FH2_TongueCancer**	Dichotomous	Whether or not any second degree relatives have/have had tongue cancer	0= No, 1= Yes	
**Na45**	**FH1_UterineCancer**	Dichotomous	Whether or not any first degree relatives have/have had uterine cancer	0= No, 1= Yes	
**Na46**	**FH2_UterineCancer**	Dichotomous	Whether or not any second degree relatives have/have had uterine cancer	0= No, 1= Yes	
**Na47**	**FH1_OvaryVancer**	Dichotomous	Whether or not any first degree relatives have/have had ovarian cancer	0= No, 1= Yes	
**Na48**	**FH2_OvaryVancer**	Dichotomous	Whether or not any second degree relatives have/have had ovarian cancer	0= No, 1= Yes	
**Na49**	**FH1_Lupus**	Dichotomous	Whether or not any first degree relatives have lupus	0= No, 1= Yes	
**Na50**	**FH2_Lupus**	Dichotomous	Whether or not any second degree relatives have lupus	0= No, 1= Yes	
**Na51**	**FH1_MS**	Dichotomous	Whether or not any first degree relatives have multiple sclerosis (MS)	0= No, 1= Yes	
**Na52**	**FH2_MS**	Dichotomous	Whether or not any second degree relatives have multiple sclerosis (MS)	0= No, 1= Yes	

Table O: Reproductive History (Women)*					
V Code	Variable Name	Variable Type	Description	Coding/Unit	Comment
**O1**	**HasMenstruation**	Categorical	Having ever had a menstrual cycle	0= No, 1= Yes	
**O2**	**MenstruationStartAge**	Discrete	Age at the time of first menstruation, if "Yes" is reported for variable O1	Year	
**O3**	**NowPregnant**	Categorical	Pregnancy status at the time of enrollment	1= Yes<br>2= No<br>3= Doesn't know	
**O4**	**PregnancyNo**	Discrete	Number of previous pregnancies		
**O5**	**FirstPregnancyAge**	Discrete	Age at the first pregnancy, if variable O4 ≥ 1	Year	
**O6**	**AliveChildbirthNo**	Discrete	Number of live deliveries, if variable O4 ≥ 1		Not the number of live children, but live deliveries. For-example, twins are considered as 1 live delivery.
**O7**	**FirstAliveChildbirthAge**	Discrete	Age at the first live delivery, if variable O6 ≥ 1	Year	
**O8**	**HasStillbirth**	Categorical	History of stillbirth, if variable O4 ≥ 1	0= No, 1= Yes	Stillbirth is defined as death of the pregnancy product after the 20th gestational week.
**O9**	**AbortionNo**	Discrete	Number of abortions, if variable O4 ≥ 1		Abortion is defined as death of the pregnancy product prior to the 20th gestational week.
**O10**	**FirstAbortionAge**	Discrete	Age at the time of first abortion, if variable O9 ≥ 1	Year	
**O11**	**BreastfeedingDuration**	Continuous	Total breastfeeding duration, if variable O4 ≥ 1	Month	
**O12**	**HasInfertility**	Dichotomous	History of primary infertility	0= No, 1= Yes	
**O13**	**UseInfertilityDrug**	Dichotomous	History of infertility treatment	0= No, 1= Yes	
**O14**	**OvaryRemovalTypeID**	Categorical	Having ever had an oophorectomy	1= Yes (unilateral)<br>2= Yes (bilateral)<br>3= No	
**O15**	**OvaryRemovalAge**	Discrete	Age at the time of oophorectomy, if "Yes" is reported for variable O14	Year	
**O16**	**HasTubectomy**	Dichotomous	Having had a tubectomy	0= No, 1= Yes	
**O17**	**HasHysterectomy**	Dichotomous	Having had a hysterectomy	0= No, 1= Yes	
**O18**	**HysterectomyAge**	Discrete	Age at the time of hysterectomy, if "Yes" is reported for variable O17	Year	

PERSIAN Cohort

**O19**	**UseContraceptiveDrug**	Dichotomous	Having ever used contraceptives including oral contraceptive pills (OCP), medroxyprogesterone acetate, cyclofem etc.	0= No, 1= Yes	Only hormonal contraceptive medications included.
**O20**	**HasMenopause**	Dichotomous	Reaching menopause	0= No, 1= Yes	Menopause is defined as passage of at least 12 months since the last menstruation
**O21**	**MenopauseAge**	Discrete	Age at menopause, if "Yes" is reported for variable O20	Year	
**O22**	**NormalMenopause**	Discrete	Whether or not menopause was reached naturally, if "Yes" is reported for variable O20	0= No, 1= Yes	Induced menopause (surgery, chemotherpay) are not considered natural
**O23**	**UseHormonalReplacementDrug**	Categorical	Having ever used hormonal replacement therapy (HRT) such as Estrogen or Progesterone	1= Yes<br>2= No<br>3= Doesn't know	All kinds of HRT are considered, even topical vaginal ointments
**O24**	**HasCervicalOrBreastScreening**	Categorical	Having ever had breast or cervical cancer screening	1= Yes<br>2= No<br>3= Doesn't know	
**O25**	**HasBreastExam**	Dichotomous	Having ever had breast examinations performed by a midwife/physician, if "Yes" is reported for variable O24	0= No, 1= Yes	
**O26**	**LastBreastExamAge**	Discrete	Age at the last breast examination, if "Yes" is reported for variable O25	Year	
**O27**	**BreastExamNo**	Discrete	The number of previous breast examinations, if "Yes" is reported for variable O25		
**O28**	**HasMammography**	Dichotomous	Having ever had a mammography, if "Yes" is reported for variable O24	0= No, 1= Yes	
**O29**	**LastMammographyAge**	Discrete	Age at the time of last mammography, if "Yes" is reported for variable O28	Year	
**O30**	**MammographyNo**	Discrete	The number of previous mammographies, if "Yes" is reported for variable O28		
**O31**	**HasPapSmear**	Dichotomous	Having ever had a pap smear, if "Yes" is reported for variable O24	0= No, 1= Yes	
**O32**	**LastPapSmearAge**	Discrete	Age at the time of last pap smear, if "Yes" is reported for variable O31	Year	
**O33**	**PapSmearNo**	Discrete	The number of previous pap smears, if "Yes" is reported for variable O31		
**O34**	**Description**	Characteristic	Any related comments		

* This questionnaire was only completed for women. Variables O3-O13 were not asked from single (never married) women.

Table Oa: Contraception History*					
V Code	Variable Name	Variable Type	Description	Coding/Unit	Comment
**Oa1**	**ContraceptionFromAge**	Discrete	Age at the beginning of each entry	Year	
**Oa2**	**ContraceptionToAge**	Discrete	Age at the end of each entry	Year	
**Oa3**	**ContraceptionTypeID**	Categorical	Specifies the type of contraception used	1= OCP<br>2= Implants<br>3= Long-term progesterone injection<br>4= Intrauterine devices (IUD)	
**Oa4**	**DurationMonth**	Discrete	The number of months the participant has used contraception	Month	
* The variables in this table (Oa1-Oa4) have been repeated for each type of contraception reported.<br>This table was not completed for single (never married) women.					

Table Ob: Hormonal Replacement Therapy*					
V Code	Variable Name	Variable Type	Description	Coding/Unit	Comment
**Ob1**	**HormoneFromAge**	Discrete	Age at the beginning of each entry	Year	
**Ob2**	**HormoneToAge**	Discrete	Age at the end of each entry	Year	
**Ob3**	**HormoneName**	Characteristic	Specifies the name of the hormonal medication		
**Ob4**	**DurationMonth**	Discrete	The number of months the participant has taken the hormonal medication	Month	
* The variables in this table (Ob1-Ob4) have been repeated for each hormonal replacement therapy reported.					

Table P: Physical Examinations					
**V Code**	**Variable Name**	**Variable Type**	**Description**	**Coding/Unit**	**Comment**
**P1**	**HasHairLoss**	Dichotomous	Experiencing hair loss	0= No, 1= Yes	
**P2**	**HairLossTypeID**	Categorical	Specifies the stage of hair loss, if "Yes" is reported for variable P1	1= Stage 2<br>2= Stage 2A<br>3= Stage 3<br>4= Stage 3A<br>5= Stage 3V<br>6= Stage 4<br>7= Stage 4A<br>8= Stage 5<br>9= Stage 5A<br>10= Stage 5V<br>11= Stage 6<br>12= Stage 7<br>13= Stage 1<br>14= Stage 2<br>15= Stage 3<br>16= Stage 4<br>17= Stage 5	Codes 1-12 and 13-17 represent different stages of hair loss in men and women, respectively (Figure S1)
**P3**	**FacialHirsutism**	Dichotomous	Having facial hirsutism	0= No, 1= Yes	Only asked in women
**P4**	**IrisColorID**	Categorical	Specifies the Iris Color	1= Brown<br>2= Hazel<br>3= Green<br>4= Blue or Grey	
**P5**	**HasAmputationOrDisability**	Dichotomous	Having any physical or sensational disabilities or any amputations	0= No, 1= Yes	Data from Table "Pa" is available only if "Yes" is reported for variable P5
**P6**	**HasSpineDisorder**	Dichotomous	Having spinal disorder	0= No, 1= Yes	
**P7**	**SpineDisorderTypeID**	Categorical	Specifies the type of spinal disorder, if "Yes" is reported for variable P6	1= Scoliosis<br>2= Lordosis<br>3= Kyphosis	
**P8**	**Description**	Characteristic	Any related comments		

Table Pa: Disability or Amputations*					
**V Code**	**Variable Name**	**Variable Type**	**Description**	**Coding/Unit**	**Comment**
**Pa1**	**DisabilityCauseID**	Categorical	Specifies the cause of disability	1= Congenital<br>2= War-related<br>3= Occupational/Accidental<br>4= Due to disease (diabetes)	
**Pa2**	**DisabilityTypeID**	Categorical	Specifies the disabled body part	1= Right hand<br>2= Left hand<br>3= Right leg<br>4= Left leg<br>5= Finger/s<br>6= Toe/s<br>7= Eye<br>8= Ear	
* The variables in this table (Pa1, Pa2) have been repeated for each disability reported.					

Table Q: Oral Health					
**V Code**	**Variable Name**	**Variable Type**	**Description**	**Coding/Unit**	**Comment**
**Q1**	**BrushingNoID**	Categorical	The number of times participant brushes teeth	1= Once daily<br>2= Twice daily<br>3= Three times a day<br>4= Others<br>5= Doesn’t brush<br>6= Has dentures	
**Q2**	**TeethNo**	Discrete	Total number of teeth	0-32	Only natural teeth are considered. Implants are not counted.
**Q3**	**DecayedTeethNo**	Discrete	The number of decayed teeth	0-32	If a tooth is both filled and decayed it is recorded as decayed
**Q4**	**MissingTeethNo**	Discrete	The number of teeth missing	0-32	This question only considers teeth pulled as a result of decaying. Congenitally missing teeth, and teeth absent for reasons other than decay (for instance trauma), are not counted in this category
**Q5**	**FilledTeeth**	Discrete	The number of teeth filled	0-32	
**Q6**	**HasOralLesion**	Dichotomous	Existence of any lesions inside the mouth	0= No, 1= Yes	
**Q7**	**UseFlossing**	Dichotomous	Whether or not the participant flosses teeth	0= No, 1= Yes	
**Q8**	**FlossingNo**	Discrete	Specified the frequency of flossing per week, if "Yes" is reported for variable Q7		
**Q9**	**HasDentures**	Dichotomous	Having dentures	0= No, 1= Yes	
**Q10**	**FromWhenDentures**	Discrete	Specifies the age at which the participant first used dentures, if "Yes" is reported for variable Q9	year	
**Q11**	**BrushingDenturesNoID**	Categorical	The number of times the participant brushes dentures, if "Yes" is reported for variable Q9	1= Once daily<br>2= Twice daily<br>3= Three times a day<br>4= Others<br>5= Doesn’t brush	
**Q12**	**UseMouthwash**	Dichotomous	Use of mouthwash	0= No, 1= Yes	
**Q13**	**MouthwashNo**	Discrete	Specifies the frequency of mouthwash use per week, , if "Yes" is reported for variable Q12		
**Q14**	**Description**	Characteristic	Any related comments		

Table R: Personal Habits					
**V Code**	**Variable Name**	**Variable Type**	**Description**	**Coding/Unit**	**Comment**
**R1**	**SmokeCigaretteTypeID**	Categorical	Having smoked at least 100 cigarettes during lifetime	1= Yes, 2= No	
**R2**	**FirstCigaretteAge**	Discrete	The age at which the participant smoked a cigarette for the first time	Year	Variables R2-R6, as well as Table "Ra" are available only if "Yes" are reported for variable R1.
**R3**	**RegularCigaretteAge**	Discrete	The age at which the participant started smoking regularly	Year	
**R4**	**CurrentSmokingTypeID**	Categorical	Whether or not the participant currently smokes	1= Yes, 3= No	
**R5**	**SmokingNo**	Discrete	The average number of times the participant smokes in 24 hours		
**R6**	**StopSmokingAge**	Discrete	The age at which the participant stopped smoking daily	Year	
**R7**	**SmokeInChildhood**	Dichotomous	Having a family member who smoked during participant's childhood	0= No, 1= Yes	
**R8**	**SmokeInHome**	Dichotomous	Whether or not the participant is/was exposed to smoke from a cigarette at home (passive/second-hand smoking)	0= No, 1= Yes	
**R9**	**SmokeInHomeNo**	Continuous	Specifies the hours per day, if "Yes" is reported for variable R8	hour	
**R10**	**SmokeInWorkplaceID**	Categorical	Whether or not the participant is exposed to smoke from a cigarette at work (passive/second-hand smoking)	1= Yes, 2= No	
**R11**	**SmokeInWorkplaceNo**	Continuous	Specifies the hours per day, if "Yes" is reported for variable R10	Hour	
**R12**	**UseNonCigTobacco**	Dichotomous	Tobacco use defined as using Naas, Hookah, Pipe, or Chopogh once per week for at least six months	0= No, 1= Yes	Data from Table "Rb" is available only if "Yes" is reported for variable R12.
**R13**	**UseDrugs**	Dichotomous	Illicit drugs use defined as using illicit drugs once per week for at least six months	0= No, 1= Yes	Data from Table "Rc" is available only if "Yes" is reported for variable R13.
**R14**	**UseAlcohol**	Dichotomous	Alcohol consumption defined as drinking approximately 200 ml of beer OR 45 ml of liquor, once per week for at least six months	0= No, 1= Yes	Data from Table "Rd" is available only if "Yes" is reported for variable R14.

Table Ra: Smoking History*					
V Code	Variable Name	Variable Type	Description	Coding/Unit	Comment
**Ra1**	**CigaretteFromAge**	Discrete	Age at the beginning of each entry	Year	
**Ra2**	**CigaretteToAge**	Discrete	Age at the end of each entry	Year	
**Ra3**	**CigaretteTypeID**	Categorical	Specifies the type of cigarette used	1= Manufactured cigarette<br>2= Hand-made cigarette<br>3= Cigar	
**Ra4**	**PerDay**	Discrete	Number of cigarettes the participant smoked in a day		
**Ra5**	**DayInDuration**	Discrete	Number of days the participant smoked per the time frame specified in Ra6		Variables Ra5-Ra6 are linked and specify the number of times the participant has smoked during a given time frame
**Ra6**	**DurationTypeID**	Categorical	Specifies the time frame for variable Ra5	1= Week<br>2= Month<br>3= Year	
* The variables in this table (Ra1-Ra6) have been repeated for each different time and pattern of smoking reported.					

PERSIAN Cohort

Table Rb: Non-Cigarette Tobacco Use History*					
**V Code**	**Variable Name**	**Variable Type**	**Description**	**Coding/Unit**	**Comment**
**Rb1**	**TobaccoFromAge**	Discrete	Age at the beginning of each entry	Year	
**Rb2**	**TobaccoToAge**	Discrete	Age at the end of each entry	Year	
**Rb3**	**TobaccoTypeID**	Categorical	Specifies the type of tobacco used	1=Naas<br>2=Chopogh<br>3= Hookah<br>4= Pipe	
**Rb4**	**PerDay**	Discrete	Number of times the participant smoked tobacco in a day		
**Rb5**	**DayInDuration**	Discrete	Number of days the participant smoked tobacco per the time frame specified in Rb6		Variables Rb5-Rb6 are linked and specify the number of times the participant has used tobacco during a given time frame
**Rb6**	**DurationTypeID**	Categorical	Specifies the time frame for variable Rb5	1=Week<br>2=Month<br>3=Year	
* The variables in this table (Rb1-Rb6) have been repeated for each different time and pattern of tobacco use reported.					

Table Rc: Drug Use History*					
V Code	Variable Name	Variable Type	Description	Coding/Unit	Comment
**Rc1**	**DrugFromAge**	Discrete	Age at the beginning of each entry	year	
**Rc2**	**DrugToAge**	Discrete	Age at the end of each entry	year	
**Rc3**	**DrugTypeID**	Categorical	Specifies the type of illicit drug used	1= Opium<br>2= Heroin<br>3= Sookhteh<br>4= Shireh<br>5= Methamphetamine<br>6= Cocaine<br>7= Crack<br>8= Crystal<br>9= Pan (pan parag)<br>10= Other	
**Rc4**	**DrugUseTypeID**	Categorical	Specifies the mode of use	1= Oral<br>2= Inhalation<br>3= Injection	
**Rc5**	**PerDay**	Discrete	Number of times the participant used illicit drug in a day		
**Rc6**	**DayInDuration**	Discrete	Number of days the participant used illicit drugs per the time frame specified in Rc7		Variables Rc6-Rc7 are linked and specify the number of times the participant has used illicit drugs during a given time frame
**Rc7**	**DurationTypeID**	Categorical	Specifies the time frame for variable Rc6	1= Week<br>2= Month<br>3= Year	

***** The variables in this table (Rc1-Rc7) have been repeated for each different time and pattern of illicit drug use reported.

PERSIAN Cohort

Table Rd: Alcohol Use History*					
**V Code**	**Variable Name**	**Variable Type**	**Description**	**Coding/Unit**	**Comment**
**Rd1**	**AlcoholFromAge**	Discrete	Age at the beginning of each entry	year	
**Rd2**	**AlcoholToAge**	Discrete	Age at the end of each entry	year	
**Rd3**	**AlcoholTypeID**	Categorical	Specifies the type of alcoholic drink used	1= Beer (5-7% alcohol)<br>2= Drinks with >40% alcohol (Vodka, Gin, Whisky, etc.)<br>3= Home-made drinks<br>4= Other	
**Rd4**	**PerDay**	Discrete	Number of times the participant used alcoholic drinks in a day		
**Rd5**	**DayInDuration**	Discrete	Number of days the participant used alcoholic drinks per the time frame specified in Rd6		Variables Rd5-Rd6 are linked and specify the number of times the participant has used alcohol during a given time frame
**Rd6**	**DurationTypeID**	Categorical	Specifies the time frame for variable Rd5	1= Week<br>2= Month<br>3= Year	

* The variables in this table (Rd1-Rd6) have been repeated for each different time and pattern of alcohol use reported.

Table Re: Dosage					
**V Code**	**Variable Name**	**Variable Type**	**Description**	**Coding/Unit**	**Comment**
**Re1**	**Pack_year**	Continuous	Specifies the dose of cigarette use throughout participant's life	Pack-year	Calculated using Table Ra
**Re2**	**Hookah_Dose**	Continuous	Specifies the dose of hookah use throughout participant's life	Dose-year (the number of years the participant used hookah once per day)	Calculated using Table Rb
**Re3**	**Other_Tob_Dose**	Continuous	Specifies the dose of tobacco (other than hookah) use throughout participant's life	Dose-year (the number of years the participant used tobacco once per day)	
**Re4**	**Opium_Dose**	Continuous	Specifies the dose of opium use throughout participant's life	Dose-year (the number of years the participant used opium once per day)	Calculated using Table Rc
**Re5**	**Other_Drug_Dose**	Continuous	Specifies the dose of drug use (other than opium) throughout participant's life	Dose-year (the number of years the participant used non-opium drugs once per day)	
**Re6**	**Beer_Dose**	Continuous	Specifies the dose of beer use throughout participant's life	Dose-year (the amount – milliliter- of beer participant used per day in a year)	Calculated using Table Rd
**Re7**	**Other_Alcohol_Dose**	Continuous	Specifies the dose of alcohol use (other than beer) throughout participant's life	Dose-year (the amount – milliliter- of alcoholic drinks (other than beer) participant used per day in a year)	

**Table S: Blood Pressure***					
**V Code**	**Variable Name**	**Variable Type**	**Description**	**Coding/Unit**	**Comment**
**S1**	**RightDBP1**	Discrete	Right arm first diastolic blood pressure	mmHg	For all measurements, the first measurement is taken in the sitting position, after at least 5 minutes of rest.<br><br>The second measurement is taken in the sitting position, at least 10 minutes after the first measurement.
**S2**	**RightDBP2**	Discrete	Right arm second diastolic blood pressure	mmHg	
**S3**	**RightSBP1**	Discrete	Right arm first systolic blood pressure	mmHg	
**S4**	**RightSBP2**	Discrete	Right arm second systolic blood pressure	mmHg	
**S5**	**LeftDBP1**	Discrete	Left arm first diastolic blood pressure	mmHg	
**S6**	**LeftDBP2**	Discrete	Left arm second diastolic blood pressure	mmHg	
**S7**	**LeftSBP1**	Discrete	Left arm first systolic blood pressure	mmHg	
**S8**	**LeftSBP2**	Discrete	Left arm second systolic blood pressure	mmHg	
**S9**	**PR1**	Discrete	First resting pulse rate	per minute	
**S10**	**PR2**	Discrete	Second resting pulse rate	per minute	
**S11**	**Description**	Characteristic	Any related comments		
**S12**	**SBP**	Continuous	The average of the second systolic blood pressure measurement in the right and left arms	mmHg	Calculated using S4 and S8
**S13**	**DBP**	Continuous	The average of the second diastolic blood pressure measurement in the right and left arms	mmHg	Calculated using S2 and S6

* Riester Exacta 1350 Sphygmomanometer was used to measure blood pressure in all PERSIAN Cohort centers. Multiple cuff sizes were available for use to best fit the participant’s arm.

PERSIAN Cohort

Table T: Food Frequency Questionnaire (FFQ)					
**V Code**	**Variable Name**	**Variable Type**	**Description**	**Coding/Unit**	**Comment**
**T1**	**1**	Continuous	Bread (Lavash)	Grams per day	
**T2**	**2**	Continuous	Bread (Barbari)	Grams per day	
**T3**	**3**	Continuous	Bread (Sangak)	Grams per day	
**T4**	**4**	Continuous	Baguette	Grams per day	
**T5**	**5**	Continuous	Cooked rice	Grams per day	
**T6**	**6**	Continuous	Cooked pasta, noodles	Grams per day	
**T7**	**7**	Continuous	Wheat, Oats, Barley	Grams per day	
**T8**	**171**	Continuous	Barley dread, diet dried bread	Grams per day	
**T9**	**10**	Continuous	Bean	Grams per day	
**T10**	**11**	Continuous	Chickpeas	Grams per day	
**T11**	**12**	Continuous	Mung beans, lentils, dhal	Grams per day	
**T12**	**13**	Continuous	Split peas	Grams per day	
**T13**	**14**	Continuous	Soybean	Grams per day	
**T14**	**15**	Continuous	Cooked fava beans/Lima beans	Grams per day	
**T15**	**16**	Continuous	Red meat (any kind)	Grams per day	
**T16**	**17**	Continuous	Chicken	Grams per day	
**T17**	**18**	Continuous	Chicken Giblets	Grams per day	
**T18**	**19**	Continuous	Eggs	Grams per day	
**T19**	**20**	Continuous	Fish	Grams per day	
**T20**	**21**	Continuous	Tuna (canned)	Grams per day	
**T21**	**22**	Continuous	Sausages, Kilbasa, Salami	Grams per day	
**T22**	**23**	Continuous	Hamburger	Grams per day	
**T23**	**24**	Continuous	Variety meats and by-products (heart, liver, kidney)	Grams per day	
**T24**	**25**	Continuous	Brain (sheep)	Grams per day	
**T25**	**26**	Continuous	Tongue (sheep)	Grams per day	
**T26**	**27**	Continuous	By-products (tripe, leg, trotter off, etc.)	Grams per day	
**T27**	**28**	Continuous	Pizza	Grams per day	
**T28**	**29**	Continuous	Milk	Grams per day	
**T29**	**30**	Continuous	Yogurt	Grams per day	
**T30**	**31**	Continuous	Cheese	Grams per day	
**T31**	**33**	Continuous	Doogh (Ayran)	Grams per day	
**T32**	**34**	Continuous	Kashk (Whey)	Grams per day	

**T33**	**155**	Continuous	Flavored milks (chocolate-milk, fruit-milk, coffee-milk, …)	Grams per day	
**T34**	**35**	Continuous	Lettuce	Grams per day	
**T35**	**36**	Continuous	Cabbage (Cauliflower, Broccoli,..)	Grams per day	
**T36**	**37**	Continuous	Tomatos	Grams per day	
**T37**	**38**	Continuous	Cucumbers	Grams per day	
**T38**	**39**	Continuous	Fresh leafy greens	Grams per day	
**T39**	**40**	Continuous	Cooked leafy greens	Grams per day	
**T40**	**41**	Continuous	Eggplant	Grams per day	
**T41**	**42**	Continuous	Celery	Grams per day	
**T42**	**43**	Continuous	Beets, Turnips	Grams per day	
**T43**	**44**	Continuous	Potatoes	Grams per day	
**T44**	**45**	Continuous	Carrots	Grams per day	
**T45**	**46**	Continuous	Garlic	Grams per day	
**T46**	**47**	Continuous	Onions	Grams per day	
**T47**	**48**	Continuous	Bell peppers	Grams per day	
**T48**	**49**	Continuous	Mushroom (cooked)	Grams per day	
**T49**	**50**	Continuous	Corn	Grams per day	
**T50**	**51**	Continuous	Green peas	Grams per day	
**T51**	**52**	Continuous	Green beans	Grams per day	
**T52**	**53**	Continuous	Zucchini	Grams per day	
**T53**	**139**	Continuous	Green peppers	Grams per day	
**T54**	**54**	Continuous	Cantaloup melon	Grams per day	
**T55**	**55**	Continuous	Honeydew	Grams per day	
**T56**	**56**	Continuous	Watermelon	Grams per day	
**T57**	**57**	Continuous	Apricot	Grams per day	
**T58**	**58**	Continuous	Sweet, Sour Cherries	Grams per day	
**T59**	**59**	Continuous	Nectarines, peaches	Grams per day	
**T60**	**60**	Continuous	Prunus	Grams per day	
**T61**	**61**	Continuous	Fresh Berries (Mulberries)	Grams per day	
**T62**	**62**	Continuous	Strawberries	Grams per day	
**T63**	**63**	Continuous	Plums	Grams per day	
**T64**	**64**	Continuous	Fresh Figs	Grams per day	
**T65**	**65**	Continuous	Grapes	Grams per day	
**T66**	**66**	Continuous	Pears	Grams per day	

**T67**	**67**	Continuous	Apples	Grams per day	
**T68**	**68**	Continuous	Kiwifruit	Grams per day	
**T69**	**69**	Continuous	Citrus fruit	Grams per day	
**T70**	**70**	Continuous	Pomegranate	Grams per day	
**T71**	**71**	Continuous	Banana	Grams per day	
**T72**	**72**	Continuous	Persimmon	Grams per day	
**T73**	**73**	Continuous	Dates	Grams per day	
**T74**	**74**	Continuous	Fruit juice (fresh)	Grams per day	
**T75**	**75**	Continuous	Dried Fruit	Grams per day	
**T76**	**76**	Continuous	Raisins	Grams per day	
**T77**	**140**	Continuous	Compote	Grams per day	
**T78**	**78**	Continuous	Margarine (vegetable butter)	Grams per day	
**T79**	**79**	Continuous	Butter	Grams per day	
**T80**	**80**	Continuous	Hydrogenated oils, animal fats	Grams per day	
**T81**	**81**	Continuous	Oils	Grams per day	
**T82**	**82**	Continuous	Olive oil	Grams per day	
**T83**	**83**	Continuous	Olives	Grams per day	
**T84**	**84**	Continuous	Mayonnaise, salad dressing	Grams per day	
**T85**	**85**	Continuous	Walnuts	Grams per day	
**T86**	**86**	Continuous	Peanut	Grams per day	
**T87**	**87**	Continuous	Other nuts (Almonds, Hindi, pistachios, hazelnuts)	Grams per day	
**T88**	**88**	Continuous	seeds (pumpkin watermelon, sunflower)	Grams per day	
**T89**	**141**	Continuous	Cream, clotted cream	Grams per day	
**T90**	**89**	Continuous	Sugar cubes	Grams per day	
**T91**	**90**	Continuous	Other sweets (rock candy, Noghl)	Grams per day	
**T92**	**91**	Continuous	Honey	Grams per day	
**T93**	**92**	Continuous	Jam	Grams per day	
**T94**	**142**	Continuous	Sugar (white)	Grams per day	
**T95**	**94**	Continuous	Tea	Grams per day	
**T96**	**95**	Continuous	Soda drinks	Grams per day	
**T97**	**96**	Continuous	Non-alcoholic malt beverage	Grams per day	
**T98**	**97**	Continuous	Coffee, Nescafe	Grams per day	
**T99**	**98**	Continuous	Ice-cream	Grams per day	
**T100**	**99**	Continuous	Cookies and Sweets (dry)	Grams per day	
**T101**	**100**	Continuous	Cookies and Sweets (creamed)	Grams per day	

**T102**	**101**	Continuous	Chocolate	Grams per day	
**T103**	**102**	Continuous	Chips	Grams per day	
**T104**	**103**	Continuous	Cheese Puffs	Grams per day	
**T105**	**104**	Continuous	Halva (Tahini)	Grams per day	
**T106**	**105**	Continuous	Pickles	Grams per day	
**T107**	**106**	Continuous	Torshi/Pickled vegetables (in vinegar)	Grams per day	
**T108**	**107**	Continuous	Tomato paste	Grams per day	
**T109**	**108**	Continuous	Juice (from concentrate)	Grams per day	
**T110**	**143**	Continuous	Crackers, wafers, biscuits	Grams per day	
**T111**	**144**	Continuous	Pickled vegetables (in salt water)	Grams per day	
**T112**	**145**	Continuous	Pomegranate paste	Grams per day	
**T113**	**111**	Continuous	Salt	Grams per day	

PERSIAN Cohort

Table Ta: Local Foods in the FFQ					
**V Code**	**Variable Name**	**Variable Type**	**Description**	**Coding/Unit**	**Comment***
**Ta1**	**156**	Continuous	Traditional Bread	Grams per day	Kermanshah
**Ta2**	**157**	Continuous	Kermanshahi Ghee	Grams per day	Kermanshah
**Ta3**	**136**	Continuous	Wheat	Grams per day	Kermanshah
**Ta4**	**137**	Continuous	Traditional Bread	Grams per day	Kermanshah
**Ta5**	**138**	Continuous	Cooked traditional greens	Grams per day	Kermanshah
**Ta6**	**123**	Continuous	Traditional Rice Bread	Grams per day	Guilan
**Ta7**	**124**	Continuous	Traditional Bread	Grams per day	Guilan
**Ta8**	**125**	Continuous	Common Beans	Grams per day	Guilan
**Ta9**	**126**	Continuous	Poultry meat (Other than chicken)	Grams per day	Guilan
**Ta10**	**127**	Continuous	Smoked/Salted fish	Grams per day	Guilan
**Ta11**	**128**	Continuous	Roe	Grams per day	Guilan
**Ta12**	**129**	Continuous	Daikon	Grams per day	Guilan
**Ta13**	**130**	Continuous	Garlic Leaves	Grams per day	Guilan
**Ta14**	**131**	Continuous	Common Medlar	Grams per day	Guilan
**Ta15**	**132**	Continuous	Enriched Olives	Grams per day	Guilan
**Ta16**	**133**	Continuous	Syrup	Grams per day	Guilan
**Ta17**	**134**	Continuous	Fruit Paste	Grams per day	Guilan
**Ta18**	**135**	Continuous	Darar (Green salt)	Grams per day	Guilan
**Ta19**	**112**	Continuous	Wheat	Grams per day	Fasa
**Ta20**	**113**	Continuous	Traditional Bread	Grams per day	Fasa
**Ta21**	**114**	Continuous	Poultry meat (Other than chicken)	Grams per day	Fasa
**Ta22**	**115**	Continuous	Colostrum	Grams per day	Fasa
**Ta23**	**116**	Continuous	Traditional Greens	Grams per day	Fasa
**Ta24**	**117**	Continuous	Lime Juice, Verjuice, sour orange juice	Grams per day	Fasa
**Ta25**	**118**	Continuous	Mango	Grams per day	Fasa
**Ta26**	**119**	Continuous	Baby Almonds	Grams per day	Fasa
**Ta27**	**120**	Continuous	Pistacia atlantica	Grams per day	Fasa
**Ta28**	**121**	Continuous	Traditional Sweets	Grams per day	Fasa
**Ta29**	**122**	Continuous	Sekanjabin	Grams per day	Fasa
**Ta30**	**109**	Continuous	Gaz (Traditional Sweet)	Grams per day	Fasa
**Ta31**	**93**	Continuous	Syrup	Grams per day	Azar
**Ta32**	**8**	Continuous	Traditional Bread	Grams per day	Azar
**Ta33**	**9**	Continuous	Traditional Bread	Grams per day	Azar

**Ta34**	**77**	Continuous	Quince	Grams per day	Azar
**Ta35**	**146**	Continuous	Traditional Bread	Grams per day	Kharameh
**Ta36**	**147**	Continuous	Poultry meat (Other than chicken)	Grams per day	Kharameh
**Ta37**	**148**	Continuous	Traditional Herbs	Grams per day	Kharameh
**Ta38**	**149**	Continuous	Pistacia atlantica	Grams per day	Kharameh
**Ta39**	**150**	Continuous	Sesame	Grams per day	Kharameh
**Ta40**	**151**	Continuous	Prunus scoparia (Wild Almond)	Grams per day	Kharameh
**Ta41**	**152**	Continuous	Thymes	Grams per day	Kharameh
**Ta42**	**153**	Continuous	Desert truffle (Terfeziaceae)	Grams per day	Kharameh
**Ta43**	**154**	Continuous	Traditional Herbs	Grams per day	Kharameh
**Ta44**	**158**	Continuous	Traditional Bread	Grams per day	Tabari
**Ta45**	**159**	Continuous	Darar (Green salt)	Grams per day	Tabari
**Ta46**	**160**	Continuous	Red Sugar	Grams per day	Tabari
**Ta47**	**161**	Continuous	Traditional Sweets	Grams per day	Tabari
**Ta48**	**162**	Continuous	Traditional Sweets	Grams per day	Tabari
**Ta49**	**172**	Continuous	Traditional Bread	Grams per day	Zahedan
**Ta50**	**173**	Continuous	Traditional Bread	Grams per day	Zahedan
**Ta51**	**174**	Continuous	Camel Meat	Grams per day	Zahedan
**Ta52**	**175**	Continuous	Kashk	Grams per day	Zahedan
**Ta53**	**176**	Continuous	Okra	Grams per day	Zahedan
**Ta54**	**177**	Continuous	Olive Fruit	Grams per day	Zahedan
**Ta55**	**178**	Continuous	Mango	Grams per day	Zahedan
**Ta56**	**179**	Continuous	Jujube	Grams per day	Zahedan
**Ta57**	**180**	Continuous	Sapodilla	Grams per day	Zahedan
**Ta58**	**181**	Continuous	Pineapple	Grams per day	Zahedan
**Ta59**	**182**	Continuous	Chilgoza Pine	Grams per day	Zahedan
**Ta60**	**183**	Continuous	Sugar Beet	Grams per day	Zahedan
**Ta61**	**184**	Continuous	Traditional Sweets	Grams per day	Zahedan
**Ta62**	**185**	Continuous	Traditional Sweets	Grams per day	Zahedan
**Ta63**	**186**	Continuous	Traditional Spices	Grams per day	Zahedan
**Ta64**	**187**	Continuous	Traditional Bread	Grams per day	Zahedan
**Ta65**	**188**	Continuous	Traditional Bread	Grams per day	Yazd
**Ta66**	**189**	Continuous	Tahini	Grams per day	Yazd
**Ta67**	**190**	Continuous	Syrup	Grams per day	Yazd
**Ta68**	**191**	Continuous	Wheat	Grams per day	Yazd

**Ta69**	**163**	Continuous	Traditional Dry Bread	Grams per day	Yazd
**Ta70**	**164**	Continuous	Rock Candy	Grams per day	Yazd
**Ta71**	**165**	Continuous	Cola	Grams per day	Yazd
**Ta72**	**166**	Continuous	Traditional Bread	Grams per day	Rafsanjan
**Ta73**	**167**	Continuous	Traditional Dry Bread	Grams per day	Rafsanjan
**Ta74**	**168**	Continuous	Pistacios	Grams per day	Rafsanjan
**Ta75**	**169**	Continuous	Traditional Sweets	Grams per day	Rafsanjan
**Ta76**	**170**	Continuous	Traditional Sweets	Grams per day	Rafsanjan
**Ta77**	**192**	Continuous	Rice Bread	Grams per day	Hoveizeh
**Ta78**	**193**	Continuous	Traditional Bread	Grams per day	Hoveizeh
**Ta79**	**194**	Continuous	Wheat	Grams per day	Hoveizeh
**Ta80**	**195**	Continuous	Traditional Herbs	Grams per day	Hoveizeh
**Ta81**	**196**	Continuous	Traditional Herb	Grams per day	Hoveizeh
**Ta82**	**197**	Continuous	Okra	Grams per day	Hoveizeh
**Ta83**	**198**	Continuous	Traditional Herb	Grams per day	Hoveizeh
**Ta84**	**199**	Continuous	Tahini	Grams per day	Hoveizeh
**Ta85**	**200**	Continuous	Date Syrup	Grams per day	Hoveizeh
**Ta86**	**201**	Continuous	Arabic Coffee	Grams per day	Hoveizeh
**Ta87**	**226**	Continuous	Traditional Bread	Grams per day	Shahrekord
**Ta88**	**227**	Continuous	Tahini	Grams per day	Shahrekord
**Ta89**	**228**	Continuous	Syrup	Grams per day	Shahrekord
**Ta90**	**202**	Continuous	Barley Bread	Grams per day	Bandare Kong
**Ta91**	**203**	Continuous	Traditional Bread	Grams per day	Bandare Kong
**Ta92**	**204**	Continuous	Salted Fish	Grams per day	Bandare Kong
**Ta93**	**205**	Continuous	Shrimp/Crab	Grams per day	Bandare Kong
**Ta94**	**206**	Continuous	Mahyawa (Fermented Fish Sauce)	Grams per day	Bandare Kong
**Ta95**	**207**	Continuous	Nido	Grams per day	Bandare Kong
**Ta96**	**208**	Continuous	Mango	Grams per day	Bandare Kong
**Ta97**	**209**	Continuous	Jujube	Grams per day	Bandare Kong
**Ta98**	**210**	Continuous	Indian-almond	Grams per day	Bandare Kong
**Ta99**	**211**	Continuous	Java plum	Grams per day	Bandare Kong
**Ta100**	**212**	Continuous	Date Syrup	Grams per day	Bandare Kong
**Ta101**	**213**	Continuous	Salted Fish Water	Grams per day	Bandare Kong
**Ta102**	**214**	Continuous	Red Soil From Hormoz Port (rich in iron)	Grams per day	Bandare Kong
**Ta103**	**215**	Continuous	Tamarind	Grams per day	Bandare Kong

**Ta104**	**216**	Continuous	Traditional Spices	Grams per day	Bandare Kong
**Ta105**	**217**	Continuous	Palm Oil	Grams per day	Bandare Kong
**Ta106**	**229**	Continuous	Traditional Bread	Grams per day	Urmia Lake
**Ta107**	**230**	Continuous	Syrup	Grams per day	Urmia Lake
**Ta108**	**218**	Continuous	Traditional Bread	Grams per day	Ardabil
**Ta109**	**219**	Continuous	Traditional Bread	Grams per day	Ardabil
**Ta110**	**220**	Continuous	Traditional Sweets	Grams per day	Ardabil
**Ta111**	**236**	Continuous	Traditional Bread	Grams per day	Sabzevar
**Ta112**	**237**	Continuous	Grape Syrup	Grams per day	Sabzevar
**Ta113**	**231**	Continuous	Starchy Noodles	Grams per day	Mashhad
**Ta114**	**232**	Continuous	Barberry	Grams per day	Mashhad
**Ta115**	**233**	Continuous	Sholeh Masshadi (Traditional Soup)	Grams per day	Mashhad
**Ta116**	**234**	Continuous	Saffron	Grams per day	Mashhad
**Ta117**	**235**	Continuous	Turmeric	Grams per day	Mashhad
**Ta118**	**221**	Continuous	Traditional Bread	Grams per day	Kavar
**Ta119**	**222**	Continuous	Desert truffle (Terfeziaceae)	Grams per day	Kavar
**Ta120**	**223**	Continuous	Meatless sausage/Salami	Grams per day	Kavar
**Ta121**	**224**	Continuous	Prunus scoparia (Wild Almond)	Grams per day	Kavar
**Ta122**	**225**	Continuous	Pistacia atlantica	Grams per day	Kavar
**Ta123**	**245**	Continuous	Traditional Bread	Grams per day	Dehgolan
**Ta124**	**246**	Continuous	Barley Bread	Grams per day	Dehgolan
**Ta125**	**247**	Continuous	Wheat and Sesame	Grams per day	Dehgolan
**Ta126**	**248**	Continuous	Traditional Herbs	Grams per day	Dehgolan
**Ta127**	**249**	Continuous	Syrup	Grams per day	Dehgolan
* Specifies the corresponding PERSIAN Cohort center for each local variable					

Table Tb: Nutrients*					
**V Code**	**Variable Name**	**Variable Type**	**Description**	**Coding/Unit**	**Comment**
**Tb1**	**Energy**	Continuous	Energy	Kilocalories per day	
**Tb2**	**Protein**	Continuous	Protein	Grams per day	
**Tb3**	**Total |lipid**	Continuous	Total lipid	Grams per day	
**Tb4**	**Carbohydrate**	Continuous	Carbohydrate	Grams per day	
**Tb5**	**Starch**	Continuous	Starch	Grams per day	
**Tb6**	**Sucrose**	Continuous	Sucrose	Grams per day	
**Tb7**	**Glucose**	Continuous	Glucose (dextrose)	Grams per day	
**Tb8**	**Fructose**	Continuous	Fructose	Grams per day	
**Tb9**	**Lactose**	Continuous	Lactose	Grams per day	
**Tb10**	**Maltose**	Continuous	Maltose	Grams per day	
**Tb11**	**Alcohol**	Continuous	Alcohol	Grams per day	
**Tb12**	**Caffeine**	Continuous	Caffeine	Milligrams per day	
**Tb13**	**Sugar**	Continuous	total Sugar	Grams per day	
**Tb14**	**Galactose**	Continuous	Galactose	Grams per day	
**Tb15**	**Fiber**	Continuous	total Fiber	Grams per day	
**Tb16**	**Calcium**	Continuous	Calcium	Milligrams per day	
**Tb17**	**Iron**	Continuous	Iron	Milligrams per day	
**Tb18**	**Magnesium**	Continuous	Magnesium	Milligrams per day	
**Tb19**	**Phosphorus**	Continuous	Phosphorus	Milligrams per day	
**Tb20**	**Potassium**	Continuous	Potassium	Milligrams per day	
**Tb21**	**Sodium**	Continuous	Sodium	Milligrams per day	
**Tb22**	**Zinc**	Continuous	Zinc	Milligrams per day	
**Tb23**	**Copper**	Continuous	Copper	Milligrams per day	
**Tb24**	**Fluoride**	Continuous	Fluoride	Micrograms per day	
**Tb25**	**Manganese**	Continuous	Manganese	Milligrams per day	
**Tb26**	**Selenium**	Continuous	Selenium	Micrograms per day	
**Tb27**	**Vitamin A**	Continuous	Vitamin A	International Units	
**Tb28**	**Retinol**	Continuous	Retinol	Micrograms per day	
**Tb29**	**Vitamin A**	Continuous	Vitamin A	Micrograms per day	
**Tb30**	**Beta Carotene**	Continuous	Beta Carotene	Micrograms per day	
**Tb31**	**Alpha Carotene**	Continuous	Alpha Carotene	Micrograms per day	
**Tb32**	**Vitamin E**	Continuous	Vitamin E (alpha tocopherol)	Milligrams per day	
**Tb33**	**Vitamin D**	Continuous	Vitamin D	International Units	

**Tb34**	**Vitamin D (D2D3)**	Continuous	Vitamin D (D2D3)	Micrograms per day	
**Tb35**	**Cryptoxanthin, beta**	Continuous	Cryptoxanthin, beta	Micrograms per day	
**Tb36**	**Lycopene**	Continuous	Lycopene	Micrograms per day	
**Tb37**	**Lutein + zeaxanthin**	Continuous	Lutein + zeaxanthin	Micrograms per day	
**Tb38**	**Vitamin C**	Continuous	Vitamin C (total ascorbic acid)	Milligrams per day	
**Tb39**	**Thiamin**	Continuous	Thiamin	Milligrams per day	
**Tb40**	**Riboflavin**	Continuous	Riboflavin	Milligrams per day	
**Tb41**	**Niacin**	Continuous	Niacin	Milligrams per day	
**Tb42**	**Pantothenic Acid**	Continuous	Pantothenic Acid	Milligrams per day	
**Tb43**	**Vitamin B6**	Continuous	Vitamin B6	Milligrams per day	
**Tb44**	**Folate**	Continuous	Total Folate	Micrograms per day	
**Tb45**	**Vitamin B12**	Continuous	Vitamin B12	Micrograms per day	
**Tb46**	**Choline, total**	Continuous	Choline, total	Milligrams per day	
**Tb47**	**Vitamin K**	Continuous	Vitamin K	Micrograms per day	
**Tb48**	**Betaine**	Continuous	Betaine	Milligrams per day	
**Tb49**	**Tryptophan**	Continuous	Tryptophan	Grams per day	
**Tb50**	**Threonine**	Continuous	Threonine	Grams per day	
**Tb51**	**Isoleucine**	Continuous	Isoleucine	Grams per day	
**Tb52**	**Leucine**	Continuous	Leucine	Grams per day	
**Tb53**	**Lysine**	Continuous	Lysine	Grams per day	
**Tb54**	**Methionine**	Continuous	Methionine	Grams per day	
**Tb55**	**Cysteine**	Continuous	Cysteine	Grams per day	
**Tb56**	**Phenylalanine**	Continuous	Phenylalanine	Grams per day	
**Tb57**	**Tyrosine**	Continuous	Tyrosine	Grams per day	
**Tb58**	**Valine**	Continuous	Valine	Grams per day	
**Tb59**	**Arginine**	Continuous	Arginine	Grams per day	
**Tb60**	**Histidine**	Continuous	Histidine	Grams per day	
**Tb61**	**Alanine**	Continuous	Alanine	Grams per day	
**Tb62**	**Aspartic Acid**	Continuous	Aspartic Acid	Grams per day	
**Tb63**	**Glutamic Acid**	Continuous	Glutamic Acid	Grams per day	
**Tb64**	**Glycine**	Continuous	Glycine	Grams per day	
**Tb65**	**Proline**	Continuous	Proline	Grams per day	
**Tb66**	**Serine**	Continuous	Serine	Grams per day	
**Tb67**	**Cholesterol**	Continuous	Cholesterol	Milligrams per day	
**Tb68**	**Fatty Acid (total trans)**	Continuous	Total trans fatty Acid	Grams per day	

**Tb69**	**Fatty Acid (total saturated)**	Continuous	Total saturated fatty Acid	Grams per day	
**Tb70**	**Fatty acids, MUFA**	Continuous	Fatty acids, total monounsaturated	Grams per day	
**Tb71**	**Fatty acids, PUFA**	Continuous	Fatty acids, total polyunsaturated	Grams per day	
**Tb72**	**n-3 (total)**	Continuous	Fatty acids, Omega-3, total	Grams per day	
**Tb73**	**Docosahexaenoic Acid (DHA)**	Continuous	Fatty acids, Docosahexaenoic Acid	Grams per day	
**Tb74**	**Eicosapentaenoic Acid (EPA)**	Continuous	Fatty acids, Eicosapentaenoic Acid	Grams per day	
**Tb75**	**Alpha-Linolenic Acid (ALA)**	Continuous	Fatty acids, Alpha-Linolenic Acid	Grams per day	
**Tb76**	**n-6 (total)**	Continuous	Fatty acids, Omega-6, total	Grams per day	
* All varibales in this table (Tb1-Tb76) can be calculated for any subset of variables from the FFQ (table T).					

PERSIAN Cohort

**Table U: Dietary Habits**					
**V Code**	**Variable Name**	**Variable Type**	**Description**	**Coding/Unit**	**Comment**
**U1**	**EatIntID**	Categorical	The number of meals usually eaten in a day	1= 3 (breakfast, lunch, and dinner)<br>2= 4 (breakfast, lunch, dinner and one snack)<br>3= 5-6 (3 meals and 2-3 snacks)<br>4= More than 6<br>5= Less than 3	
**U2**	**SaltUseID**	Categorical	Adding salt to food at the table	1= Yes<br>2= Sometimes<br>3= No	
**U3**	**FoodSaltUsedID**	Categorical	Saltiness of food as perceived by participant	1= Little salt<br>2= Regular<br>3= Salty	
**U4**	**GrilledFoodIntID**	Categorical	Frequency of eating barbecued foods and Kebabs (cooking on fire/grill)	0= Never<br>1= Less than once per month<br>2= 1-3 time per month<br>3= 1-3 time per week<br>4= Daily	
**U5**	**FriedFoodIntID**	Categorical	Frequency of eating fried foods	0= Never<br>1= Less than once per month<br>2= 1-3 time per month<br>3= 1-3 time per week<br>4= Daily	
**U6**	**PotatoFryTypeID**	Categorical	Usual amount of frying done to fry potatoes, eggplants and zucchini	1= Little frying, Sautéing<br>2= Medium amount of frying, until golden yellow<br>3= A lot of frying, until browned<br>4= Does not fry foods	
**U7**	**OnionFryTypeID**	Categorical	Usual amount of frying done to fry onions	1= Little frying, Sautéing<br>2= Medium amount of frying, until golden yellow<br>3= A lot of frying, until browned<br>4= Does not fry foods	
**U8**	**VegFryTypeID**	Categorical	Usual amount of frying done to fry vegetables	1= Little frying, Sautéing<br>2= Medium amount of frying, until golden yellow<br>3= A lot of frying, until browned<br>4= Does not fry foods	
**U9**	**UsedOilTypeID**	Categorical	Type of oil used for frying	1= Solid oil (Ghee)<br>2= Semi-solid oil (Margarine)<br>3= Liquid oil<br>4= Frying oil<br>5= Other types of oil	

				6= Does not fry foods	
**U10**	**ReUseOil**	Dichotomous	Reusing oil after frying	0= No, 1= Yes	
**U11**	**ReUseOilNo**	Discrete	Specifies the number of times oil is re-used if "Yes" is reported for variable U10		
**U12**	**ReUseMold**	Dichotomous	Using jam, pickles, tomato paste, and other foods even after they have molded (removing the mold and using the rest)	0= No, 1= Yes	
**U13**	**SmokedFoodIntID**	Categorical	Frequency of using smoked rice, fish, and other smoked products	0= Never<br>1= Less than once per month<br>2= 1-3 time per month<br>3= 1-3 time per week<br>4= Daily	
**U14**	**VegKeepDried**	Dichotomous	Storing vegetables dried	0= No, 1= Yes	
**U15**	**VegKeepRefrigerator**	Dichotomous	Storing vegetables in the refrigerator	0= No, 1= Yes	
**U16**	**VegKeepFreezer**	Dichotomous	Storing vegetables frozen	0= No, 1= Yes	
**U17**	**VegKeepFresh**	Dichotomous	Does not store vegetables (only uses fresh)	0= No, 1= Yes	
**U18**	**FreezVegTypeID**	Categorical	The way vegetables are stored in the refrigerator/freezer, if “Yes” is reported for variables U15 or U16	1= Raw<br>2= Boiled<br>3= Fried	
**U19**	**FreezMeatTypeID**	Categorical	The way meats and meat products are stored in the refrigerator/freezer	1= Raw<br>2= Boiled<br>3= Fried<br>4= Not stored (only fresh is used)	
**U20**	**TeaTempUseID**	Categorical	The usual temperature of tea/coffee when participant consumes it	1= Hot<br>2= Lukewarm<br>3= Cold<br>4= Doesn't drink tea/coffee	
**U21**	**SoupTempUseID**	Categorical	The usual temperature of soups/other liquid foods when participant consumes it	1= Hot<br>2= Lukewarm<br>3= Cold<br>4= Doesn't consume soup/liquid foods	
**U22**	**WaterContainerTypeID1**	Categorical	Type of container used to store water (#1)	1= Plastic<br>2= Steel<br>3= Chinaware<br>4= Glass<br>5= Other	Up to two containers, that are used more often than other types, could have been reported.
**U23**	**WaterContainerTypeID2**	Categorical	Type of container used to store water (#2)	1= Plastic<br>2= Steel<br>3= Chinaware<br>4= Glass	

				5= Other	
**U24**	**BreadContainerTypeID1**	Categorical	Type of container used to store bread (#1)	1= Plastic<br>2= Steel<br>3= Cloth<br>4= Glass<br>5= Other<br>6= Doesn't store bread	Up to two containers, that are used more often than other types, could have been reported.
**U25**	**BreadContainerTypeID2**	Categorical	Type of container used to store bread (#2)	1= Plastic<br>2= Steel<br>3= Cloth<br>4= Glass<br>5= Other<br>6= Doesn't store bread	
**U26**	**LemonContainerTypeID1**	Categorical	Type of container used to store lemon juice, lime juice or verjuice (#1)	1= Plastic<br>2= Steel<br>4= Glass<br>5= Other<br>6= Doesn't store these variables	Up to two containers, that are used more often than other types, could have been reported.
**U27**	**LemonContainerTypeID2**	Categorical	Type of container used to store lemon juice, lime juice or verjuice (#2)	1= Plastic<br>2= Steel<br>4= Glass<br>5= Other<br>6= Doesn't store these variables	
**U28**	**TomatoContainerTypeID1**	Categorical	Type of container used to store tomato juice, tomato paste (#1)	1= Plastic<br>2= Steel<br>4= Glass<br>5= Other<br>6= Doesn't store these variables	Up to two containers, that are used more often than other types, could have been reported.
**U29**	**TomatoContainerTypeID2**	Categorical	Type of container used to store tomato juice, tomato paste (#2)	1= Plastic<br>2= Steel<br>4= Glass<br>5= Other<br>6= Doesn't store these variables	
**U30**	**PicklesContainerTypeID1**	Categorical	Type of container used to store pickles and pickled vegetables (Torshi) (#1)	1= Plastic<br>2= Steel<br>4= Glass<br>5= Other<br>6= Doesn't store these variables	Up to two containers, that are used more often than other types, could have been reported.
**U31**	**PicklesContainerTypeID2**	Categorical	Type of container used to store pickles and pickled vegetables (Torshi) (#2)	1= Plastic<br>2= Steel<br>4= Glass<br>5= Other<br>6= Doesn't store these variables	

**U32**	**CookingWareID1**	Categorical	Type of cooking ware (pots and pans) used (#1)	1= Zinc coated<br>2= Copper<br>3= Aluminum<br>4= Glazed<br>5= Teflon<br>6= Cast Iron<br>7= Steel<br>8= Pyrex<br>9= Other	
**U33**	**CookingWareID2**	Categorical	Type of cooking ware (pots and pans) used (#2)	1= Zinc coated<br>2= Copper<br>3= Aluminum<br>4= Glazed<br>5= Teflon<br>6= Cast Iron<br>7= Steel<br>8= Pyrex<br>9= Other	Up to three types of cookware, that are used more often than other types, could have been reported.
**U34**	**CookingWareID3**	Categorical	Type of cooking ware (pots and pans) used (#3)	1= Zinc coated<br>2= Copper<br>3= Aluminum<br>4= Glazed<br>5= Teflon<br>6= Cast Iron<br>7= Steel<br>8= Pyrex<br>9= Other	
**U35**	**UsedScratchedTeflon**	Dichotomous	Use of scratched Teflon cooking ware, if "Teflon" is reported as one of the cooking ware types used in variables U32-U34	0= No, 1= Yes	
**U36**	**EatingDishwareID1**	Categorical	Type of dishware used (#1)	1= China<br>2= Aluminum<br>3= Glazed<br>4= Melamine<br>5= Steel<br>6= Plastic<br>7= Glass<br>8= Arcopal<br>9= Other	Up to three types of dishware, that are used more often than other types, could have been reported.
**U37**	**EatingDishwareID2**	Categorical	Type of dishware used (#1)	1= China<br>2= Aluminum<br>3= Glazed<br>4= Melamine<br>5= Steel	

				6= Plastic<br>7= Glass<br>8= Arcopal<br>9= Other	
**U38**	**EatingDishwareID3**	Categorical	Type of dishware used (#1)	1= China<br>2= Aluminum<br>3= Glazed<br>4= Melamine<br>5= Steel<br>6= Plastic<br>7= Glass<br>8= Arcopal<br>9= Other	
**U39**	**StoringContainerID1**	Categorical	Type of container used to keep leftovers (#1)	1= Copper<br>2= China<br>3= Aluminum<br>4= Glazed<br>5= Melamine<br>6= Steel<br>7= Plastic<br>8= Glass<br>9= Other<br>10= Doesn't keep left-overs	Up to three types of dishware, that are used more often than other types, could have been reported.
**U40**	**StoringContainerID2**	Categorical	Type of container used to keep leftovers (#2)	1= Copper<br>2= China<br>3= Aluminum<br>4= Glazed<br>5= Melamine<br>6= Steel<br>7= Plastic<br>8= Glass<br>9= Other<br>10= Doesn't keep left-overs	
**U41**	**StoringContainerID3**	Categorical	Type of container used to keep leftovers (#3)	1= Copper<br>2= China<br>3= Aluminum<br>4= Glazed<br>5= Melamine<br>6= Steel<br>7= Plastic<br>8= Glass<br>9= Other<br>10= Doesn't keep left-overs	
**U42**	**FoodAlergy**	Characteristic	List of any food allergies		Both diagnosed or perceived

**U43**	**UseBoiledHerbal**	Dichotomous	Use of herbal teas or herbal powders	0= No, 1= Yes	
**U44**	**UsedHerbal**	Characteristic	The type of herbal tea or powders used, if " Yes " is reported for variable U43		
**U45**	**WhyUseHerbal**	Characteristic	Reason for using herbal tea or powders, if " Yes " is reported for variable U43		
**U46**	**Description**	Characteristic	Any related comments		

PERSIAN Cohort

Table V: Water Use					
V Code	Variable Name	Variable Type	Description	Coding/Unit	Comment
**V1**	**Water_inSummer**	Discrete	The number of glasses the participant drinks water in the summer, in the time frame indicated in V2	Glass of water (230 ml)	Variables V1-V2 are linked and specify the glasses of water the participant drinks during a given time frame.
**V2**	**Water_SummerInterval**	Categorical	Time frame in which participant usually drinks water in the summer	1= In a day<br>2= In a week<br>3= In a month	
**V3**	**Water_inOther**	Discrete	The number of glasses the participant drinks water in the other seasons, in the time frame indicated in V4	Glass of water (230 ml)	Variables V3-V4 are linked and specify the glasses of water the participant drinks during a given time frame.
**V4**	**Water_OtherInterval**	Categorical	Time frame in which participant usually drinks water in other seasons	1= In a day<br>2= In a week<br>3= In a month	

PERSIAN Cohort

Table W: Dietary Supplements					
**V Code**	**Variable Name**	**Variable Type**	**Description**	**Coding/Unit**	**Comment**
**W1**	**SupMultiVitaminMineralInterval**	Categorical	Taking multi-vitamin mineral supplements	0= Never<br>1= Daily<br>2= Weekly<br>3= Monthly<br>4= Annually	
**W2**	**SupMultiVitaminMineralValue**	Discrete	Specifies the number of multi-vitamin/mineral supplements taken over the time frame indicated in variable W1		
**W3**	**SupMultiVitaminInterval**	Categorical	Taking multi-vitamin supplements	0= Never<br>1= Daily<br>2= Weekly<br>3= Monthly<br>4= Annually	
**W4**	**SupMultiVitaminValue**	Discrete	Specifies the number of multi-vitamin supplements taken over the time frame indicated in variable W3		
**W5**	**SupCalciumDInterval**	Categorical	Taking calcium + vitamin D supplements	0= Never<br>1= Daily<br>2= Weekly<br>3= Monthly<br>4= Annually	
**W6**	**SupCalciumDValue**	Discrete	Specifies the number of calcium + vitamin D supplements taken over the time frame indicated in variable W5		
**W7**	**SupCalciumInterval**	Categorical	Taking calcium supplements	0= Never<br>1= Daily<br>2= Weekly<br>3= Monthly<br>4= Annually	
**W8**	**SupCalciumValue**	Discrete	Specifies the number of calcium supplements taken over the time frame indicated in variable W7		
**W9**	**SupVitaminDPillInterval**	Categorical	Taking vitamin D supplement pills	0= Never<br>1= Daily<br>2= Weekly<br>3= Monthly<br>4= Annually	
**W10**	**SupVitaminDPillValue**	Discrete	Specifies the number of vitamin D supplements taken over the time frame indicated in variable W9		
**W11**	**SupVitaminDVialInterval**	Categorical	Receiving vitamin D injection supplements	0= Never<br>1= Daily<br>2= Weekly<br>3= Monthly<br>4= Annually	

**W12**	**SupVitaminDVialValue**	Discrete	Specifies the number of vitamin D injections over the time frame indicated in variable W11		
**W13**	**SupFolicAcidInterval**	Categorical	Taking folic acid supplements	0= Never<br>1= Daily<br>2= Weekly<br>3= Monthly<br>4= Annually	
**W14**	**SupFolicAcidValue**	Discrete	Specifies the number of folic acid supplements taken over the time frame indicated in variable W13		
**W15**	**SupOmega3Interval**	Categorical	Taking omega-3 or fish oil supplements	0= Never<br>1= Daily<br>2= Weekly<br>3= Monthly<br>4= Annually	
**W16**	**SupOmega3Value**	Discrete	Specifies the number of omega-3 or fish oil supplements taken over the time frame indicated in variable W15		
**W17**	**SupIronInterval**	Categorical	Taking iron supplements	0= Never<br>1= Daily<br>2= Weekly<br>3= Monthly<br>4= Annually	
**W18**	**SupIronValue**	Discrete	Specifies the number of iron supplements taken over the time frame indicated in variable W17		
**W19**	**SupZincInterval**	Categorical	Taking zinc supplements	0= Never<br>1= Daily<br>2= Weekly<br>3= Monthly<br>4= Annually	
**W20**	**SupZincValue**	Discrete	Specifies the number of zinc supplements taken over the time frame indicated in variable W20		
**W21**	**SupOtherVitaminInterval**	Categorical	Taking a vitamin/mineral supplement other than those previously specified	0= Never<br>1= Daily<br>2= Weekly<br>3= Monthly<br>4= Annually	
**W22**	**SupOtherVitaminValue**	Discrete	Specifies the number of other supplements taken over the time frame indicated in variable W22		
**W23**	**Description**	Characteristic	Any related comments		
* For all supplement reported, amount taken over the year previous to the enrollment date were recorded.					

Table X: Biobank Samples					
**V Code**	**Variable Name**	**Variable Type**	**Description**	**Coding/Unit**	**Comment**
**X1**	**HasUrine**	Dichotomous	Specifies if participant has urine sample	0= No, 1= Yes	
**X2**	**HasBlood**	Dichotomous	Specifies if participant has blood sample	0= No, 1= Yes	
**X3**	**HasHair**	Dichotomous	Specifies if participant has hair sample	0= No, 1= Yes	
**X4**	**HasNail**	Dichotomous	Specifies if participant has nail sample	0= No, 1= Yes	
**X5**	**Description**	Characteristic	Any related comments		

PERSIAN Cohort

**Table Y: Lab Test Results**					
**V Code**	**Variable Name**	**Variable Type**	**Description**	**Coding/Unit**	**Comment**
**Y1**	**TestDate**	Date	Specifies the date of sampling		
**Y2**	**WBC**	Continuous	White blood cell count	Cumm	
**Y3**	**RBC**	Continuous	Red blood cell count	Cumm	
**Y4**	**HGB**	Continuous	Hemoglobin	Gr/dl	
**Y5**	**HCT**	Continuous	Hematocrit	Gr/dl	
**Y6**	**MCV**	Continuous	Mean corpuscular volume	FL	
**Y7**	**MCH**	Continuous	Mean corpuscular hemoglobin	Pg	
**Y8**	**MCHC**	Continuous	Mean corpuscular hemoglobin concentration	Gr/dl	
**Y9**	**PLT**	Continuous	Platelet count	Cumm	
**Y10**	**LY**	Continuous	Lymphocytes	%	
**Y11**	**MO**	Continuous	Monocytes	%	
**Y12**	**GR**	Continuous	Granulocytes	%	
**Y13**	**RDWCV**	Continuous	Red cell distribution width	%	
**Y14**	**PCT**	Continuous	Plateletcrit	%	
**Y15**	**MPV**	Continuous	Mean platelet volume	FL	
**Y16**	**PDW**	Continuous	Platelet distribution width	%	
**Y17**	**GLUC**	Continuous	Fasting blood sugar	Mg/dl	
**Y18**	**BUN**	Continuous	Blood urea nitrogen	Mg/dl	
**Y19**	**Creat**	Continuous	Level of creatinine	Mg/dl	
**Y20**	**TG**	Continuous	Triglyceride	Mg/dl	
**Y21**	**CHOL**	Continuous	Total cholesterol	Mg/dl	
**Y22**	**HDLC**	Continuous	High-density lipoprotein	Mg/dl	
**Y23**	**LDL**	Continuous	Low-density lipoprotein	Mg/dl	
**Y24**	**LDL_Calc**	Continuous	Calculated Low-density lipoprotein	Mg/dl	Calculated using Friedewald formula
**Y25**	**SGOT**	Continuous	The aspartate aminotransferase (AST)	IU/L	
**Y26**	**SGPT**	Continuous	The alanine aminotransferase (ALT)	IU/L	
**Y27**	**ALP**	Continuous	Alkaline phosphatase	IU/L	
**Y28**	**GGT**	Continuous	Gamma-glutamyl transpeptidase	IU/L	
**Y29**	**eGFR**	Continuous	Glomerular filtration rate	$mL/min/1.73m^2$	Calculated using MDRD formula
**Y30**	**Color**	Categorical	Urine color	1= Yellow<br>2= Brown	

				3= Red<br>4= White	
**Y31**	**SG**	Continuous	Specific gravity	µg	
**Y32**	**Appearance**	Categorical	Urine appearance	1= Clear<br>2= Semi clear<br>3= Turbid	
**Y33**	**PH**	Continues	Urine pH level		
**Y34**	**Nitrite**	Categorical	Urine nitrite	1= Negative<br>2= Positive<br>3= Positive+<br>4= Positive++<br>5= Positive+++	
**Y35**	**Bilirubin**	Categorical	Urine bilirubin	1= Negative<br>2= Positive<br>3= Positive+<br>4= Positive++<br>5= Positive+++	
**Y36**	**Urobilinogen**	Categorical	Urine urobilinogen	1= Negative<br>2= Positive<br>3= Positive+<br>4= Positive++<br>5= Positive+++	
**Y37**	**Protein**	Categorical	Urine protein	1= Negative<br>2= Positive<br>3= Positive+<br>4= Positive++<br>5= Positive+++	
**Y38**	**Glucose**	Categorical	Urine glucose	1= Negative<br>2= Positive<br>3= Positive+<br>4= Positive++<br>5= Positive+++	
**Y39**	**Blood**	Categorical	Urine blood	1= Negative<br>2= Positive<br>3= Positive+<br>4= Positive++<br>5= Positive+++	
**Y40**	**Epithelial**	Categorical	Epithelial cells in urine	1= Negative or few<br>2= Moderate<br>3= Many	
**Y41**	**Bacteria**	Categorical	Bacteria in urine	1= Negative or few<br>2= Moderate<br>3= Many	
**Y42**	**Mucus**	Categorical	Mucus in urine	1= Negative or few	

				2= Moderate<br>3= Many	
**Y43**	**Cast**	Categorical	Cast in urine	1= Negative or few<br>2= Moderate<br>3= Many	
**Y44**	**Crystal**	Categorical	Crystal in urine	1= Negative or few<br>2= Moderate<br>3= Many	
**Y45**	**AscorbicAcid**	Categorical	Urine ascorbic acid level	1= Negative<br>2= Positive+<br>3= Positive++<br>4= Positive+++	
**Y46**	**KetoneBodies**	Categorical	Ketone bodies in urine	1= Negative<br>2= Positive+<br>3= Positive++<br>4= Positive+++	
**Y47**	**UrineWBC**	Continues	Urine white blood cell count	1= Negative or few<br>2= Moderate<br>3= Many	
**Y48**	**UrineRBC**	Continues	Urine red blood cell count	1= Negative or few<br>2= Moderate<br>3= Many	
**Y49**	**Alb**	Continues	Albumin in urine	1= Negative<br>2= Positive+<br>3= Positive++<br>4= Positive+++	
	**Creat**	Continues	Creatinine in urine	Mg/dl	
	**Other**	Characteristic	Any related comments		

PERSIAN Cohort

Supplementary Figures and Tables

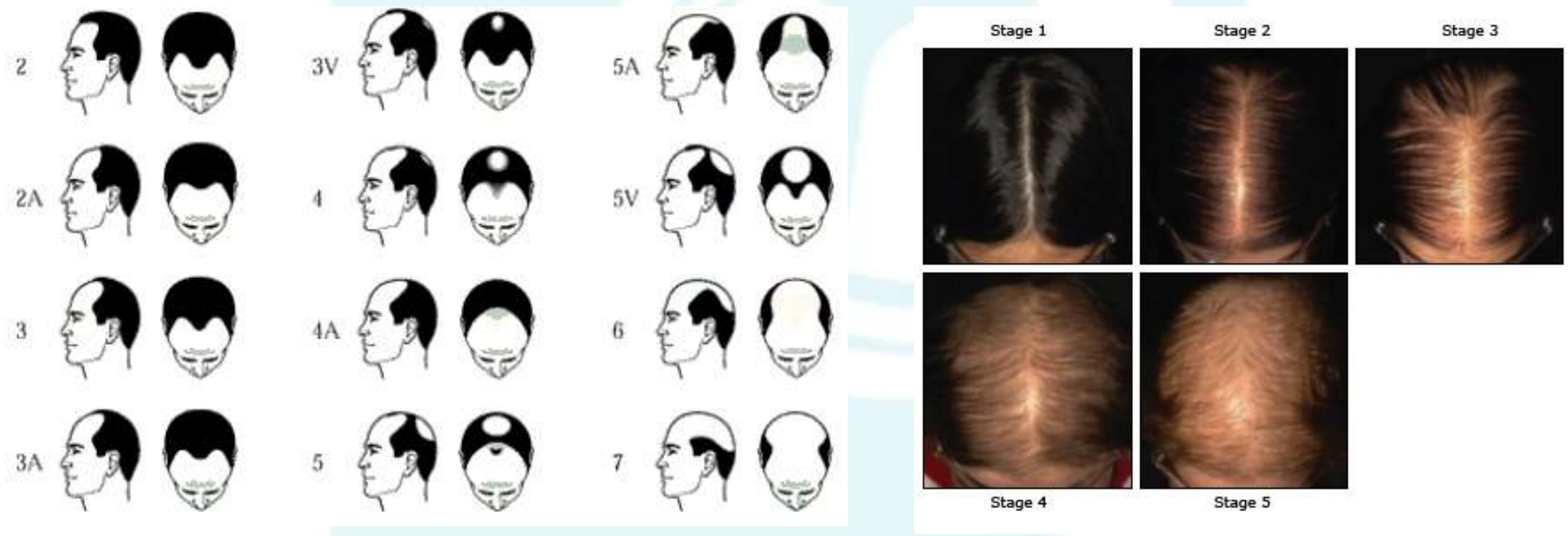

Men ***Women***

Figure S1: The stages of hair loss in men and women

PERSIAN Cohort

Table S1: Province ID							
Code	Province name		Code	Province name		Code	Province name
1	East Azerbaijan		12	North Khorasan		23	Golestan
2	West Azerbaijan		13	Zanjan		24	Guilan
3	Ilam		14	Semnan		25	Lorestan
4	Ardabil		15	Sistan & Baluchestan		26	Mazandaran
5	Isfahan		16	Fars		27	Markazi
6	Bushehr		17	Qom		28	Hamedan
7	Tehran		18	Qazvin		29	Hormozgan
8	Chaharmahal and Bakhtiari		19	Kohgiluyeh and Boyer-Ahmad		30	Yazd
9	Khuzestan		20	Kerman		31	Alborz
10	Razavi Khorasan		21	Kermanshah			
11	South Khorasan		22	Kurdestan			

PERSIAN Cohort

Table S2: County ID

Code	County Name	Code	County Name	Code	County Name
355	Shiraz	386	Bonab	417	Namin
356	KhoramAbaad	387	Jolfa	418	Nir
357	Kerman	388	Sarab	419	Germi
358	Yazd	389	Shabestar	420	Aran & Bidgol
359	Sari	390	Ajab Shir	421	Ardestan
360	Kermanshah	391	Kaleybar	422	Shahin Shahr and Meimeh
361	Kashan	392	Maraghi	423	Tiran and Karvan
362	Shahrekord	393	Marand	424	Chadegan
363	Karaj	394	Malekan	425	Khomeini Shahr
364	Urmia	395	Mianeh	426	Khansar
365	Isfahan	396	Heris	427	Semirom
366	Mashhad	397	Hasht-roud	428	Shahreza
367	Tabriz	398	Varzaghan	429	Semirom Sofla
368	Qom	399	Oshnavieh	430	Fereydoun
369	Arak	400	Bukan	431	Fereydoun Shahr
370	Semnan	401	Piranshahr	432	Falavarjan
371	Hamedan	402	Takab	433	Golpayegan
372	Tehran	403	Chaldoran	434	Lenjan
373	Ardabil	404	Khoy	435	Mobarakeh
374	Bushehr	405	Sardasht	436	Naein
375	Ahvaz	406	Salmas	437	Najaf Abaad
376	Bandare Abbas	407	Shahin-Dej	438	Natanz
377	Rasht	408	Makoo	439	Deyr
378	Babol	409	Mahabaad	440	Deylam
379	Tonekabon	410	Mian-doab	441	Kangan
380	Gorgan	411	Naghadeh	442	Gonaveh
381	Qazvin	412	Bileh Savar	443	Tangestan
382	Azarshahr	413	ParsAbaad	444	Jam
383	Oskoo	414	Khalkhal	445	Dashtestan
384	Ahar	415	Kowsar	446	Dashti
385	BostanAbaad	416	Meshkin Shahr	447	Abdanan

Code	County Name	Code	County Name	Code	County Name
448	Eyvan	479	Taybad	510	Izeh
449	Dareh Shahr	480	Torbate Jam	511	Bagh Malek
450	Dehloran	481	Torbate Heidarieh	512	Mahshahr
451	Mehran	482	Chenaran	513	Behbahan
452	Ilam	483	Joghatai	514	Khoramshahr
453	Islam Shahr	484	Jovin	515	Dezfoul
454	Pakdasht	485	Khalil Abaad	516	Dashte Azadegan
455	Damavand	486	Khaf	517	Ramhormoz
456	Robat Karim	487	Dargaz	518	Shadegan
457	Rey	488	Rasht-Khar	519	Shoush
458	Savojbolagh	489	Zaveh	520	Shoushtar
459	Shemiranat	490	Sabzevar	521	Lali
460	Shahriar	491	Sarakhs	522	Masjed Soleiman
461	Firouz Kouh	492	Fariman	523	Hendijan
462	Nazar Abaad	493	Ghouchan	524	Abhar
463	Varamin	494	Torghabeh & Shandiz	525	Ijroud
464	Ardal	495	Kashmar	526	Khodabandeh
465	Boroujen	496	Kalat	527	Khordam-Dareh
466	Farsan	497	Gonabad	528	Zanjan
467	Kouhrang	498	Mahvelat	529	Taram
468	Lordegan	499	Neyshabour	530	Mahneshan
469	Boshruyeh	500	Esfarayen	531	Damghan
470	Birjand	501	Bojnourd	532	Shahroud
471	Darmian	502	Jajarm	533	Garmsar
472	Sarayan	503	Shiravan	534	Mahdi Shahr
473	Sarbisheh	504	Farouj	535	Irani Shahr
474	Ferdous	505	Garmeh	536	Chabahar
475	Ghaen	506	Maneh Va Samalqan	537	Khash
476	Nehbandan	507	Abadan	538	Zabol
477	Bardaskan	508	Omidieh	539	Zahedan
478	Bajestan	509	Andimeshk	540	Saravan

Code	County Name	Code	County Name	Code	County Name
541	Sarbaz	572	Marivan	603	Bahma'i
542	Nikshahr	573	Baft	604	Dena
543	Abadeh	574	Bardsir	605	Kohgiluyeh
544	Arsenjan	575	Bam	606	Gachsaran
545	Estahban	576	Jiroft	607	Azadshahr
546	Eqlid	577	Ravar	608	Agh-Qala
547	Bavanat	578	Rafsanjan	609	Bandar-e-Gaz
548	Jahrom	579	Rudbar	610	Bandare Torkaman
549	Khordambid	580	Jonoub	611	Ramiyan
550	Darab	581	Zarand	612	Ali Abaad
551	Zarin-Dasht	582	Sirjan	613	Kord-Kouy
552	Sepidan	583	Shahre Babak	614	Kalaleh
553	Farashband	584	Anbar Abaad	615	Gonbad
554	Fasa	585	Ghal'e Ganj	616	Minou Dasht
555	Firouz-Abaad	586	Kouhbanan	617	Astara
556	Ghirokarzin	587	Kohnouj	618	Astaneh Ashrafieh
557	Kazeroun	588	Manoojan	619	Amlash
558	Larestan	589	Islam Abaad Gharb	620	Bandare Anzali
559	Lamerd	590	Paveh	621	Rezvanshahr
560	Marvdasht	591	Salase Babajani	622	Rudbar
561	Mamasani	592	Javan Roud	623	Rudsar
562	Mehr	593	Dalahou	624	Siah Kol
563	Ney-riz	594	Ravansar	625	Shaft
564	Baneh	595	Sar-Pole Zahab	626	Some'e Sara
565	Bijar	596	Sonqor	627	Tavalesh (Talesh)
566	Divan-Dareh	597	Sahneh	628	Fouman
567	Sarv-Abaad	598	Ghasre Shirin	629	Lahijan
568	Saghez	599	Kangavar	630	Langroud
569	Sanandaj	600	Guilan Gharb	631	Masal
570	Ghorveh	601	Harsin	632	Azna
571	Kamyaran	602	Boyer-Ahmad	633	Aligoudarz

Code	County Name	Code	County Name	Code	County Name
634	Boroojerd	665	Jusk	696	Garm-Dareh
635	Poldokhtar	666	Gheshm	697	Kouhsar
636	Doroud	667	Haji Abaad	698	Chahar-Bagh
637	Delfan	668	Abu-Mousa	699	Taleghan
638	Selseleh	669	Bastak	700	Sari
639	Kouhdasht	670	Gav-bandi	701	Doroud
640	Amol	671	AsadAbaad	702	Kelardasht
641	Babolsar	672	Bahar	703	Kharameh
642	Behshahr	673	Tuyserkan	704	Hoveizeh
643	Jouybar	674	Razan	705	Gotvand
644	Chalous	675	KaboudarAhang	706	Ben
645	Ramsar	676	Malayer	707	Saman
646	Savad-Kouh	677	Nahavand	708	Kiyar
647	Ghaem-shahr	678	Abarkouh	709	Bandare Khamir
648	Galougah	679	Ardakan	710	Bandare Kong
649	Mahmoud Abaad	680	Bafgh	711	Andika
650	Neka	681	Taft	712	Bavi
651	Nour	682	Khatam	714	Karoun
652	Noshahr	683	Sadough	715	Hamidieh
653	Ashtian	684	Tabas	716	Dehdez
654	Tafresh	685	Mehriz	717	Ramshir
655	Khomein	686	Meybod	718	Haftgol
656	Delijan	687	Marvast	719	Lali
657	Zarandieh	688	Lavasan	721	Aghajri
658	Saveh	689	Hasht-Gaerd	722	Kavar
659	Shazand	690	Mohammad Shahr	723	Sysakht
660	Komijan	691	Kamal Shahr	724	Dehgolan
661	Mahalat	692	Meshkin Dasht		
662	Minab	693	Mah Dasht		
663	Bandare Lengaeh	694	Eshtehard		
664	Roudan	695	Hashg-Gaerd New City		

Table S3: Medication ID

Code	Drug name	Code	Drug name
1	Permethrin 1%	35	Fluorouracil 5%
2	Povidone Iodine 7.5%	36	Hydrocortisone Acetate 1%
3	Ketoconazole 2%	37	Hydroquinone 2%
4	Lindane 1%	38	Hydroquinone 4%
5	Aciclovir 5%	39	Imiquimod 5%
6	Adapalene 1%	41	Lidocaine/Prilocaine 2.5%/2.5%
7	Azelaic Acid 20%	42	Mafenide Acetate 112 mg/g
8	Betamethasone 0.1%	43	Miconazole Nitrate 2%
9	Calamine 8%	45	Monobenzone 20%
10	Clindamycin 2%	46	Nitrofurazone 0.2%
11	Clobetasol Propionate 0.05%	47	Nonoxynol-9 5%
12	Clotrimazole 1%	48	Acetaminophen 100 mg/ml
14	Clotrimazole 2%	49	Acetaminophen 120 mg/5ml
16	Permethrin 5%	50	Albendazole 200 mg/5ml
17	Phenytoin Sodium 1%	51	Aluminium Hydroxide 320 mg/5ml
18	Sertaconazole Nitrate 2%	52	Aluminium/Magnesium 225/200 mg/5ml
19	Silver Sulfadiazine 10 mg/g	53	Aluminium/Magnesium 225/200 mg/5ml, 10ml
20	Terbinafine Hydrochloride 1%	54	Aluminium/Magnesium/Simethicone
21	Tolnaftate 1%	55	Aluminium/Magnesium/Simethicone 10 ml
22	Tretinoin 0.05%	56	Beractant 25 mg/ml
23	Triamcinolone Acetonide 0.1%	57	Budesonide 0.5 mg/2ml
24	Triamcinolone NN*	58	Budesonide 1 mg/2ml
25	Triple Sulfa	59	Carbamazepine 100 mg/5ml
26	Urea 10%	60	Charcoal Activated 30 g/240ml
27	Urea 20%	61	Chloramphenicol 150 mg/5ml
28	Urea 5%	62	Clindamycin 75 mg/5ml
29	Conjugated Estrogens 0.625 mg/g	63	Phenytoin 30 mg/5ml
30	Crotamiton 10%	64	Primidone 125 mg/5ml
31	Dexpanthenol 5%	65	Primidone 250 mg/5ml
32	Dienestrol 0.01%	66	Pyrantel 250 mg/5ml
33	Dithranol 0.25%	67	Pyrvinium 50 mg/5ml
34	Fluocinolone Acetonide 0.025%	68	Co-trimoxazole 200/40 mg/5ml

Code	Drug name
69	Furazolidone 50 mg/15ml
70	Ibuprofen 100 mg/5ml
71	Magnesium Hydroxide 8%
72	Metronidazole 125 mg/5ml
73	Nalidixic Acid 60 mg/ml
74	Nevirapine 50 mg/ml
75	Nitrofurantoin 25 mg/5ml
76	Sulfasalazine 30 mg/ml, 100ml
77	Diazepam 10 mg
78	Diazepam 5 mg
79	Docusate Na & Sorbitol 0.01/13.4 g
80	Hydrocortisone 100 mg/60ml
81	Mesalazine 4 g/100ml
82	Piracetam 33.33%
83	Sevoflurane 250 mg
84	Levonorgestrel 6X36 mg
85	Acetaminophen 125 mg
86	Acetaminophen 325 mg
87	Antihemorrhoid
88	Artisunate 50 mg
90	Bisacodyl 10 mg
91	Bisacodyl 5 mg
92	Clindamycin 100 mg
93	Clotrimazole 100 mg
94	Clotrimazole 200 mg
95	Clotrimazole 500 mg
96	Piroxicam 20 mg
97	Povidone Iodine 200 mg
98	Progesterone 200 mg
99	Progesterone 400 mg
102	Diclofenac Sodium 100 mg
103	Diclofenac Sodium 50 mg

Code	Drug name
104	Glycerin 1 g
105	Glycerin 2 g
106	Hyoscine-n-butyl Bromide 10 mg
107	Hyoscine-n-butyl Bromide 7.5 mg
108	Indomethacin 100 mg
109	Indomethacin 50 mg
110	Mesalazine 500 mg
111	Metronidazole 500 mg
112	Miconazole Nitrate 100 mg
113	Morphine Sulfate 10 mg
115	Cetirizine Hydrochloride 5mg/5ml
116	Chloroquine 50 mg/5ml
117	Chloroquine 25 mg/5ml
118	Chlorpheniramine Maleate 2 mg/5ml
119	Cimetidine 200 mg/5ml
120	Piperazine Hexahydrate 750 mg/5ml
121	Promethazine 5 mg/5ml
122	Pseudoephedrine Hydrochloride 30 mg/5ml
123	Ranitidine 75mg/ml
124	Salbutamol 2 mg/5ml
125	Theophylline/Guaifenesin 50/30 mg/5ml
126	Valproate Sodium 200 mg/5ml
127	Valproate Sodium 300 mg/5ml
128	Zidovudine 50 mg/5ml
129	Dextromethorphan Hydrobromide 15 mg/5ml
130	Dextromethorphan/Pseudoephedrine 15/30 mg/5ml
131	Diphenhydramine/Ammonium chloride 12.5/125 mg
132	Ethosuximide 250 mg/5ml
133	Expectorant
134	Expectorant Codeine
135	Fluoxetine 20 mg/5ml
136	Guaifenesin 100 mg/5ml

Code	Drug name		Code	Drug name
137	Hydroxyzine Hydrochloride 10 mg/5ml		174	Cefixime 200 mg
138	Ipecac 0.14%		175	Cefixime 400 mg
139	Ketotifen 1 mg/5 ml		176	Celecoxib 100 mg
140	Lactulose 10 g/15 ml		177	Celecoxib 200mg
141	L-Carnitine 500 mg/5ml		178	Chloramphenicol 250 mg
142	Levamisole 40 mg/5ml		179	Ciclosporin 100 mg
143	Loratadine 5 mg/5ml		180	Ciclosporin 25 mg
144	Midazolam Hydrochloride 2 mg/ml		181	Ciclosporin 50 mg
145	Paromomycin 125 mg/ 5ml		182	Clindamycin 150 mg
146	Pediatric Grippe		183	Clofazimine 100 mg
148	Salicylic Acid 40 mg		184	Clofazimine 50 mg
149	Nitroglycerin 10 mg/24h		185	Clofibrate 500 mg
150	Nitroglycerin 5 mg/24h		186	Cloxacillin 250 mg
151	Acetaminophen 325 mg		187	Cloxacillin 500 mg
152	Acetaminophen 500 mg		188	Phenoxybenzamine Hydrochloride 10 mg
153	Acitretin 10 mg		189	Phenytoin Sodium 100 mg
154	Acitretin 25 mg		190	Phenytoin Sodium 50 mg
155	Adult Cold		191	Phosphocysteamine 150 mg
156	Adult Cold Preparations		192	Piroxicam 10 mg
157	Amantadine Hydrochloride 100 mg		193	Prednimustine 10 mg
158	Amoxicillin 250 mg		194	Prednimustine 50 mg
159	Amoxicillin 500 mg		195	Procainamide Hydrochloride 500 mg
160	Ampicillin 250 mg		196	Procarbazine 50 mg
161	Ampicillin 500 mg		198	Quinidine Sulfate 200 mg
164	Artemether 40 mg		200	Ribavirin 200 mg
165	Azithromycin 250 mg		201	Rifabutin 150 mg
167	Betacarotene 15 mg		202	Rifampicin 150 mg
168	Betacarotene 25 mg		203	Rifampicin 300 mg
169	Betacarotene 30 mg		204	Rivastigmine 1.5 mg
170	Buprenorphine 0.4 mg		205	Rivastigmine 4.5 mg
171	Calcitriol 0.25 mcg		206	Rivastigmine 6 mg
173	Cefalexin 500 mg		207	Rivastigmine 3 mg

Code	Drug name
208	Sertralin 100 mg
210	Simethicone 125 mg
212	Succimer 100 mg
213	Tacrolimus 0.5 mg
214	Tacrolimus 1 mg
215	Tacrolimus 5 mg
216	Tamsulosin Hydrochloride 0.4 mg
217	Testosterone Undecanoate 40 mg
218	Tetracycline Hydrochloride 250 mg
219	Theophylline 200 mg
220	Thiothixene 5 mg
221	Tiotropium 18 mcg
222	Tramadol Hydrochloride 50 mg
223	Tranexamic Acid 250 mg
224	Tropisetron 5 mg
225	Typhoid Vaccine
226	Ursodeoxycholic Acid 250 mg
227	Valproate Sodium 300 mg
228	Valsartan 160 mg
229	Valsartan 40 mg
230	Valsartan 80 mg
231	Vitamin A 25,000 U
232	Vitamin A 50,000 U
233	Vitamin D3 50,000 U
234	Zidovudine 100 mg
235	Zidovudine 300 mg
236	Zonisamide 100 mg
237	Cromolyn Sodium 20 mg
238	Cycloserine 250 mg
239	Cysteamine Bitartrate 150 mg
240	Danazol 100 mg
241	Danazol 200 mg

Code	Drug name
242	Dantrolene Sodium 25 mg
243	Dextromethorphan Hydrobromide 15 mg
244	Diazoxide 100 mg
245	Diazoxide 50 mg
248	Diltiazem Hydrochloride 120 mg
249	Diphenhydramine Hydrochloride 25 mg
250	Disopyramide 100 mg
251	Doxepin 10 mg
252	Doxepin 25 mg
253	Doxycycline 100 mg
254	Estramustine Sodium Phosphate 140 mg
255	Ethosuximide 250 mg
256	Etoposide 100 mg
257	Etoposide 50 mg
258	Fenofibrate 100 mg
259	Fenofibrate 200 mg
260	Ferrous-glycine-sulfate 567.66 mg
261	Ferrous-glycine-sulfate/Folic Acid 0
262	Fluconazole 100 mg
263	Fluconazole 150 mg
264	Fluconazole 200 mg
266	Fluoxetine 10 mg
267	Fluoxetine 20 mg
268	Flurazepam 15 mg
270	Gabapentin 100 mg
271	Gabapentin 300 mg
272	Gabapentin 400 mg
273	Gemfibrozil 300 mg
274	Hematinic
276	Hexamethylmelamine 100 mg
277	Hydroxyurea 250 mg
278	Hydroxyurea 500 mg

Code	Drug name		Code	Drug name
279	Ibuprofen 200 mg		311	Oseltamivir 75 mg
280	Ibuprofen 400 mg		312	Pancreatin Forte
281	Ibuprofen/Paracetamol/Caffeine		313	Pantoprazole 15 mg
282	Imatinib 100 mg		314	Paromomycin 250 mg
283	Imatinib 50 mg		315	Penicillamine 250 mg
284	Indomethacin 25 mg		316	A.C.A
285	Indomethacin 75 mg		317	Acarbose 100 mg
286	IODIDE [125I] (0.281-0.878) mCi		318	Acarbose 50 mg
287	Isotretinoin 10 mg		321	Acetaminophen Codeine 300/10 mg
288	Isotretinoin 20 mg		322	Acetazolamide 250 mg
289	Itraconazole 100 mg		323	Acetylcysteine 200 mg
290	Lithium Carbonate 400 mg		324	Acetylcysteine 600 mg
291	Lomustine 40 mg		325	Acetylsalicylic acid/Ascorbic acid 400/240 mg
292	Loperamide Hydrochloride 2 mg		326	Aciclovir 200 mg
293	Mebeverine Hydrochloride 200 mg		327	Aciclovir 400 mg
294	Mefenamic Acid 250 mg		328	Adefovir Dipivoxil 10 mg
295	Metoclopramide Hydrochloride 10 mg		334	Albendazole 200 mg
296	Mexiletine 100 mg		335	Albendazole 400 mg
297	Multivitamin Therapeutic		336	Alendronate 10 mg
298	Mycophenolate Mofetil 250 mg		337	Alendronate 35 mg
299	Naltrexone Hydrochloride 25 mg		338	Alendronate 70 mg
300	Naltrexone Hydrochloride 50 mg		339	Allopurinol 100 mg
301	Nifedipine 10 mg		340	Allopurinol 300 mg
302	Nifedipine 20 mg		341	Alprazolam 0.5 mg
303	Nifedipine 30 mg		342	Alprazolam 1 mg
304	Nitroglycerin 2.6 mg		343	Aluminium Hydroxide 300 mg
305	Nitroglycerin 0.4 mg		344	Aluminium/Magnesium 200/200 mg
306	Nitroglycerin 2.5 mg		346	Ambenonium Chloride 10 mg
307	Nitroglycerin 6.4 mg		347	Amiloride/Hyrochlorothiazide 5/50 mg
308	Nitroglycerin 6.5 mg		348	Aminoglutethimide 250 mg
309	Omeprazole 20 mg		349	Amiodarone Hydrochloride 200 mg
310	Orlistat 120 mg		350	Amitriptyline Hydrochloride 10 mg

Code	Drug name
351	Amitriptyline Hydrochloride 100 mg
352	Amitriptyline Hydrochloride 25 mg
353	Amitriptyline Hydrochloride 50 mg
354	Amlodipine 5 mg
355	Amlodipine/Atorvastatin 5/20 mg
356	Ammonium Chloride 500 mg
357	Amoxicillin 250 mg
358	Amoxicillin 500 mg
359	Amoxicillin 200 mg
360	Amoxicillin 400 mg
361	Anthocyanoside A
363	Antihistamine Decongestant
364	Artemether/Lumefantrine 20/120 mg
365	Artisunate 100 mg
366	ASA (Acetylsalicylic Acid) 100 mg
368	ASA (Acetylsalicylic Acid) 325 mg
371	ASA (Acetylsalicylic Acid) 500 mg
372	ASA (Acetylsalicylic Acid) 80 mg
373	ASA Codeine 500/10 mg
374	Aspartame 18 mg
375	Atenolol 100 mg
376	Atenolol 50 mg
377	Atorvastatin 10 mg
378	Atorvastatin 20 mg
379	Atorvastatin 40 mg
380	Atovaquone/Chloroghanide Hydrochloride 250/100 mg
381	Atovaquone/Chloroghanide Hydrochloride 62.5/25 mg
382	Atropine Sulfate 0.5 mg
383	Azathioprine 50 mg
385	Azithromycin 500 mg
386	Baclofen 10 mg
387	Baclofen 25 mg
388	Belladonna Pb
389	Benztropine Mesylate 2 mg
390	Betahistine Dihydrochloride 8 mg
391	Betamethasone 0.5 mg
392	Bethanechol Chloride 10 mg
393	Biperiden Hydrochloride 2 mg
394	Biperiden Hydrochloride 4 mg
396	Bismuth Subcitrate Equ. To Bismuth Oxide** 120 mg
397	Bromhexine Hydrochloride 8 mg
398	Bromocriptine 2.5 mg
400	Buprenorphine 2 mg
401	Buprenorphine 8 mg
402	Buprenorphine Hydrochloride/Naloxone Hydrochloride 2/0.5 mg
403	Buprenorphine Hydrochloride/Naloxone Hydrochloride 8/2 mg
404	Bupropion Hydrochloride 100 mg
405	Bupropion Hydrochloride 150 mg
406	Bupropion Hydrochloride 75 mg
407	Buspirone Hydrochloride 10 mg
408	Buspirone Hydrochloride 5 mg
409	Busulfan 2 mg
410	Cabergoline 0.5 mg
411	Cabergoline 1 mg
412	Calcium Acetate 667 mg
413	Calcium Dobesilate 250 mg
414	Calcium Pantothenate 100 mg
415	Capecitabine 150 mg
416	Capecitabine 500 mg
417	Captopril 25 mg
418	Captopril 50 mg
419	Carbamazepine 200 mg
421	Carbamazepine 400 mg
422	Carvedilol 12.5 mg

Code	Drug name
423	Carvedilol 25 mg
424	Carvedilol 6.25 mg
429	Cefuroxime 125 mg
430	Cefuroxime 250 mg
431	Cefuroxime 500 mg
432	Cetirizine Hydrochloride 5 mg
433	Cetirizine Hydrochloride 10 mg
434	Charcoal Activated 250 mg
435	Children Cold
436	Chlorambucil 2 mg
437	Chlordiazepoxide 10 mg
438	Chlordiazepoxide 5 mg
439	Chlormadinone Acetate/Ethinyl Estradiol 2 / 0.03 mg
440	Chloroquine Phosphate 250 mg
441	Chlorpheniramine Maleate 4 mg
442	Chlorpheniramine Maleate 8 mg
443	Chlorpromazine Hydrochloride 100 mg
444	Chlorpromazine Hydrochloride 25 mg
445	Chlorpropamide 250 mg
446	Chlorthalidone 100 mg
447	Cimetidine 200 mg
448	Cinnarizine 25 mg
449	Cinnarizine 75 mg
450	Ciprofloxacin 250 mg
451	Ciprofloxacin 500 mg
452	Cisapride 10 mg
453	Cisapride 5 mg
454	Citalopram 20 mg
455	Citalopram 40 mg
456	Clarithromycine 250 mg
457	Clarithromycine 500 mg
458	Clemastine 1mg

Code	Drug name
459	Clidinium/Chlordiazepoxide 2.5/5 mg
460	Clobazam 10 mg
461	Clobutinol Hydrochloride 40 mg
462	Clomiphene Citrate 50 mg
463	Clomipramine Hydrochloride 10 mg
464	Clomipramine Hydrochloride 25 mg
465	Clomipramine Hydrochloride 50 mg
466	Clomipramine Hydrochloride 75 mg
467	Clonazepam 1 mg
468	Clonazepam 2 mg
469	Clonidine Hydrochloride 0.2 mg
470	Clopidogrel 75 mg
475	Clozapine 100 mg
476	Clozapine 25 mg
477	Co-amoxiclav 250/125 mg
478	Co-amoxiclav 500/125 mg
479	Codeine Phosphate 30 mg
480	Colchicine 1 mg
481	Penicillin V 500 mg
482	Pentazocine Hydrochloride 50 mg
483	Pentoxifylline 400 mg
484	Perphenazine 2 mg
485	Perphenazine 4 mg
486	Perphenazine 8 mg
487	Phenazopyridine Hydrochloride 100 mg
488	Phenobarbital 100 mg
489	Phenobarbital 15 mg
490	Phenobarbital 60 mg
491	Phenytoin/Phenobarbital 100/50 mg
492	Phosphate, Potassium Monobasic* 500 mg
493	Phosphate, Sodium*
494	Pimozide 4 mg

Code	Drug name
495	Pioglitazone 15 mg
496	Pioglitazone 30 mg
497	Pioglitazone 45 mg
498	Piperazine Hexahydrate 500 mg
499	Piracetam 800 mg
500	Potassium Aminobenzoic Acid 500 mg
501	Potassium Chloride 500 mg
502	Potassium Chloride 600 mg
503	Potassium Citrate 10 mEq
504	Potassium Citrate 5 mEq
505	Pramipexole Hydrochloride 0.125 mg
506	Pramipexole Hydrochloride 0.25 mg
507	Pramipexole Hydrochloride 0.5 mg
508	Pramipexole Hydrochloride 1 mg
509	Praziquantel 600 mg
510	Prazosin 1 mg
511	Prazosin 5 mg
513	Prednimustine 100 mg
514	Prednimustine 20 mg
516	Prednisolone 5 mg
517	Prednisolone Forte 50 mg
518	Prednisone 1 mg
519	Prednisone 50 mg
520	Primaquine 15 mg
521	Primaquine 7.5 mg
522	Primidone 250 mg
523	Promethazine Hydrochloride 25 mg
524	Propafenone Hydrochloride 150 mg
525	Propafenone Hydrochloride 300 mg
526	Propantheline Bromide 15 mg
527	Propranolol Hydrochloride 20 mg
528	Propranolol Hydrochloride 10 mg

Code	Drug name
529	Propranolol Hydrochloride 160 mg
530	Propranolol Hydrochloride 40 mg
531	Propranolol Hydrochloride 80 mg
533	Propylthiouracil 50 mg
534	Prostaglandin E2 3 mg
535	Prothionamide 125 mg
536	Prothionamide 250 mg
537	Pseudoephedrine Hydrochloride 30 mg
538	Pyrantel 125 mg
539	Pyrazinamide 500 mg
540	Pyridostigmine Bromide 10 mg
541	Pyridostigmine Bromide 60 mg
543	Pyrimethamine 25 mg
544	Pyrvinium 50 mg
545	Quinacrine Hydrochloride 100 mg
546	Quinidine Bisulfate 250 mg
548	Quinine Hydrochloride 100 mg
549	Quinine Hydrochloride 250 mg
550	Quinine Sulfate 200 mg
551	Rabeprazole Sodium 20 mg
552	Raloxifene Hydrochloride 60 mg
553	Ranitidine 150 mg
555	Ranitidine 300 mg
556	Repaglinide 0.5 mg
557	Repaglinide 1 mg
558	Repaglinide 2 mg
559	Reserpine 0.1 mg
561	Rifabutin 300 mg
562	Riluzole 50 mg
563	Risperidone 1 mg
564	Risperidone 2 mg
565	Risperidone 3 mg

Code	Drug name	Code	Drug name
566	Risperidone 4 mg	600	Sumatriptan 50 mg
567	Ritodrine 10 mg	601	Tadalafil 10 mg
568	Rizatriptan 10 mg	602	Tadalafil 20 mg
569	Rizatriptan 5 mg	603	Tamoxifen 20 mg
570	Ropinirole 5 mg	604	Tamoxifen 10 mg
571	Ropinirole 0.25 mg	605	Terazosin 2 mg
572	Ropinirole 1 mg	606	Terazosin 5 mg
573	Salbutamol 2 mg	607	Terbinafine 250 mg
574	Selegiline Hydrochloride 5 mg	608	Terbutaline Sulfate 5 mg
575	Sertaconazole Nitrate 500 mg	609	Tetrabenazine 25 mg
577	Sertralin 25 mg	610	Theophylline 100 mg
578	Sertralin 50 mg	613	Thiabendazole 500 mg
579	Sildenafil 100 mg	614	Thiethylperazine 6.5 mg
580	Sildenafil 25 mg	615	Thioguanine 40 mg
581	Sildenafil 50 mg	616	Thioridazine Hydrochloride 10 mg
582	Simvastatin 10 mg	617	Thioridazine Hydrochloride 100 mg
583	Simvastatin 20 mg	618	Thioridazine Hydrochloride 25 mg
584	Sirolimus 1 mg	619	Ticlopidine Hydrochloride 250 mg
585	Sodium Aminosalicylate 500 mg	620	Tilactase 3000 FCC U
586	Sodium Salicylate 300 mg	621	Tinidazole 500 mg
587	Sotalol Hydrochloride 40 mg	622	Tizanidine 4 mg
588	Sotalol Hydrochloride 80 mg	623	Tolmetin 200 mg
589	Spiramycin 500 mg	624	Tolterodine Tartarate 1 mg
590	Spironolactone 100 mg	625	Tolterodine Tartarate 2 mg
591	Spironolactone 25 mg	626	Topiramate 100 mg
592	Stavudine 40 mg	627	Topiramate 200 mg
593	Sucralfate 1 g	628	Topiramate 25 mg
594	Sucralfate 500 mg	629	Topiramate 50 mg
595	Sulfadiazine 500 mg	630	Tramadol Hydrochloride 100 mg
596	Sulfadoxine/Pyrimethamine 500/25 mg	633	Tranexamic Acid 500 mg
597	Sulfasalazine 500 mg	634	Tranylcypromine 10 mg
599	Sumatriptan 100 mg	635	Trazodone Hydrochloride 50 mg

Code	Drug name
636	Triamterene/Hydrochlorothiazide 50/25 mg
637	Triclabendazole 250 mg
638	Trifluoperazine 1 mg
639	Trifluoperazine 10 mg
640	Trifluoperazine 2 mg
641	Trifluoperazine 5 mg
642	Trihexyphenidyl Hydrochloride 2 mg
643	Trihexyphenidyl Hydrochloride 5 mg
644	Trimethoprim 100 mg
645	Trimipramine 100 mg
646	Trimipramine 25 mg
649	Valproate Sodium 200 mg
650	Valproate Sodium 500 mg
651	Valproic acid (as Divalproex Sodium) 250 mg
652	Valproic acid (as Divalproex Sodium) 500 mg
656	Verapamil Hydrochloride 240 mg
657	Verapamil Hydrochloride 40 mg
658	Verapamil Hydrochloride 80 mg
661	Vitamin B1 100 mg
662	Vitamin B1 300 mg
663	Vitamin C 1000 mg
664	Vitamin E 200 IU
665	Vitamin K1 10 mg
666	Warfarin Sodium 2.5 mg
667	Warfarin Sodium 5 mg
668	Yohimbine Hydrochloride 2 mg
669	Yohimbine Hydrochloride 5.4 mg
670	Zafirlukast 20 mg
672	Zolpidem 10 mg
673	Zolpidem 5 mg
674	Conjugated Estrogens 0.625 mg
675	Conjugated Estrogens 1.25 mg

Code	Drug name
676	Contraceptive DE
677	Contraceptive HD
678	Contraceptive LD
679	Contraceptive LD/Fe
680	Contraceptive Triphasic
681	Co-trimoxazole 100/20 mg
682	Co-trimoxazole 400/80 mg
683	Co-trimoxazole 800/160 mg
684	Cumarin 100 mg
685	Cyclophosphamide 50 mg
687	Cyproheptadine Hydrochloride 4 mg
688	Cyproterone Acetate 50 mg
689	Cyproterone Compound
690	Dapsone 100 mg
691	Dapsone 50 mg
692	Deferasirox 125 mg
693	Deferasirox 250 mg
694	Deferasirox 500 mg
695	Deferiprone 500 mg
696	Desipramine Hydrochloride 25 mg
697	Desmopressin 120 mcg
698	Desmopressin 60 mcg
699	Desmopressin Acetate 0.1 mg
700	Desmopressin Acetate 0.2 mg
701	Dexamethasone 0.5 mg
702	Dextroamphetamine Sulfate 10 mg
703	Dextroamphetamine Sulfate 5 mg
704	Dextromethorphan Hydrobromide 15 mg
706	Diazepam 2 mg
709	Diclofenac Sodium 25 mg
711	Dicyclomine Hydrochloride 10 mg
712	Didanosine 200 mg

Code	Drug name
713	Diethylcarbamazine Citrate 50 mg
714	Digestive
715	Digoxin 0.25 mg
716	Dihydroergotamine Mesylate 2.5 mg
717	Dihydroergotoxine Mesylate 1.5 mg
718	Diloxanide Furoate 500 mg
720	Diltiazem Hydrochloride 60 mg
721	Dimenhydrinate 50 mg
722	Dimethicone 40 mg
724	Diphenoxylate
725	Dipyridamole 25 mg
726	Dipyridamole 75 mg
727	Donepezil Hydrochloride 10 mg
728	Donepezil Hydrochloride 5 mg
732	Drospirenone/Ethinyl Estradiol 3 / 0.03 mg
733	Dydrogesterone 10 mg
734	Dydrogesterone 5 mg
735	Enalapril Maleate 20 mg
736	Enalapril Maleate 5 mg
737	Ephedrine Hydrochloride 20 mg
738	Ergotamine Compound
739	Ergotamine Tartarate/Caffeine 1/100 mg
740	Ergotamine Tartrate 2 mg
741	Erythromycin 200 mg
743	Erythromycin 400 mg
744	Estradiol 25 mcg
745	Estradiol Valerate 1 mg
746	Estradiol Valerate 2 mg
747	Ethacrynic Acid 50 mg
748	Ethambutol Hydrochloride 400 mg
749	Ethinylestradiol 0.05 mg
750	Ethinylestradiol 0.5 mg

Code	Drug name
751	Ethionamide 250 mg
752	Etidronate Disodium 200 mg
753	Exemestane 25 mg
754	Ezetimibe 10 mg
755	Famotidine 20 mg
756	Famotidine 40 mg
757	Fexofenadine Hydrochloride 120 mg
758	Fexofenadine Hydrochloride 180 mg
759	Fexofenadine Hydrochloride 60 mg
760	Finasteride 1 mg
761	Finasteride 5 mg
762	Flecainide Acetate 100 mg
763	Fluconazole 50 mg
767	Flucytosine 500 mg
768	Fludrocortisone Acetate 0.1 mg
769	Fluoride 0.25 mg
770	Fluoride 1 mg
771	Fluoride Sodium 20 mg
772	Flupenthixol 0.5 mg
773	Flupenthixol 3 mg
774	Fluphenazine 1 mg
775	Fluphenazine 2.5 mg
776	Flutamide 250 mg
777	Fluvoxamine Maleate 100 mg
778	Fluvoxamine Maleate 50 mg
779	Folic Acid 1 mg
780	Folic Acid 5 mg
781	Fosfestrol 120 mg
782	Furazolidone 100 mg
783	Furosemide 40 mg
784	Galantamine 4 mg
785	Galantamine 8 mg

Code	Drug name		Code	Drug name
786	Galantamine 12 mg		822	Isosorbide Dinitrate 40 mg
787	Gemfibrozil 450 mg		823	Isosorbide Dinitrate 5 mg
788	Glibenclamide 5 mg		824	Isosorbide Mononitrate 60 mg
789	Gliclazide 30 mg		825	Isoxsuprine Hydrochloride 10 mg
790	Gliclazide 80 mg		826	Ketoconazole 200 mg
791	Granisetron 1 mg		827	Ketotifen 1 mg
792	Griseofulvin 125 mg		828	Lamivudine 100 mg
793	Griseofulvin 500 mg		829	Lamivudine 150 mg
794	Haloperidol 0.5 mg		830	Lamotrigine 100 mg
795	Haloperidol 2 mg		831	Lamotrigine 25 mg
796	Haloperidol 5 mg		832	Lamotrigine 50 mg
797	Hydralazine Hydrochloride 10 mg		833	L-Carnitine 1 g
798	Hydralazine Hydrochloride 25 mg		834	L-Carnitine 250 mg
799	Hydralazine Hydrochloride 50 mg		835	Letrozole 2.5 mg
800	Hydrochlorothiazide 50 mg		836	Leucovorin 15 mg
801	Hydrocortisone 10 mg		837	Leucovorin 25 mg
802	Hydroxychloroquine Sulfate 200 mg		838	Leucovorin 5 mg
803	Hydroxyzine Hydrochloride 10 mg		839	Levamisole 50 mg
804	Hydroxyzine Hydrochloride 25 mg		840	Levodopa 500 mg
808	Imatinib 100 mg		841	Levodopa/Benserazide 100/25 mg
809	Imipramine Hydrochloride 10 mg		842	Levodopa/Benserazide Forte 200/50 mg
810	Imipramine Hydrochloride 25 mg		843	Levodopa/Beserazide 50/12.5 mg
811	Imipramine Hydrochloride 50 mg		844	Levodopa/Carbidopa 100/10 mg
812	Indapamide 1.5 mg		845	Levodopa/Carbidopa 100/25 mg
814	Indomethacin 80 mg		846	Levodopa/Carbidopa Forte 250/25 mg
815	Iodoquinol 210 mg		847	Levonorgestrel 30 mcg
816	Iopanoic Acid 500 mg		848	Levonorgestrel 750 mcg
817	Isocarboxazid 10 mg		849	Levothyroxine Sodium 100 mcg
818	Isoniazid 100 mg		850	Levothyroxine Sodium 50 mcg
819	Isoniazid 300 mg		851	Liothyronine Sodium 25 mcg
820	Isoniazid/Prazinamide/Rifampin		852	Lisinopril 10 mg
821	Isosorbide Dinitrate 10 mg		853	Lisinopril 20 mg

Code	Drug name
854	Lisinopril 5 mg
855	Lithium Carbonate 300 mg
858	Loratadine 10 mg
860	Lorazepam 1 mg
861	Lorazepam 2 mg
862	Losartan Potassium 25 mg
863	Losartan Potassium 50 mg
864	Lovastatin 20 mg
865	Lynestrenol 0.5 mg
866	Magnesium Oxide 400 mg
867	Maprotiline Hydrcochloride 25 mg
868	Maprotiline Hydrcochloride 75 mg
869	Mebendazole 100 mg
870	Mebeverine Hydrochloride 135 mg
871	Medroxyprogesterone Acetate 250 mg
872	Medroxyprogesterone Acetate 5 mg
873	Mefloquine 250 mg
874	Megestrol Acetate 15 mg
875	Megestrol Acetate 20 mg
876	Megestrol Acetate 40 mg
877	Melphalan 2 mg
878	Memantine Hydrochloride 10 mg
879	Memantine Hydrochloride 5 mg
880	Mercaptopurine 50 mg
881	Mesalazine 250 mg
883	Mesalazine 400 mg
886	Mesalazine 800 mg
887	Metaproterenol Sulfate 20 mg
888	Metformin Hydrochloride 500 mg
889	Metformin Hydrochloride 1000 mg
890	Methadone Hydrochloride 20 mg
891	Methadone Hydrochloride 40 mg

Code	Drug name
892	Methadone Hydrochloride 5 mg
893	Methimazole 5 mg
894	Methocarbamol 500 mg
895	Methotrexate 2.5 mg
896	Methotrexate 5 mg
897	Methoxsalen 10 mg
898	Methyldopa 250 mg
899	Methylergonovine Maleate 0.125 mg
900	Methylphenidate Hydrochloride 10 mg
901	Methylphenidate Hydrochloride 20 mg
902	Methyltestosterone 25 mg
904	Metoprolol Succinate 190 mg
905	Metoprolol Succinate 23.75 mg
906	Metoprolol Succinate 47.5 mg
907	Metoprolol Succinate 95 mg
908	Metoprolol Tartrate 100 mg
909	Metoprolol Tartrate 50 mg
910	Metronidazole 250 mg
912	Metyrapone 250 mg
913	Minoxidil 10 mg
914	Misoprostol 100 mcg
915	Mitotane 500 mg
916	Moclobemide 150 mg
917	Montelukast 10 mg
918	Montelukast 5 mg
921	Mycophenolate Mofetil 500 mg
922	Mycophenolic acid 360 mg
923	Mycophenolic acid 180 mg
924	Nalidixic Acid 500 mg
925	Naproxen 250 mg
926	Naproxen 500 mg
927	Nelfinavir 250 mg

Code	Drug name		Code	Drug name
928	Nelfinavir 625 mg		968	Oxycodone Hydrochloride 30 mg
929	Neomycin Sulfate 500 mg		969	Oxycodone Hydrochloride 5 mg
930	Neostigmine Bromide 15 mg		970	Oxymetholone 50 mg
931	Nevirapine 200 mg		971	Pancreatin
932	Niacinamide 500 mg		972	Pantoprazole 20 mg
934	Nicotine 2 mg		973	Pantoprazole 40 mg
935	Nicotinic Acid 100 mg		975	Saccharin 13-16 mg
936	Nicotinic Acid 25 mg		976	Adapalene 0.1%
937	Nicotinic Acid 500 mg		977	Artificial Saliva
941	Nimodipine 30 mg		978	Artificial Tears
942	Nitrazepam 5 mg		979	Benzoyl Peroxide 10%
943	Nitrofurantoin 100 mg		980	Benzoyl Peroxide 5%
946	Nitroglycerin 2.6 mg		981	Clindamycin 1%
949	Nortriptyline 10 mg		982	Piroxicam 0.5%
950	Nortriptyline 25 mg		983	Povidone Iodine 10%
951	Nystatin 100,000 U		984	Prostaglandin E2
952	Nystatin 500,000 U		985	Tretinoin 0.025%
953	Ofloxacin 200 mg		988	Diclofenac Diethylammonium Salt Eq To diclofenac s 1%
954	Ofloxacin 300 mg		989	Erythromycin 2%
955	Olanzapine 10 mg		990	Erythromycin 4%
956	Olanzapine 15 mg		991	Erythromycin/Benzoyl 3/5 %
957	Olanzapine 2.5 mg		992	Estradiol 0.06%
958	Olanzapine 5 mg		993	Estradiol 500 mcg
959	Ondansetron 4 mg		994	Fluoride 1.23%
960	ORS		995	Isotretinoin 0.05%
961	Oxandrolone 2.5 mg		996	Lidocaine Hydrochloride 2%
962	Oxazepam 10 mg		997	Lidocaine/Chlorhexidine
963	Oxcarbazepine 150 mg		998	Metronidazole 0.75%
964	Oxcarbazepine 300 mg		1000	Acetic Acid
965	Oxcarbazepine 600 mg		1001	Aluminium Chloride
966	Oxybutynin Chloride 5 mg		1002	Aminobenzoic Acid
967	Oxycodone Hydrochloride 15 mg		1003	Ammonium Alum

Code	Drug name		Code	Drug name
1004	Ammonium Chloride		1036	Tartaric Acid
1005	Ammonium Hydroxide		1037	Titanium Dioxide
1006	Benzoic Acid		1038	Urea
1007	Benzoin Tincture		1039	White Wax
1008	Benzyl Benzoate		1040	Zinc Oxide
1009	Borax		1041	Zinc Sulfate
1010	Boric Acid		1042	Collodion
1011	Cade Oil		1043	Copper Sulfate
1012	Camphor		1044	Cream Base
1013	Castor Oil		1045	Cresol
1014	Citric Acid		1046	Dextrose
1015	Clindamycin		1047	Eosin
1016	Phenol		1048	Erythromycin
1017	Phosphate, Potassium Monobasic *		1049	Ethacridine Lactate
1018	Phosphate, Sodium Dibasic *		1050	Ethanol
1019	Phosphate, Sodium monobasic *		1052	Gentian Violet
1020	Podophyllin		1053	Glycerin
1021	Polysorbate 80		1054	Hydrochloric Acid
1022	Potassium Iodide		1055	Hydrogen Peroxide Concentrate
1023	Potassium Permanganate		1056	Ichthyol
1024	Propylene Glycol		1057	Iodine
1025	Resorcinol		1058	Iodine Glycerin 5%
1026	Salicylic Acid		1059	Isopropyl Alcohol
1027	Silver Nitrate		1060	Lactic Acid
1028	Sodium Bicarbonate		1061	Lactose
1029	Sodium Cellulose Phosphate		1062	Lanolin
1030	Sodium Chloride		1063	Menthol
1031	Sodium Citrate		1064	Mono-di-tri-chloroacetic Acid
1032	Sodium Thiosulfate		1066	Enflurane
1033	Starch		1067	Ether
1034	Stearic Acid		1068	Formoterol Fumarate 12 mcg
1035	Sulfur		1069	Formoterol Fumarate 4.5 mcg

Code	Drug name
1070	Formoterol Fumarate 9 mcg
1071	Halothane 250 ml/bottle
1072	Ipratropium Bromide 20 mcg/dose
1073	Ipratropium Bromide 40 mcg/dose
1074	Isoflurane
1075	Krypton [81mKr] Gas 4 mCi
1076	Krypton [81mKr] Gas 8 mCi
1077	Nitrous Oxide
1078	Budesonide 64 mcg/dose
1079	Buserelin 150 mcg/dose
1080	Calcitonin, Salmon 100 IU/dose
1081	Calcitonin, Salmon 200 IU/dose
1082	Phenylephrine Hydrochloride 0.25%
1083	Phenylephrine Hydrochloride 0.5%
1084	Sodium Chloride 0.65%
1085	Cromolyn Sodium 20 mg/ml
1086	Desmopressin Acetate 10 mcg/dose
1087	Naphazoline Hydrochloride 0.05%
1088	Nitroglycerin 400 mcg/dose
1089	Aminobenzoic Acid/Padimate
1090	Aminobezoic Acid 5%
1093	Benzyl Benzoate 25%
1097	Clotrimazole/Betamethasone Dipropionate 1 / 0.05 %
1098	Coal Tar 5%
1101	Copper and Zinc Sulfate
1103	Mequinol/Tretinoin 10/0.01%
1104	Benzydamine Hydrochloride 0.15%
1105	Fluoride Sodium 0.2%
1106	Atropine Sulfate 0.5%
1107	Atropine Sulfate 1%
1108	Betamethasone Disodium Phosphate 0.1%
1109	Betaxolol 0.5%

Code	Drug name
1110	Brimonidine Tartrate 0.2%
1111	Brinzolamide 1%
1112	Chloramphenicol 0.5%
1113	Ciprofloxacin 0.3%
1114	Citalopram 40 mg/ml
1115	Clobutinol Hydrochloride 60 mg/ml
1116	Phenol Glycerin 6.4%
1118	Phenylephrine Hydrochloride 0.50%
1119	Phenylephrine Hydrochloride 5%
1120	Phenylephrine Zinc
1121	Pilocarpine Hydrochloride 1%
1122	Pilocarpine Hydrochloride 2%
1123	Pilocarpine Nitrate 1%
1124	Pilocarpine Nitrate 2%
1125	Pilocarpine Nitrate 4%
1126	Pilocarpinye Hydrochloride 4%
1127	Poly Vinyl Alcohol 14 mg/ml
1128	Polymyxin B Sulfate/Neomycin Sulfate/Hydrocortison
1129	Prednisolone Acetate 1%
1130	Propamidine Isethionate 0.1%
1131	Rifampicin 153 mg/ml
1132	Sodium Chloride 0.65%
1133	Sodium Chloride 5%
1134	Sulfacetamide Sodium 10%
1135	Sulfacetamide Sodium 20%
1136	Tetracaine Hydrochloride 0.5%
1137	Trifluridine 1%
1138	Trimipramine 4%
1139	Tropicamide 0.5%, 10ml
1140	Vitamin A 50,000 U/ml
1141	Desmopressin Acetate 0.1 mg/ml
1142	Dexamethasone Phosphate 0.1%

Code	Drug name
1143	Dextromethorphan Hydrobromide 4 mg/ml
1144	Diclofenac Sodium 0.1%
1145	Digoxin 0.5 mg/ml
1146	Dimethicone 40 mg/ml
1147	Dorzolamide 2%
1148	Echothiophate Iodide 0.06%
1149	Echothiophate Iodide 0.125%
1150	Echothiophate Iodide 0.25%
1151	Edetate Sodium
1152	Epinephrine 1%
1153	Fluoride Sodium 1.1 mg/ml
1154	Fluoride Sodium 550 mcg/0.15ml
1155	Fluorometholone 0.1%
1156	Gentamicin 3 mg/ml
1157	Homatropine Hydrobromide 2%
1158	Idoxuridine 0.1%
1159	Iron 25 mg/ml
1160	Metoclopramide Hydrochloride 4 mg/ml, 15ml
1161	Naphazoline Hydrochloride 0.05%
1162	Naphazoline Hydrochloride 0.1%
1163	Naphazoline Hydrochloride/Antazoline Phosphate 0.05/0.5 %
1164	Naphazoline Nitrate 0.05%
1165	Naphazoline Nitrate 0.1%
1166	Natamycin 5%
1167	Ofloxacin 0.30%
1168	Povidone Iodine 3 mg/cm2
1169	Microfibrillar Collagen Hemostat 14.8 mg/cm3
1170	Microfibrillar Collagen Hemostat 15.5 mg/cm3
1171	Microfibrillar Collagen Hemostat 22.3mg/cm3
1173	Bephenium Hydroxynaphthoate 5 g/sachet
1174	Clarithromycin 250 mg/sachet
1175	Mesalazine 1g/sachet

Code	Drug name
1176	Methylcellulose
1177	Montelukast 4 mg/Sachet
1178	Para aminosalicylic acid 4 g
1179	Clonidine Hydrochloride 0.2 mg/24h
1180	Scopolamine 1 mg/72h
1181	Estradiol 100 mcg/24h
1182	Estradiol 25 mcg/24h
1183	Estradiol 50 mcg/24h
1184	Estradiol Hemihydrate 100 mcg/24h
1185	Estradiol Hemihydrate 25 mcg/24h
1186	Estradiol Hemihydrate 50 mcg/24h
1187	Fentanyle 100 mcg/h
1188	Fentanyle 75 mcg/h
1189	Fentanyle 25 mcg/h
1190	Fentanyle 50 mcg/h
1191	Nicotine 15 mg/16h
1192	Abciximab 2 mg/ml, 5ml
1193	Acetaminophen 10 mg/ml, 50ml
1194	Acetaminophen 150 mg/ml, 4ml
1195	Acetazolamide 500 mg
1196	Acetylcholine Chloride 1%
1197	Acetylcysteine 200 mg/ml
1198	Aciclovir 250 mg
1199	Aciclovir 500 mg
1200	Activated Prothrombin Complex (concentrated) 500 U
1201	Adenosine 3 mg/ml, 2ml
1202	Activated Prothrombin Complex (concentrated) 1000 U
1203	Albumin (Human) 20%, 100ml
1204	Albumin (Human) 20%, 10ml
1205	Albumin (Human) 20%, 250ml
1206	Albumin (Human) 20%, 500ml
1207	Albumin (Human) 20%, 50ML

Code	Drug name	Code	Drug name
1208	Albumin (Human) 5%, 100ML	1247	Arginine Hydrochloride
1209	Albumin (Human) 5%, 250ml	1248	Arsenic Trioxide 0.1%
1210	Albumin (Human) 5%, 500ml	1249	Artemether 80 mg
1211	Alfentanil 0.5 mg/ ml, 5ml	1250	Articaine Hydrochloride/Epinephrine 4/0.001 %
1212	Alfentanil 0.5 mg/ml, 2ml	1251	Articaine Hydrochloride/Epinephrine 4/0.002 %
1213	Alfentanil 0.5 mg/ml, 10ml	1252	Artisunate 200 mg
1214	Allergenic Extracts	1253	Asparaginase 10,000 U
1215	Amikacin 250 mg/ml, 2ml	1254	Atracurium Besylate 10 mg/ml, 2.5ml
1216	Amikacin 50 mg/ml, 2ml	1255	Atracurium Besylate 10 mg/ml, 5ml
1217	Amino Caproic Acid 250 mg/ml	1256	Atropine Sulfate 0.5 mg/ml, 1ml
1218	Amino Caproic Acid 400 mg/ml,10ml	1257	Atropine Sulfate 1 mg/ml, 10ml
1221	Aminoacid 10%	1258	Atropine Sulfate 10 mg/ml, 2ml
1223	Aminoacid 5%	1259	Atropine Sulfate 2.5 mg/ml, 0.8ml
1224	Aminophylline 25 mg/ml, 10ml	1262	BCG
1225	Amiodarone Hydrochloride 50 mg/ml, 3ml	1264	Benztropine Mesylate 1 mg/ml, 2ml
1226	Amphotericin B Liposome 50 mg	1265	Betamethasone
1227	Amphotericin-B 50 mg	1266	Betamethasone 4 mg/ml
1228	Ampicillin 1 g	1267	Biperiden Lactate 5 mg/ml
1231	Ampicillin/Sulbactam 1/0.5 g	1268	Bleomycin 15 U
1232	Ampicillin/Sulbactam 2/1 g	1270	Botulinum A Toxin
1233	Anti - D Immunoglobulin 250 mcg	1271	Botulism Monovalent
1235	Anti - D Immunoglobulin 300 mcg	1272	Botulism Polyvalent (A+B+E)
1237	Anti - D Immunoglobulin 50 mcg/ml, 2ml	1273	Bovactant 50 mg/1.2ml
1238	Antilymphocyte Immunoglobulin 20 mg/ml, 5ml	1274	Bretylium Tosilate 50 mg/ml, 10ml
1239	Antilymphocyte Immunoglobulin 50 mg/ml	1275	Bretylium Tosilate 50 mg/ml, 20ml
1240	Antiscorpion Aenom Serum	1276	Bretylium Tosilate 50 mg/ml, 2ml
1241	Antisnake Aenom Serum	1277	Bromhexine Hydrochloride 2 mg/ml, 2 ml
1242	Antithymocyte Immunoglobulin 25 mg	1278	Bupivacaine Hydrochloride 0.25%, 10ml
1243	Antithymocyte Immunoglobulin 50 mg/ml, 5ml	1279	Bupivacaine Hydrochloride 0.25%, 20ml
1244	Apomorphine Hydrochloride 10 mg/ml	1280	Bupivacaine Hydrochloride 0.5%, 10ml
1245	Aprotinin 10,000 KIU/ml	1281	Bupivacaine Hydrochloride 0.5%, 20ml
1246	Aprotinin 20,000 KIU/ml, 1ml	1282	Bupivacaine Hydrochloride 0.5%, 4ml

Code	Drug name
1283	Buprenorphine 0.3 m/ml, 1ml
1284	Buserelin 1 mg/ml, 5.5ml
1285	Busulfan 6 mg/ml, 10ml
1286	Calcitonin 100 IU/ml
1287	Calcitonin 50 IU/ml
1288	Calcium Chloride 100 mg/m, 10ml
1289	Calcium Fulinate 10 mg/ml, 10ml
1290	Calcium Fulinate 10 mg/ml, 5ml
1291	Calcium Fulinate 100 mg
1292	Calcium Gluconate 10%, 10ml
1293	Capreomycin 1g
1294	Carboplatin 10 mg/ml, 15ml
1295	Carboplatin 10 mg/ml, 45ml
1296	Carboplatin 10 mg/ml, 5ml
1297	Carboplatin 150 mg
1298	Carboplatin 50 mg
1299	Carboprost 250 mcg/ml, 1ml
1300	Carmustine 100 mg
1301	Casoni Test
1302	Caspofungin 50 mg
1303	Caspofungin 70 mg
1304	Cefazolin 1 g
1305	Cefazolin 250 mg
1306	Cefazolin 500 mg
1307	Cefepime 1 g
1308	Cefepime 2 g
1309	Cefepime 500mg
1310	Cefotaxime 1 g
1311	Cefotaxime 500 mg
1312	Ceftazidime 1 g
1313	Ceftazidime 2 g
1314	Ceftazidime 500 mg

Code	Drug name
1315	Ceftizoxime 1 g
1316	Ceftizoxime 500 mg
1317	Ceftriaxone 250 mg
1318	Ceftriaxone 500 mg
1319	Ceftriaxone 1 g
1320	Cefuroxime 1.5 g
1322	Cefuroxime 750 mg
1323	Cephalothin 1 g
1324	Cetrorelix 250 mcg
1325	Cetrorelix 3mg
1326	Chloramphenicol 1 g
1327	Chlormethine Hydrochlorid (Nitrogen mustard) 10 mg
1328	Chloroquine 30 mg/ml, 5ml
1329	Chloroquine 40 mg/ml, 5ml
1330	Chloroquine Sulfate 50 mg/ml, 5ml
1331	Chlorpheniramine Maleate 10 mg/ml, 1ml
1332	Chlorpromazine Hydrochloride 25 mg/ml, 2ml
1333	Choriogonadotropin Alfa 500 mcg/ml, 0.5ml
1334	Chorionic Gonadotrophin (Human) 1500 U
1335	Chorionic Gonadotrophin (Human) 500 U
1336	Chorionic Gonadotrophin (Human) 5000 U
1337	Ciclosporin 50 mg/ml
1338	Ciclosporin 50 mg/ml, 5ml
1339	Cimetidine 100 mg/ml, 2ml
1340	Ciprofloxacin 2 mg/ml, 100ml
1341	Cisatracurium 2 mg/ml, 10ml
1342	Cisatracurium 2 mg/ml, 2.5ml
1343	Cisatracurium 2 mg/ml, 5ml
1344	Cisatracurium 5 mg/ml
1345	Cisplatin 0.5 mg/ml, 100ml
1346	Cisplatin 0.5 mg/ml, 20ml
1347	Cisplatin 10 mg

Code	Drug name		Code	Drug name
1348	Cisplatin 50 mg		1382	Piperacillin 2 g
1349	Citicoline 125 mg/ml, 2ml		1383	Piperacillin Sodium/Tazobactam Sodium 2g/250mg
1350	Cladribine 1mg/ml, 10ml		1384	Piperacillin Sodium/Tazobactam Sodium 3g/375mg
1351	Cladribine 2 mg/ml, 5ml		1385	Piperacillin Sodium/Tazobactam Sodium 4g/500mg
1352	Clemastine 1 mg/ml, 2ml		1386	Piroxicam 20 mg/ml
1353	Clindamycin 150 mg/ml, 2ml		1387	Pneumococcal Vaccine
1354	Clobutinol Hydrochloride 10 mg/ml, 2ml		1388	Poliomyelitis Vaccine
1355	Cloxacillin 1g		1389	Polymyxin B Sulfate 500,000 U
1358	Penicillin G Benzathin 1,200,000 U		1390	Poractant Alfa 80 mg/ml
1359	Penicillin G Benzathine 600,000 U		1391	Potassium Chloride Concentrated 1 mEq/ml, 10ml
1360	Penicillin G Potassium 1,000,000 U		1392	Potassium Chloride Concentrated 2 mEq/ml, 10ml
1361	Penicillin G Potassium 5000000 U		1393	Potassium Chloride Concentrated 2 mEq/ml, 50ml
1362	Penicillin G Procaine 400,000 U		1394	Pralidoxime Chloride 1 g
1363	Penicillin G procaine 800,000 U		1395	Prilocaine/ Felypressin
1364	Penicillin G Sodium 5,000,000 U		1396	Procainamide Hydrochloride 100 mg/ml, 10ml
1365	Pentagastrin 0.25 mg/ml, 2ml		1397	Progesterone 25 mg/ml
1366	Pentamidine Isoethionate 300 mg		1398	Progesterone 50 mg/ml
1367	Pentazocine 30 mg/ml, 1ml		1399	Promethazine Hydrochloride 25 mg/ml, 1ml
1368	Perphenazine 5 mg/ml		1400	Promethazine Hydrochloride 25 mg/ml, 2ml
1369	Pethidine Hydrochloride 50 mg/ml, 1ml		1401	propofol 10 mg/ml, 100ml
1370	Pethidine Hydrochloride 50 mg/ml, 2ml		1402	Propofol 10 mg/ml, 20ml
1371	Phenobarbital (as Sodium) 200 mg/ml, 1ml		1403	propofol 10 mg/ml, 50ml
1372	Phenobarbital Sodium 100 mg/ml		1404	propofol 20mg/ml, 50ml
1373	Phenobarbital Sodium 200 mg/ml, 1ml		1405	Propranolol Hydrochloride 1 mg/ml
1374	Phenolsulphonphthalein 6 mg/ml		1406	Prostaglandin E1 0.5 mg/ml
1375	Phentolamine Mesylate 10 mg/ml		1407	Prostaglandin E1 20 mcg
1376	Phenylephrine Hydrochloride 10 mg/ml		1408	Prostaglandin E2 10 mg/ml, 0.5ml
1377	Phenytoin Sodium 50 mg/ml, 5ml		1409	Protamine Sulfate 1000 UAH/ml, 5ml
1378	Phosphate, Potassium		1410	Protirelin 0.2 mg/ml
1379	PHOSPHOROUS [32P] CHROMIC PHOSPHATE COLLOID 5 mCi		1411	Quinine 250 mg/ml
1380	Pipecuronium Bromide 4 mg		1412	Quinine Dihydrochloride 300 mg/ml, 2ml
1381	Piperacillin 1 g		1413	Rabies Immune globulin

Code	Drug name
1414	Rabies Vaccine
1415	Rabies Vero Vaccine
1416	Ranitidine 25 mg/ml, 2ml
1417	Remifentanil 1 mg
1418	Remifentanil 2 mg
1419	Remifentanil 5 mg
1420	Reteplase 10.4 U
1421	Ribaverin 100 mg
1422	Rifampicin 600 mg
1423	Ringer Lactate 1000 ml
1424	Ringer Lactate 500 ml
1425	Ringers 1000 ml
1426	Ringers 250 ml
1427	Ringers 500 ml
1428	Ritodrine 10 mg/ml, 5ml
1429	Rituximab 10 mg/lm, 10ml
1430	Rituximab 10mg/ml, 50ml
1431	Rubella Rirus Vaccine
1432	Salbutamol 0.5 mg/ml, 1ml
1433	Sarcoidosis Test
1434	Schick Test
1435	Secretin 100 U
1436	Sodium Bicarbonate 8.4%, 10ml
1437	Sodium Bicarbonate 8.4%, 50ml
1438	Sodium Chloride 0.45%, 1000ml
1439	Sodium Chloride 0.45%, 500ml
1440	Sodium Chloride 0.9%, 1000ml
1441	Sodium Chloride 0.9%, 100ml
1442	Sodium Chloride 0.9%, 10ml
1443	Sodium Chloride 0.9%, 500ml
1444	Sodium Chloride 0.9%, 5ml

Code	Drug name
1446	Sodium Chloride 5%, 500ml
1447	Sodium Chloride 5%, 50ml
1448	Sodium Lactate 1/6 molar
1449	Sodium Nitrite 30 mg/ml, 10ml
1450	Sodium Tetradecyl Sulfate 1%, 2ml
1451	Sodium Tetradecyl Sulfate 3%, 2ml
1452	Sodium Thiosulfate 250 mg/ml, 50ml
1453	Somatostatin 0.25 mg
1454	Somatropin 4 U
1455	Somatropin 12 U
1456	Spectinomycin 2 g
1457	Stibogluconate Sodium 100 mg/ml
1458	Streptokinase 250,000 IU
1459	Streptokinase 750,000 IU
1460	Streptomycin 1 g
1461	Streptozocin 1 g
1462	Succinylcholine Chloride 1 g
1463	Succinylcholine Chloride 100 mg
1464	Succinylcholine Chloride 100 mg/ml, 10ml
1465	Succinylcholine Chloride 20 mg/ml
1466	Succinylcholine Chloride 50 mg/ml, 10ml
1467	Succinylcholine Chloride 50 mg/ml, 2ml
1468	Succinylcholine Chloride 500 mg
1469	Sufentanil 5 mcg/ml, 2ml
1470	Sufentanil 5 mcg/m, 5ml
1471	Sufentanil 5 mcg/ml, 10ml
1472	Sumatriptan 12 mg/ml, 0.5ml
1473	Tacrolimus 5 mg/ml, 1ml
1474	Technetium [99mtc] Pertechnetate 100 mCi
1475	Technetium [99mtc] Pertechnetate 200 mCi
1476	Technetium [99mtc] Pertechnetate 300 mCi

1445	Sodium Chloride 0.90% , 250ml	1477	Technetium [99mtc] Pertechnetate 400 mCi

Code	Drug name	Code	Drug name
1478	Technetium [99mtc]]ethylenedicystein (10 - 100) mCi	1510	Thallium [201Tl] Chloride 5 mCi
1479	Technetium [99mtc]antimony Sulfur Colloid (5 - 50) mCi	1511	Thiethylperazine 6.5 mg/ml
1480	Technetium [99mtc]bicisate (10 - 100) mCi	1512	Thiopental Sodium 1 g
1481	Technetium [99mtc]etidronate (up to 150) mCi	1513	Thiopental Sodium 25 mg/ml, 20ml
1482	Technetium [99mtc]exzametazine (10 - 30) mCi	1514	Thiopental Sodium 50 mg/ml, 20ml
1483	Technetium [99mtc]macrosalb (5 - 30) mCi	1515	Thiopental Sodium 500 mg
1484	Technetium [99mtc]mebrofenin (up to 100) mCi	1516	Thyrotrophin 10 U
1485	Technetium [99mtc]medronate (up to 300) mCi	1517	Tirofiban 0.25mg/ml, 50ml
1486	Technetium [99mtc]glucoheptonate (up to 150) mCi	1518	Tirofiban Hydrochloride 0.25mg/ml, 25ml
1487	Technetium [99mtc]mertiatide (10 - 100) mCi	1519	Tobramycin 10 mg/ml, 2ml
1488	Technetium [99mtc]pentate (up to 300) mCi	1520	Tobramycin 40 mg/ml, 2ml
1489	Technetium [99mtc]phytate (up to 100) mCi	1521	Tobramycin 50 mg / ml, 1.5ml
1490	Technetium [99mtc]pyrophosphate (up to 80) mCi	1522	Tolazoline Hydrochloridel 10 mg
1491	Technetium [99mtc]sestamibi (20-100) mCi	1523	Trace Metal Combination 1
1492	Technetium [99mtc]stannous Agent labelled Cells (10-100) mCi	1524	Tramadol Hydrochloride 50 mg/ml, 1ml
1493	Technetium [99mtc]succimer (up to 40) mCi	1525	Tranexamic Acid 100 mg/ml, 10ml
1494	Technetium [99mtc]sulfur Colloid (10-100) mCi	1526	Tranexamic Acid 100 mg/ml, 5ml
1495	Technetium [99mtc]tin Colloid (up to 100) mCi	1527	Tranexamic Acid 50 mg/ml, 5ml
1496	Technetium[99mtc]pentavalent Succimer (20-80) mCi	1528	Trastuzumab 150 mg
1497	Teicoplanin 200 mg	1529	Trastuzumab 440 MG
1498	Teicoplanin 400 mg	1530	Triamcinolone Acetonide 40 mg/ml, 1ml
1499	Teniposide 10 mg/ml, 5ml	1531	Trifluoperazine 1 mg/ml
1500	Terbutaline Sulfate 0.5 mg/ml	1532	Trimethaphan Camsylate 50 mg/ml, 10ml
1501	Teriparatide 250 mcg/ml, 3ml	1533	Triptorelin 3.75 mg
1502	Testosterone Enantate 100 mg/ml	1534	Triptorelin (as Acetate) 95.6 mcg
1503	Testosterone Enantate 250 mg/ml	1535	Tropisetron 1 mg/ml, 5ml
1504	Tetanus Antitoxin uine*	1536	Trypan Blue 0.1%
1505	Tetanus Immune globulin* 250 IU	1537	Tuberculin ppd*
1506	Tetanus Toxoid Adsorbed	1538	Urofollitropin 75 IU FSH
1507	Tetracaine Hydrochloride 1%	1539	Urokinase 250,000 IU

1508	Tetracosactide Acetate 1 mg/ml
1509	Thallium [201Tl] Chloride 10 mCi
1540	Urokinase 75,000 IU
1541	Valproate Sodium 100 mg/ml, 10ml

Code	Drug name
1542	Valproate Sodium 100 mg/ml, 3ml
1543	Valproate Sodium 100 mg/ml, 5ml
1544	Valproate Sodium 400 mg
1545	Vancomycin 500 mg
1546	Varicella Vaccine
1547	Vasoperssin 20 pressorU/ml
1548	Verapamil Hydrochloride 2.5 mg/ml, 2ml
1549	Vinblastine Sulfate 10 mg
1550	Vincristine Sulfate 1 mg
1551	Vincristine Sulfate 1 mg/ml
1552	Vindesine Sulfate 5 mg
1553	Vinorelbine 10 mg/ml, 1ml
1554	Vinorelbine 10 mg/ml, 5ml
1556	Vitamin B Complex
1557	Vitamin B12 100 mcg/ml
1558	Vitamin B2 5 mg/ml, 2ml
1559	Vitamin B6 100 mg/ml, 3ml
1560	Vitamin B6 50 mg/ml, 2ml
1561	Vitamin C 100 mg/ml, 5ml
1562	Vitamin D3 300,000 U
1563	Vitamin E 100 IU/ml
1564	Vitamin K1 10 mg/ml
1565	Vitamin K1 2 mg/ml, 0.5ml
1566	Water 10 ml
1567	Water 2 ml
1568	Water 3 ml
1569	Water 5 ml
1570	Yellow Fever Vaccine
1571	Conjugated Estrogens 5 mg/ml, 5ml
1575	Cyclophosphamide 200 mg
1576	Cyclophosphamide 500 mg
1577	Cytarabine 1 g
1578	Cytarabine 100 mg
1579	Cytarabine 20 mg/ml, 50ml
1580	Cytarabine 20 mg/ml, 5ml
1581	Dacarbazine 100 mg
1582	Dacarbazine 200 mg
1583	Dacarbazine 500 mg
1584	Daclizumab 5 mg/ml, 5ml
1585	Dactinomycin 0.5 mg
1586	Dalteparin Sodium 10000 U/ml
1587	Dalteparin Sodium 12500 U/ml
1588	Dalteparin Sodium 25000 U/ml
1589	Dalteparin Sodium 7500 U/0.3 ml
1590	Danaparoid Sodium 1250 U/ml, 0.6ml
1591	Dantrolene Sodium 20 mg
1592	Daunorubicin 20 mg
1593	Deferoxamine Mesylate 2 g
1594	Deferoxamine Mesylate 500 mg
1595	Dehydroemetine Dihydrochloride 30 mg/ml
1596	Desmopressin Acetate 15 mcg/ml
1597	Desmopressin Acetate 4 mcg/ml
1598	Desoxycorticosterone Acetate 5 mg/ml
1599	Dexamethasone Phosphate 4 mg/ml, 2ml
1600	Dextran/ NaCl 500 ml
1601	Dextrose 10%, 1lit
1602	Dextrose 10%, 500ml
1603	Dextrose 20% , 50ml

1572	Corticotrophin 40 U/ml	1604	Dextrose 20%, 1lit
1573	Corticotrophin 80 U/ml	1605	Dextrose 20%, 500ml
1574	Co-trimoxazole 400/80 mg/5ml	1606	Dextrose 5% , 250ml

Code	Drug name	Code	Drug name
1607	Dextrose 5%, 1lit	1640	Doxorubicin Hydrochloride 2 mg/ml, 25ml
1608	Dextrose 5%, 500ml	1641	Doxorubicin Hydrochloride 2 mg/ml, 5ml
1609	Dextrose 50%, 1lit	1642	Doxorubicin Hydrochloride 50 mg
1610	Dextrose 50%, 500ml	1643	Droperidol 2.5 mg/ml, 10ml
1611	Dextrose 50%, 50ml	1644	Droperidol Compound
1612	Dextrose 70%, 1lit	1645	Edetate Calcium Disodium 50 mg/ml
1613	Dextrose/ Ehthanol 5/5%, 1000ml	1646	Edetate Dicobalt 15 mg/ml, 20ml
1614	Dextrose/ NaCl 5/0.45%, 1000ml	1647	Edetate Disodium 150 mg/ml
1615	Dextrose/ NaCl 5/0.45%, 500ml	1648	Edrophonium Chloride 10 mg/ml
1616	Dextrose/ NaCl 5/0.9%, 1000ml	1649	Enoxaparin Sodium 100 mg/ml, 0.2 ml
1617	Dextrose/ NaCl 5/0.9%, 250ml	1650	Enoxaparin Sodium 100 mg/ml, 0.4 ml
1618	Dextrose/ NaCl 5/0.9%, 500ml	1651	Enoxaparin Sodium 100 mg/ml, 0.6 ml
1619	Diazepam 5 mg/ml, 2ml	1652	Enoxaparin Sodium 100 mg/ml, 0.8 ml
1620	Diclofenac Sodium 25 mg/ml, 3ml	1653	Enoxaparin Sodium 100 mg/ml, 1 ml
1621	Dicyclomine Hydrochloride 10 mg/ml, 2ml	1654	Ephedrine Hydrochloride 50 mg/ml
1622	Digoxin 0.25 mg/ml, 2ml	1655	Ephedrine Sulfate 50 mg/ml
1623	Digoxin Specific Antibody 40 mg	1656	Epinephrine 1 mg/ml
1624	Dihydroergotamine Mesylate 1 mg/ml	1657	Epinephrine 1 mg/ml, 2ml
1625	Diltiazem Hydrochloride 100 mg	1658	Epirubicin Hdrochloride 2mg/ml, 25ml
1627	Dimercaprol 100 mg/ml, 3ml	1659	Epirubicin Hdrochloride 2mg/ml, 5ml
1628	Diphenhydramine Hydrochloride 50 mg/ml	1660	Epirubicin Hydrochloride 10 mg
1629	Diphteria & Tetanus toxoids Adsorbed (Dt)(for Pedi	1661	Epirubicin Hydrochloride 50 mg
1630	Diphteria & Tetanus toxoids Adsorbed (Td)(for adul	1662	Erythromycin 1 g
1631	Diphteria Antitoxin Equine	1663	Erythropoetin 2000 U/0.3ml
1632	Diphteria Toxoid/Tetanus Toxoid/Pertussis Vaccine	1664	Erythropoietin 10,000 U/ml
1633	Dipyridamole 5 mg/ml, 2ml	1665	Erythropoietin 1000 U/ml
1634	Dobutamine 12.5 mg/ml, 20ml	1666	Erythropoietin 20,000 U/ml
1635	Docetaxel 20 mg	1667	Erythropoietin 2000 U/ml

1636	Docetaxel 80 mg	1668	Erythropoietin 4000 U/0.3ml
1637	Dopamine Hydrochloride 40 mg/ml, 5ml	1669	Erythropoietin 4000 U/ml
1638	Doxapram Hydrochloride 20 mg/ml, 5ml	1670	Esmolol Hydrochloride 250 mg/ml, 10ml
1639	Doxorubicin Hydrochloride 10 mg	1671	Estradiol Valerate 10 mg/ml

Code	Drug name	Code	Drug name
1672	Etanercept 25 mg	1704	Fosfestrol 50 mg/ml, 5ml
1673	Ethiodized Oil 10ml	1705	Furosemide 10 mg/ml, 25ml
1674	Etomidate 2 mg/ml, 10ml	1706	Furosemide 10 mg/ml, 2ml
1675	Etoposide 20 mg/ml, 10ml	1707	Furosemide 10 mg/ml, 4ml
1676	Etoposide 20 mg/ml, 5ml	1708	Gadodiamide 287 mg/ml
1677	Factor IX Complex 1,000 IU	1709	Gadopentetate Dimeglumine 469 mg/ml
1678	Factor IX Complex 250 IU	1710	Gallium[67ga]citrate 10 mCi
1679	Factor IX Complex 500 IU	1711	Gallium[67ga]citrate 5 mCi
1680	Factor VII	1712	Ganciclovir 500 mg
1681	Factor VIII 250 U	1713	Ganirelix 500 mcg/ml, 0.5ml
1682	Factor VIII 500 U	1714	Gaseous Gangrene Antitoxin
1683	Fentanyl 50 mcg/ml, 10ml	1715	Gelatin Modified
1684	Fentanyle 50 mcg/ml, 10ml	1716	Gelatin Modified 4%
1685	Fentanyle 50 mcg/ml, 2ml	1717	Gemcitabine Hydrochloride 1 g
1686	Fentanyle 50 mcg/ml, 5ml	1718	Gemcitabine Hydrochloride 200 mg
1687	Ferric Oxide Saccharated 20 mg/ml	1719	Gentamicin 40 mg/ml, 1ml
1688	Fibrinogen 1 g	1720	Gentamicin 40 mg/ml, 2ml
1689	Filgrastim 300 mcg/ml, 1ml	1721	Gentamicin 10 mg/ml, 2ml
1690	Filgrastim 600 mcg/ml, 0.5ml	1722	Gestonorone Caproate 100 mg/ml, 2ml
1691	Fludarabine Phosphate 50 mg	1723	Glucagon 1 mg
1692	Flumazenil 0.1 mg/ml, 5ml	1724	Glycopyrronium Bromide 200 mcg/ml, 1ml
1693	Fluorescein Sodium 10%	1725	Glycopyrronium Bromide 200 mcg/ml, 5ml
1694	Fluorouracil 50 mg/ml	1726	Gold Sodium Thiomalate 50 mg/ml, 10ml
1695	Flupenthixol Decanoate 20 mg/ml	1727	Gold Sodium Thiomalate 100 mg/ml, 0,5ml
1696	Fluphenazine Decanoate 25 mg/ml	1728	Gold Sodium Thiomalate 100 mg/ml, 10ml
1697	Folic Acid 5 mg/ml, 10ml	1729	Gold Sodium Thiomalate 100 mg/ml, 1ml
1698	Follitropin Alfa 600 IU/ml, 0.5ml	1730	Gold Sodium Thiomalate 20 mg/ml, 0.5ml

1699	Follitropin Alfa 600 U/ml, 0.75ml	1731	Gold Sodium Thiomalate 20 mg/ml, 10ml
1700	Follitropin Alfa 600 U/ml, 1.5ml	1732	Gold Sodium Thiomalate 20 mg/ml, 1ml
1701	Follitropin Alfa 75 IU	1733	Gold Sodium Thiomalate 40 mg/ml, 0.5ml
1702	Follitropin Beta 75 IU	1734	Gold Sodium Thiomalate 40 mg/ml, 10ml
1703	Fomepizole 1 g/ml, 1.5ml	1735	Gold Sodium Thiomalate 40 mg/ml, 1ml

Code	Drug name	Code	Drug name
1736	Gold Sodium Thiomalate 50 mg/ml, 0.5ml	1769	Imipramine Hydrochloride 12.5 mg/ml, 2ml
1737	Gold Sodium Thiomalate 50 mg/ml, 1ml	1770	Immune Globulin
1738	Gonadorelin 100 mcg	1771	Immune Globulin 50 mg/ml, 200ml
1739	Gonadorelin Acetate 0.8 mg	1772	Immune Globulin 50mg/ml, 100ml
1740	Gonadorelin Acetate 3.2 mg	1773	Immune Globulin 50mg/ml, 10ml
1741	Granisetron 1 mg/ml, 3ml	1774	Immune Globulin 50mg/ml, 50ml
1742	Granisetron 1mg/ml, 1ml	1775	Indocyanine Green 25 mg
1743	Haloperidol 5 mg/ml	1776	Infliximab 100 mg
1744	Haloperidol 50 mg/ml	1777	Influenza Virus
1745	Heparin Sodium 10,000 U/ml, 1ml	1778	Insulin (Regular) 100 IU/ml
1746	Heparin Sodium 100 IU/ml	1779	Insulin Aspart 100 U/ml, 3ml
1747	Heparin Sodium 5000 U/ml, 1ml	1781	Insulin Biphasic Isophane 100 IU/ml, 3ml
1748	Heparin Sodium 5000 U/ml, 5ml	1783	Insulin Biphasic isophane 100 IU/ml
1749	Hepatitis B immune globulin At least 200 IU/ml	1784	Insulin Glargine 100 IU/ml, 10ml
1750	Hepatitis B Virus	1785	Insulin Glargine 100 IU/ml, 3ml
1751	Hydralazine Hydrochloride 20 mg	1786	Insulin Isophane 100 IU/ml
1753	Hydrocortisone 50 mg/ml, 2ml	1787	Insulin Zinc 100 IU/ml
1754	Hydrocortisone 100 mg	1788	Interferons
1755	Hydrocortisone 50 mg/ml, 2ml	1789	Iodixanol 150 mgI/ml
1756	Hydroxyethylstarch 6% (130/04)	1790	Iodixanol 270 mgI/ml
1757	Hydroxyprogesteron Caproate 250 mg/ml	1791	Iodixanol 320 mgI/ml
1758	Hyoscine-n-butyl Bromide 20 mg/ml	1792	Iohexol 240 mgI/ml, 100ml
1759	Idarubicin Hydrochloride 10 mg	1793	Iohexol 240 mgI/ml, 10ml
1760	Idarubicin Hydrochloride 5 mg	1794	Iohexol 240 mgI/ml, 200ml
1761	Ifosfamide 1 g	1795	Iohexol 240 mgI/ml, 20ml
1762	Ifosfamide 2 g	1796	Iohexol 240 mgI/ml, 50ml

1763	Igm-enriched Human (Immune globulin)*	1797	Iohexol 300 mgI/ml, 100ml
1764	Imiglucerase 200 U	1798	Iohexol 300 mgI/ml, 10ml
1765	Imiglucerase 400 U	1799	Iohexol 300 mgI/ml, 200ml
1766	Imipenem /Cilastatin 250/250 mg	1800	Iohexol 300 mgI/ml, 20ml
1767	Imipenem /Cilastatin 500/500 mg	1801	Iohexol 300 mgI/ml, 50ml
1768	Imipenem /Cilastatin 750/750 mg	1802	Iohexol 350 mgI/ml, 100ml

Code	Drug name	Code	Drug name
1803	Iohexol 350 mgI/ml, 200ml	1835	Leuprorelin Acetate 3.75 mg
1804	Iohexol 350 mgI/ml, 20ml	1836	Lidocaine Dextrose 5/7.5 %
1805	Iohexol 350 mgI/ml, 50ml	1837	Lidocaine Epinephrine
1806	Iopamidol 300 mgI/ml, 100ml	1838	Lidocaine Epinephrine 2%
1807	Iopamidol 300 mgI/ml, 20ml	1839	Lidocaine Hydrochloride 1%, 50ml
1808	Iopamidol 300 mgI/ml, 50ml	1840	Lidocaine Hydrochloride 1%, 5ml
1809	Iopamidol 370 mgI/ml, 100ml	1842	Lidocaine Hydrochloride 2%, 5ml
1810	Iopamidol 370 mgI/ml, 20ml	1843	Lidocaine Hydrochloride 20%
1811	Iopamidol 370 mgI/ml, 50ml	1844	Lidocaine Hydrochloride 4%, 50ml
1812	Iopromide 240 mgI/ml, 20ml	1845	Lidocaine Hydrochloride 2%, 50ml
1813	Iopromide 240 mgI/ml, 50ml	1846	Lipid Infusion 10%
1814	Iopromide 300 mgI/ml, 10ml	1847	Lipid Infusion 20%
1815	Iopromide 300 mgI/ml, 20ml	1848	Lorazepam 2 mg/ml
1816	Iopromide 300 mgI/ml, 50ml	1849	Lorazepam 4 mg/ml
1817	Iopromide 370 mgI/ml, 100ml	1850	Lutropin Alfa 75 IU
1818	Iopromide 370 mgI/ml, 50ml	1851	Magnesium Sulfate 10%, 10ml
1819	Iotroxate Meglumine 50 mgI/ml, 100ml	1852	Magnesium Sulfate 10%, 50ml
1820	Irinotecan Hydrochioride 20 mg/ml, 5ml	1853	Magnesium Sulfate 20%, 10ml
1821	Irinotecan Hydrochloride 20 mg/ml, 2ml	1854	Magnesium Sulfate 20%, 50ml
1822	Irinotecan Hydrochloride 100MG, 5ML	1855	Magnesium Sulfate 50%, 10ml
1823	Irinotecan Hydrochloride 40mg, 2ML	1856	Magnesium Sulfate 50%, 50ml
1824	Iron 50 mg/ml, 5ml	1857	Mannitol 10%
1825	Iron 20 mg/ml, 5ml	1858	Mannitol 20%
1826	Isoniazid 100 mg/ml, 10ml	1859	Measles Vaccine
1827	Isoproterenol Hydrochloride 1 mg/ml, 2ml	1860	Measles and rubella Vaccine

1828	Isoproterenol Hydrochloride 0.2 mg/ml	1861	Measles Immune globulin 200 IU/ml
1829	Ketamine 50 mg/ml, 10 ml	1862	Measles, Mumps, and rubella Vaccine
1830	L-Carnitine 200 mg/ml, 5ml	1863	Medroxyprogesterone Acetate 100 mg/ml, 5ml
1831	Leucovorin 10 mg/ml, 3ml	1864	Medroxyprogesterone Acetate 150 mg/ml
1832	Leucovorin 3 mg/ml	1865	Medroxyprogestrone Acetate/Estradiol Cypionate 50/10 mg/ml, 0.5 ml
1833	Leucovorin 30 mg	1866	Meglumine Antimonate 300 mg/ml, 5ml
1834	Leucovorin 125/ml, 5ml	1867	Meglumine Compound 60%, 20ml

Code	Drug name	Code	Drug name
1868	Meglumine Compound 76%, 100 ml	1900	Methylergonovine Maleate 0.2 mg/ml
1869	Meglumine Compound 76%, 20ml	1901	Methylprednisolone 1000 mg
1870	Meglumine Gadoterate 377 mg/ml, 10ml	1902	Methylprednisolone 250 mg
1871	Meglumine Gadoterate 377 mg/ml, 15ml	1903	Methylprednisolone 40 mg
1872	Meglumine Gadoterate 377 mg/ml, 20ml	1904	Methylprednisolone 500 mg
1873	Meglumine/ Sodium ioxaglate 320 10 ml	1905	Methylprednisolone Acetate 40 mg/ml, 1ml
1874	Meglumine/ Sodium ioxaglate 320 20 ml	1906	Metoclopramide 5 mg/ml, 2ml
1875	Meglumine/Sodium Ioxaglate 320 100 ml	1907	Metoprolol Tartrate 1mg/ml, 5ml
1876	Meglumine/Sodium Ioxaglate 320 200 ml	1908	Metronidazole 5 mg/ml, 100ml
1877	Meglumine/Sodium Ioxaglate 320 50 ml	1909	Midazolam 5 mg/ml, 1ml
1878	Melphalan	1910	Midazolam 5 mg/ml, 2 ml
1879	Meningococcal Vaccine	1911	Midazolam 5 mg/ml, 3 ml
1880	Menotropins	1912	Midazolam 1 mg/ml, 5ml
1881	Mepivacaine Hydrochloride 2%, 20ml	1913	Milrinone 1 mg/ml, 10ml
1882	Mepivacaine Hydrochloride 3%, 1.7ml	1914	Milrinone 1 mg/ml, 20ml
1883	Mepivacaine Hydrochloride 3%, 1.8ml	1915	Mitomycin 10 mg
1884	Meropenem 1 g	1916	Mitomycin 2 mg
1885	Meropenem 500 mg	1917	Mitomycin 5 mg
1886	Mesna 100 mg/ml, 4ml	1918	Mitoxantrone 2 mg/ml, 10ml
1887	Metaproterenol Sulfate 0.5 mg/ml	1919	Mivacurium Chloride 2 mg/ml
1888	Methadone Hydrochloride 10 mg/ml	1920	Molgramostim 0.15 mg
1889	Methadone Hydrochloride 5 mg/ml	1921	Molgramostim 0.4 mg
1890	Methocarbamol 100 mg/ml, 10ml	1922	Morphine Sulfate 10 mg/ml
1891	Methotrexate 100 mg/ml	1923	Mumps Mirus Vaccine

1892	Methotrexate 5 mg
1893	Methotrexate 5 mg/ml
1894	Methotrexate 50 mg
1895	Methotrexate 1 g
1896	Methotrexate 10 mg/ml, 5ml
1897	Methotrexate 2.5 mg/ml
1898	Methotrexate 25 mg/ml
1899	Methylene Blue 10 mg/ml, 10 ml

1924	Nafcillin Sodium 1 g
1925	Nafcillin Sodium 500 mg
1926	Naloxone Hydrochloride 0.4 mg/ml
1927	Nandrolone Decanoate 25 mg/ml
1928	Nandrolone Phenpropionate 25 mg/ml
1929	Neostigmine Methylsulfate 2.5 mg/mlL, 5ml
1930	Neostigmine Methylsulfate 0.5 mg/ml
1931	Neostigmine Methylsulfate 2.5 mg/ml, 1ml

Code	Drug name
1932	Nimodipine 0.2 mg/ml
1933	Nitroglycerin 1 mg/ml, 10ml
1934	Nitroglycerin 1 mg/ml, 2ml
1935	Nitroglycerin 1 mg/ml, 5ml
1936	Nitroglycerin 5 mg/ml, 10ml
1937	Nitroglycerin 5 mg/ml, 1ml
1938	Nitroglycerin 5 mg/ml, 2ml
1939	Nitroprusside Sodium 50 mg
1940	Norepinephrine Bitartrate 0.1%
1941	Obidoxime Chloride 250 mg/ml
1942	Obidoxime Chloride 275 mg/ml, 0.8ml
1943	Octreotide 100 mcg/ml
1944	Octreotide 20 mg
1945	Octreotide 200 mcg/ml
1946	Octreotide 50 mcg/ml
1947	Ondansetron 2 mg/ml, 2ml
1948	Ondansetron 2 mg/ml, 4ml
1949	Oxaliplatin 100 mg
1950	Oxaliplatin 5 mg/ml, 10ml
1951	Oxaliplatin 5 mg/ml, 20ml
1952	Oxaliplatin 50 mg
1953	Oxytocin 10 U/ml
1954	Oxytocin 5 U/ml, 1ml

Code	Drug name
1964	Pamidronate Disodium 90 mg
1965	Pamidronate Disodium 9mg/ml, 10ml
1966	Pancuronium Bromide 2 mg/ml, 2ml
1968	Papaverine Hydrochloride 40 mg/ml
1969	Parathormon 100 uspU/ml
1970	Patent Blue V 2.5%, 2ml
1971	Pegaspargase 3750 IU
1972	Peginterferon Alfa-2a 360 mcg/ml, 0.5ml
1973	Peginterferon Alfa-2a 180 mcg/ml
1974	Peginterferon Alfa-2b 100 mcg
1975	Peginterferon Alfa-2b 120 mcg
1976	Peginterferon Alfa-2b 150 mcg
1977	Peginterferon Alfa-2b 50 mcg
1978	Peginterferon Alfa-2b 80 mcg
1979	Penicillin 6.3.3
1980	Aciclovir 3%
1983	Bacitracin 500 U/g
1984	Benzocaine 5%
1987	Burn Ointment
1988	Calcipotriol 50 mcg/g
1991	Simple Eye Oint*
1993	Tacrolimus 0.03%
1994	Tetracycline Hydrochloride 1%

1955	Paclitaxel 6 mg/ml, 5ml		1995	Tetracycline Hydrochloride 3%
1956	Paclitaxel 6 mg/ml, 16.7ml		1998	Vitamin A 250 U/g
1957	Paclitaxel 6 mg/ml, 25ml		1999	Vitamin A+D
1958	Paclitaxel 6 mg/ml, 50ml		2000	Zinc Oxide 20%
1959	Pamidronate Disodium 15 mg/ml, 2ml		2001	Erythromycin 0.5%
1960	Pamidronate Disodium 15 mg/ml, 4ml		2002	Fibrinolysin
1961	Pamidronate Disodium 15 mg/ml, 6ml		2004	Gentamicin 3 mg/g
1962	Pamidronate Disodium 3 mg/ml, 10ml		2007	Ichthyol 10%
1963	Pamidronate Disodium 30 mg		2008	Lidocaine/Hydrocortisone Acetate 5/0.5 %

Code	Drug name		Code	Drug name
2009	Menthol Salicylate		2053	Sorbitol 70%
2010	Mequinol 10%		2055	Terbutaline Sulfate
2011	Methyl Salicylate 30%		2056	Timolol 0.5%
2012	Mupirocin 2%		2058	Tropicamide 1%, 10ml
2014	Nitroglycerin 2%		2060	Cromolyn Sodium 10 mg/ml
2015	Nystatin 100,000 U/g		2061	Cromolyn Sodium 20 mg/2ml
2016	Paromomycin/Urea		2063	Cyclopentolate Hydrochloride 1%
2017	Fluorescein Sodium 1 mg		2064	Dextrose 5%, 100ml
2019	Bromhexine Hydrochloride 4 mg/5ml		2065	Diazepam 2 mg/5ml
2020	Dexamethasone 0.5 mg/5ml		2066	Dihydrotachysterol 0.25 mg/ml
2021	Dicyclomine Hydrochloride 10 mg/5ml		2067	Dimethyl Sulfoxide 50%
2022	Digoxin 0.05 mg/ml		2068	Diphenhydramine Hydrochloride 12.5 mg/5ml
2026	Amyl Nitrite		2071	Galantamine 20 mg/5ml
2028	Balanced Salt Solution		2072	Gentian Violet 1%
2029	Balanced Salt Solution/Glutathion		2073	Glycine 1.5%
2030	Calcipotriol 50 mcg/ml		2074	Haloperidol 2 mg/ml
2031	Carbon [14C] Urea 5 micCi		2075	Hemodialysis Concentrated I
2032	Cardioplegic Solution		2076	Hemodialysis Concentrated I,without Dextrose
2033	Cetrimide/Chlorhexidine		2077	Hemodialysis Concentrated II
2034	Chlorhexidine Gluconate/Detergent 4%		2078	Hemodialysis Concentrated II ,Acidic
2035	Ciclosporin 100 mg/ml		2079	Hemodialysis Concentrated III
2036	Ciclosporin 2%		2080	Iopamidol 300mgI/ml

2037	Clindamycin 10 mg/ml		2081	Ipratropium Bromide 250 mcg/ml, 1ml
2039	Peritoneal Dialysis		2082	Ipratropium Bromide 250 mcg/ml, 2ml
2042	Phosphate [32p] Sodium 5 mCi		2083	Lamivudine 10 mg/ml
2043	Poliovirus Vaccine		2084	Lamotrigine 10 mg/ml
2046	Povidone Iodine 2.5%		2085	Latanoprost 50 mcg/ml
2048	Salbutamol 5 mg/ml		2087	Lidocaine 10 mg/dose
2049	Salbutamol 2.5 mg/2.5ml		2088	Lidocaine Hydrochloride 4%
2050	Salicylic Acid Compound		2089	Loperamide Hydrochloride 1 mg/5ml
2051	Sodium Chloride 0.9%		2090	Megestrol Acetate 40 mg/ml
2052	Sodium Hypochlorite 0.55% (AVAILABLE Cl)		2091	Meglumine Compound 76%

Code	Drug name		Code	Drug name
2092	Methadone Hydrochloride 25 mg/5ml		2125	Charcoal Activated 50 g
2093	Methadone Hydrochloride 5mg/5ml		2126	Cholestyramine 4 g
2094	Methoxsalen 1%		2127	Clarithromycine 125 mg/5ml
2095	Minoxidil 20 mg/ml		2128	Co-amoxiclav 125/31.25 mg
2096	Minoxidil 50 mg/ml		2129	Co-amoxiclav 125/31.25 mg/5ml
2097	Neomycin Sulfate 125 mg/5ml		2130	Co-amoxiclav 200/28.5 mg/5ml
2098	Nicotine 10 mg/ml, 10ml		2131	Co-amoxiclav 250/62.5 mg
2099	Ondansetron 4 mg/5ml		2132	Co-amoxiclav 250/62.5 mg/5ml
2100	Ophthalmic Bath Solution		2133	Co-amoxiclav 400/57 mg/5ml
2102	Aluminium/Magnesium 564/174 mg		2134	Penicillin V 125 mg/5ml
2103	Amoxicillin 125 mg/5ml		2135	Penicillin V 250 mg/5ml
2104	Amoxicillin 200 mg/5ml		2136	Penicillin V Benzathine 200,000 IU/5ml
2105	Amoxicillin 250 mg/5ml		2137	Penicillin V Benzathine 400,000 IU/5ml
2106	Amoxicillin 400 mg/5ml		2139	Poly Ethylene Glycol 0
2107	Ampicillin 125 mg/5ml		2140	Poly Ethylenglycol - electrolyte 1*
2108	Ampicillin 250 mg/5ml		2141	Poly Ethylenglycol - electrolyte 2*
2109	Anticoagulant		2142	Potassium Aminobenzoic Acid 3 g/sachet
2110	Azithromycin 100 mg/5ml		2143	Potassium Chloride 20 mEq/sachet
2111	Azithromycin 200 mg/5ml		2144	Potassium Citrate 10 mEq/Sachet
2112	Barium Sulfate 135 g		2145	Sodium Polystyrene Sulfonate 454 g
2113	Bicarbonate Concentrate 500 mg		2146	Sorbitol 5 g/sachet

2114	Bicarbonate Concentrate 650 mg	2147	Sucralfate 1 g/sachet
2115	Bicarbonate Concentrate 750 mg	2148	Vancomycin 500 mg/6ml
2116	Cefalexin 125 mg	2149	Zanamivir 5 mg/Blister
2117	Cefalexin 125 mg/5ml	2151	Erythromycin 200 mg/5ml
2118	Cefalexin 250 mg	2152	Lactulose 10 g
2119	Cefalexin 250 mg/5ml	2153	Low Sodium Salt 5 g/sachet
2120	Cefixime 100 mg	2154	Methadone Hydrochloride 1g/ SUCHET
2121	Cefixime 100 mg/5ml	2155	Nelfinavir 50 mg/g
2122	Cefixime 50 mg	2156	Nystatin 100,000 U/ml
2123	Cefuroxime 125 mg/5ml	2158	Oseltamivir 60 mg/5ml
2124	Cefuroxime 250 mg/5ml	2164	Beclomethasone Dipropionate 50 mcg/dose

Code	Drug name	Code	Drug name
2165	Budesonide 200 mcg/Inhalation	2222	Pramipexole 0.7 Mg
2166	Budesonide 100 mcg/Inhalation	2223	Pramipexole 1 Mg
2167	Budesonide 400 mcg/Inhalation	2224	Pramipexole 1.5 Mg
2168	Salbutamol 100 mcg/dose	2225	Livergol 70 mg
2169	Salbutamol/Beclomethasone Dipropionate 100/50 mcg	2226	Livergol 140 mg
2170	Salmeterol 25 mcg/dose	2227	Valproate sodium 250 mg
2171	Salmeterol 50 mcg/dose	2228	vitamin C 250 mg
2172	Salmeterol / Fluticasone Propionate 25/125	2229	Calcium + vitamin D
2173	Salmeterol / Fluticasone Propionate 25/250	2230	Bisoprolol 1.25mg
2174	Salmeterol / Fluticasone Propionate 25/50	2231	Bisoprolol 1mg
2175	Cromolyn Sodium 1 mg/dose	2232	Bisoprolol 2.5mg
2176	Fluticasone 125 mcg/dose	2233	Bisoprolol 5mg
2177	Fluticasone 250 mcg/dose	2234	Sitagliptin 25mg
2179	Fluticasone 50 mcg/dose	2235	Sitagliptin 50mg
2180	Ipratropium bromide / Salbutamol 20/100 mcg/dose	2236	Sitagliptin 100mg
2181	Lidocaine Hydrochloride 6.5%		
2206	Pregabalin 25 Mg		
2207	Pregabalin 50 Mg		
2208	Pregabalin 75 Mg		
2209	Pregabalin 100 Mg		

2210	Pregabalin 150 Mg			
2211	Pregabalin 200 Mg			
2212	Pregabalin 225 Mg			
2213	Pregabalin 300 Mg			
2214	Vitamin E 100 IU			
2215	Vitamin E 400 IU			
2216	Pramipexole 0.088 Mg			
2217	Pramipexole 0.18 Mg			
2218	Pramipexole 0.25 Mg			
2219	Pramipexole 0.35 Mg			
2220	Pramipexole 0.375 Mg			
2221	Pramipexole 0.75 Mg			

PERSIAN Cohort
